# Publisher Correction: Intramolecular chaperone-mediated secretion of an Rhs effector toxin by a type VI secretion system

**DOI:** 10.1038/s41467-020-16325-2

**Published:** 2020-05-06

**Authors:** Tong-Tong Pei, Hao Li, Xiaoye Liang, Zeng-Hang Wang, Guangfeng Liu, Li-Li Wu, Haeun Kim, Zhiping Xie, Ming Yu, Shuangjun Lin, Ping Xu, Tao G. Dong

**Affiliations:** 1 State Key Laboratory of Microbial Metabolism, Joint International Research Laboratory of Metabolic & Developmental Sciences, School of Life Sciences and Biotechnology, Shanghai Jiao Tong University, 200240 Shanghai, China; 2 Department of Ecosystem and Public Health, University of Calgary, 3330 Hospital Dr. NW, Calgary, AB T2N4Z6 Canada; 3 National Center for Protein Science Shanghai, Shanghai Advanced Research Institute, Chinese Academy of Sciences, 201204 Shanghai, China

**Keywords:** Proteases, Bacterial secretion, Bacterial toxins, Pathogens

Correction to: *Nature Communications* 10.1038/s41467-020-15774-z, published online 20 April 2020.

The original version of this Article contained an error in Fig. 4c. A label in the ‘Elution’ panel (second row, second column) was incorrectly labelled with a ‘−‘ sign rather than a ‘+’ sign. This has now been corrected in both the PDF and HTML versions of the Article.

