# Supplementary material for: Intramolecular chaperone-mediated secretion of an Rhs effector toxin by a type VI secretion system
Source: Nat Commun. 2020 Apr 20;11:1865. doi: 10.1038/s41467-020-15774-z (PMC7170923; doi:10.1038/s41467-020-15774-z)
Supplement: Supplementary file 1 — Supplementary Information [file 41467_2020_15774_MOESM1_ESM.pdf]

Supplementary Information

Intramolecular chaperone-mediated secretion of an Rhs effector toxin by a type VI secretion system

Pei et al.

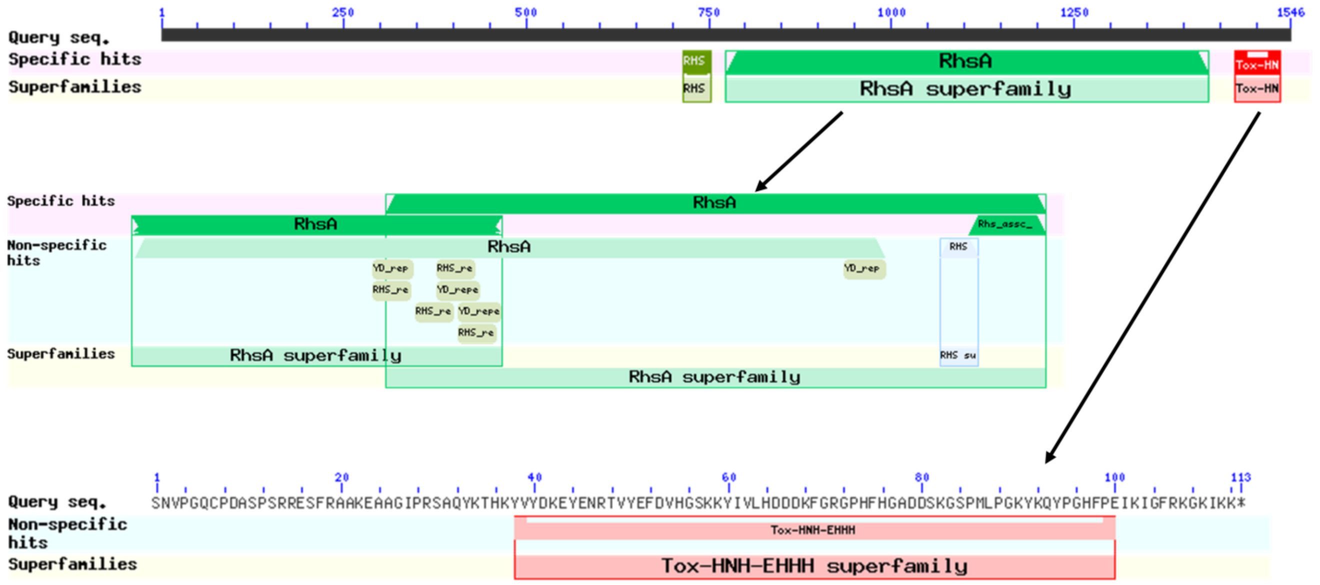

Supplementary Figure 1. Domain organization of TseI. TseI sequence was analyzed using BLAST CD-search (https://www.ncbi.nlm.nih.gov/Structure/cdd/wrpsb.cgi).

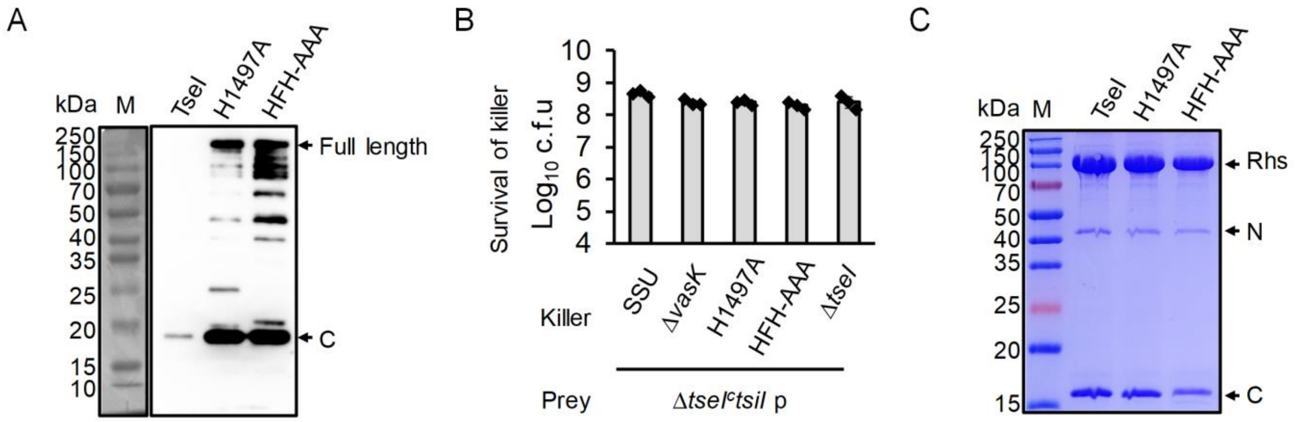

Supplementary Figure 2. Comparison of TseI and its non-toxic mutants for expression and toxicity assays. A, Western blotting analysis confirming plasmid-borne TseI and its catalytic mutants tested in Figure 1C were expressed. Full-length wild type TseI was barely detected, likely due to low abundance resulting from toxicity. **B,** Survival of killer strains in the competition assay of Figure 1D showing equal survival of killers. Error bars indicate the mean +/- standard deviation of three biological replicates. **C,** SDS-PAGE analysis of purified TseI and its catalytic mutant proteins used for the enzymatic assay for Figure 1E. Source data are provided as a Source Data file. Data in (A-C) are representative of at least two replications.

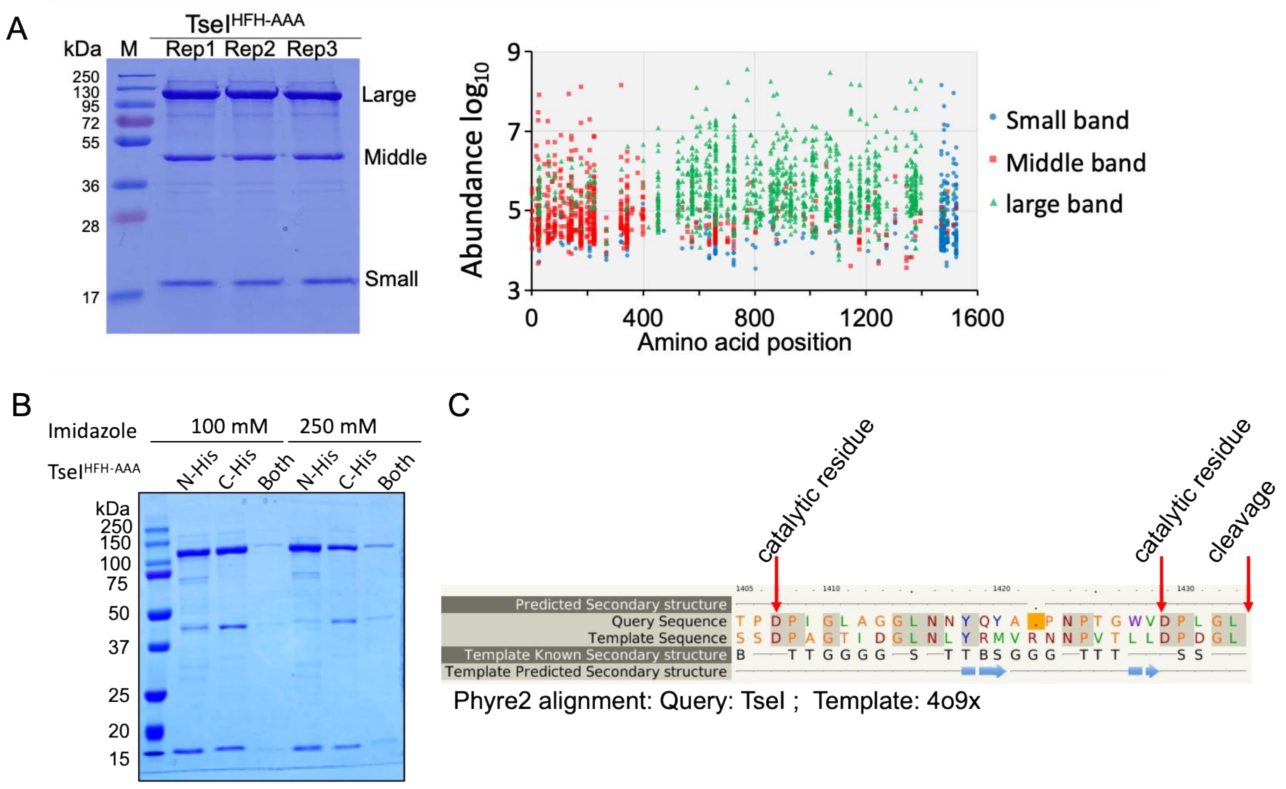

Supplementary Figure 3. Analysis of purified TseI cleaved fragments. A, Identification of TseI fragments by mass-spectrometry. Purified non-toxic TseI$^{HFH\text{-}AAA}$ mutant was resolved on an SDS-PAGE gel. The three major bands were excised individually and subject to N-terminal Edman sequencing (see Figure 2A) and LC-MS/MS analysis. The abundance of identified peptides was $\log_{10}$-transformed and plotted against the mapped location of TseI protein sequence. **B,** Comparison of eluted TseI proteins with N/C-terminal His-tag. Three major bands were observed regardless of the position of His-tag, suggesting the cleaved products remain in complex. **C,** Alignment of TseI C-terminal cleavage site with the Tc-toxin 4O9X. Conserved internal protease catalytic residues and cleavage sites are indicated. Phyre2 alignment between TseI and the template ends at the cleavage site as indicated. Data in (A-B) are representative of at least two replications.

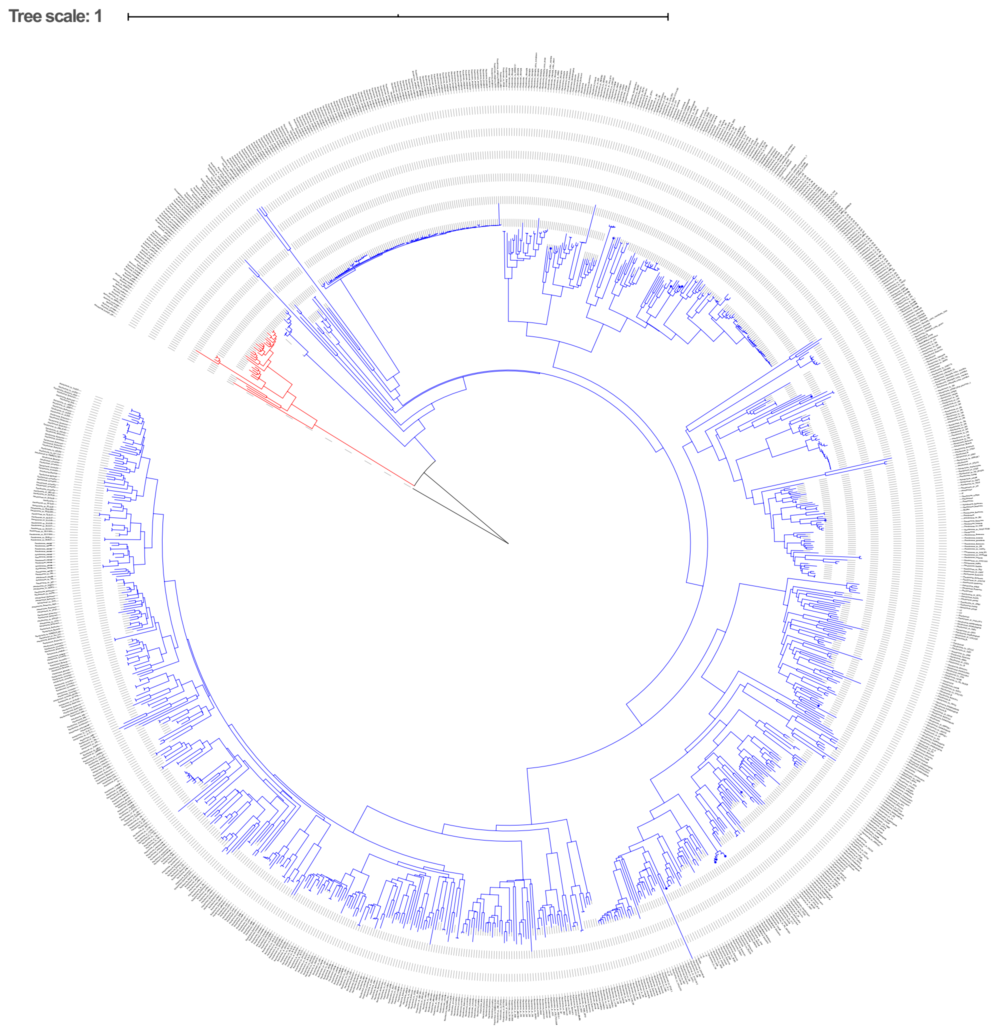

Supplementary Figure 4. Phylogeny of top 1000 blastp hits. Phylogeny was generated using the NCBI distance tree of results web function and visualized using the iTOL server. Sequences from *Aeromonas* species are highlighted in red while those from *Pseudomonas* sequences in blue.

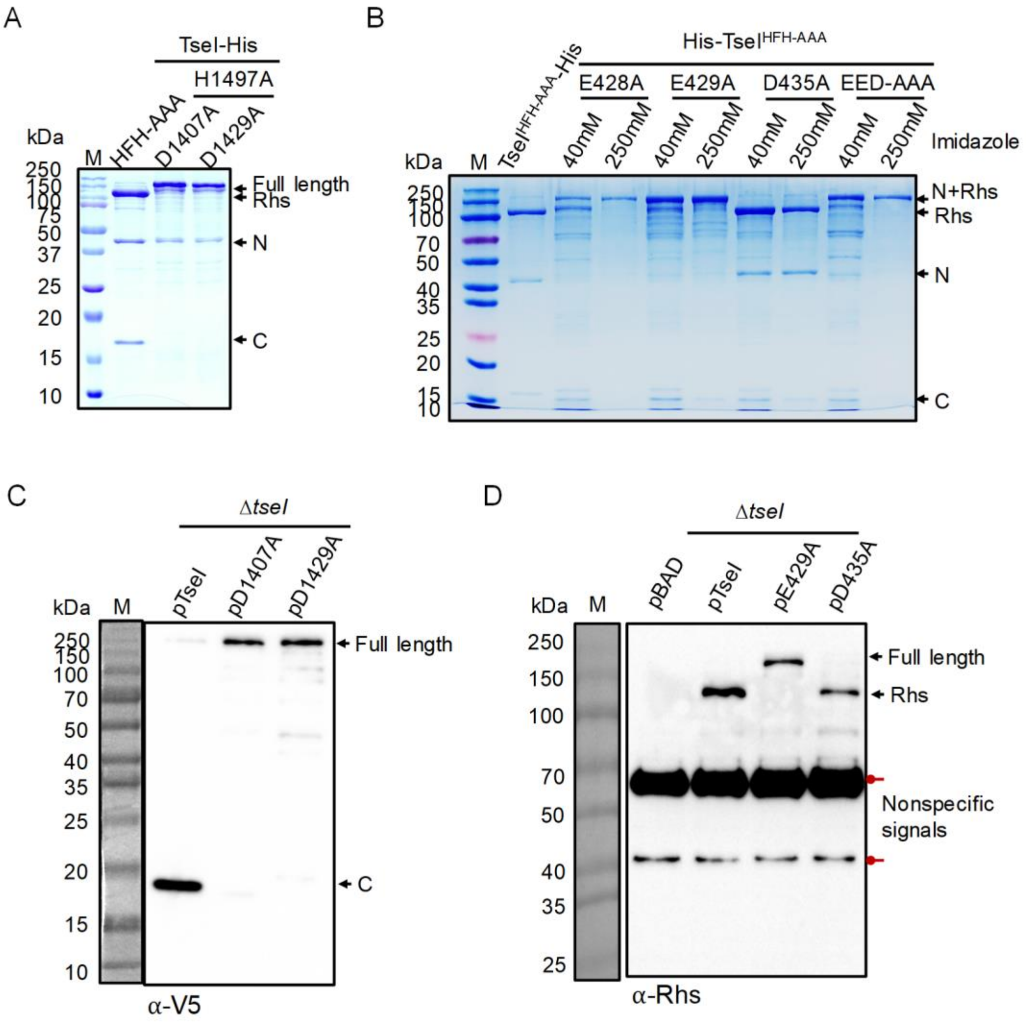

Supplementary Figure 5. Identification of critical residues that dictate TseI cleavage. A-B, Protein purification of cleavage-deficient TseI mutants. TseI nontoxic HFA-AAA and H1497A mutants were used as parental for purifying cleavage-defective mutants. TseI variants were expressed and purified on a nickel-column. Proteins were washed in 40 mM imidazole wash buffer and eluted in 250 mM imidazole elution buffer. For A&B, the C-terminal His tagged HFH-AAA TseI mutant served as cleavage control for comparison. Cleaved N- and C-terminal fragments are indicated. The D1407A and D1429A mutants exhibited no C-terminal cleavage. Mutations E428A, E429A, and triple EED-AAA but not D435A abolished N-terminal cleavage. EED-AAA refers to triple mutations of E428A, E429A and D435A. **C-D**, Western blotting analysis for expression of wild type TseI and its cleavage-deficient mutants in the Δ*tseI* mutant. Different TseI derivatives with a C-terminal 3V5-tag on pBAD vectors were expressed in the Δ*tseI* mutant upon arabinose induction. The results confirm the D1407A and D1429A not only abolish C-terminal cleavage but also enrich for full length TseI. In addition, E429A but not D435A abolish N-terminal cleavage. The Δ*tseI* mutant carrying an empty pBAD vector serves as control for nonspecific signals of the α-Rhs antibody. Data in (A-D) are representative of at least two replications.

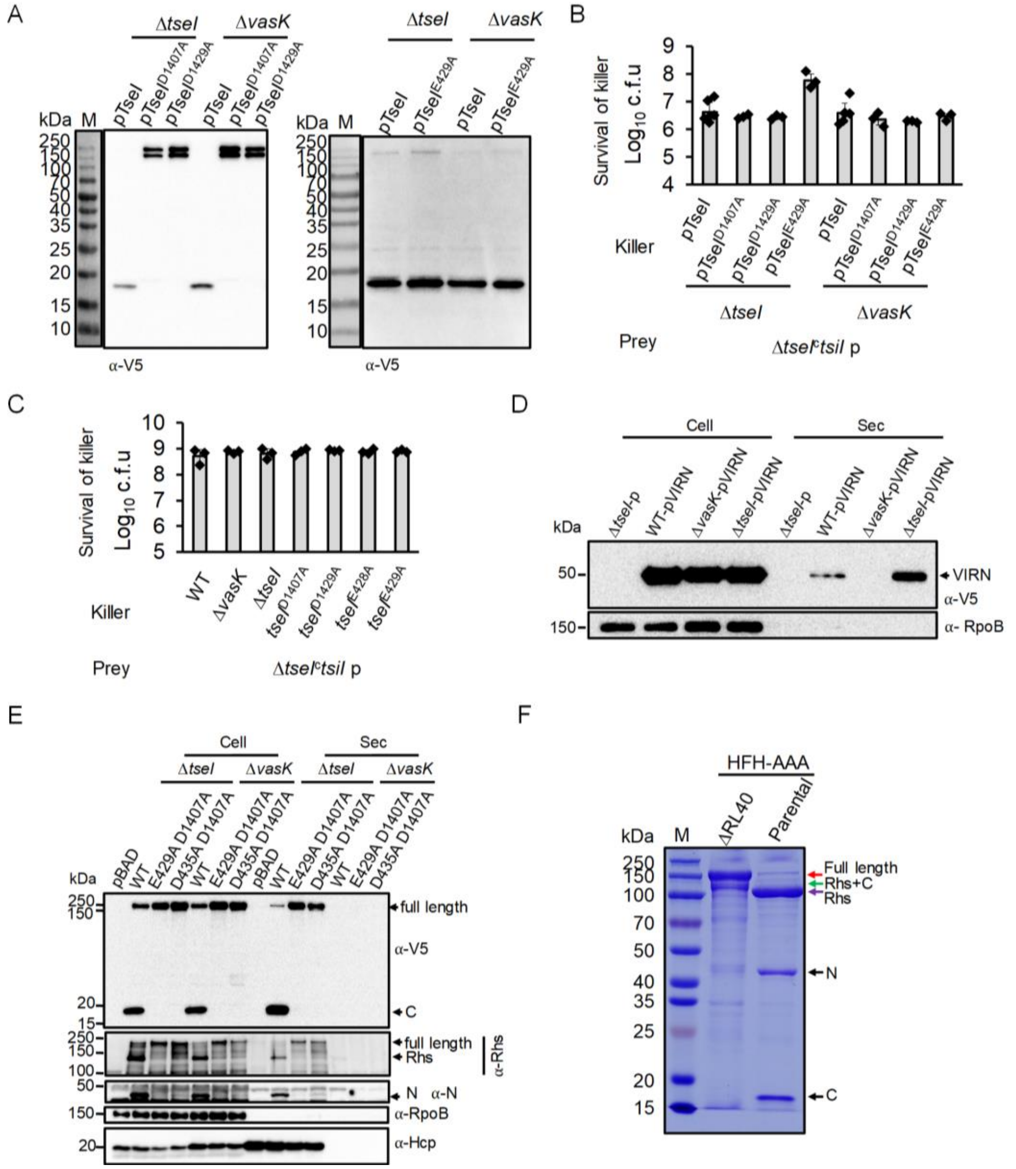

Supplementary Figure 6. Effects of TseI cleavage on secretion and competition. A, Expression of plasmid-borne TseI and its cleavage-defective mutants during competition assays. The cells were collected after co-culturing and TseI expression was detected by western blotting analysis. The survival of the related prey and killer strains is shown in Figure 3A and Supplemental Figure 6B respectively. **B-C,** Survival of killer strains during competition assays for which the survival of the prey is shown in Figure 3A and 3B respectively. Error bars indicate the mean +/- standard deviation of at least three biological replicates (n = 6 for Δ*tseI* and Δ*vasK* carrying pTseI plasmid as killer, and n = 3 for the others). **D,** T6SS-dependent secretion of the VIRN domain of TseI. The VIRN was cloned to a pBAD vector with a C-terminal 3V5 tag and expressed in wild type, the Δ*vasK*, and the Δ*tseI* strains. **E,** Secretion analysis of TseI full-length non-cleaved mutants. T6SS inner tube Hcp serves as a positive control for T6SS delivery. **F,** SDS-PAGE analysis of purified of TseI protease-domain deletion mutant showing the N-terminal cleaved product Rhs+C and the full length. For D&E, proteins with the C-terminal 3V5 tag were expressed on pBAD vectors and induced with 0.01% arabinose. Protein expression and secretion were detected by western blotting analysis and the RNA polymerase subunit RpoB serves as a control for cytosolic expression and cell lysis. Source data are provided as a Source Data file. Data in (A-F) are representative of at least two replications.

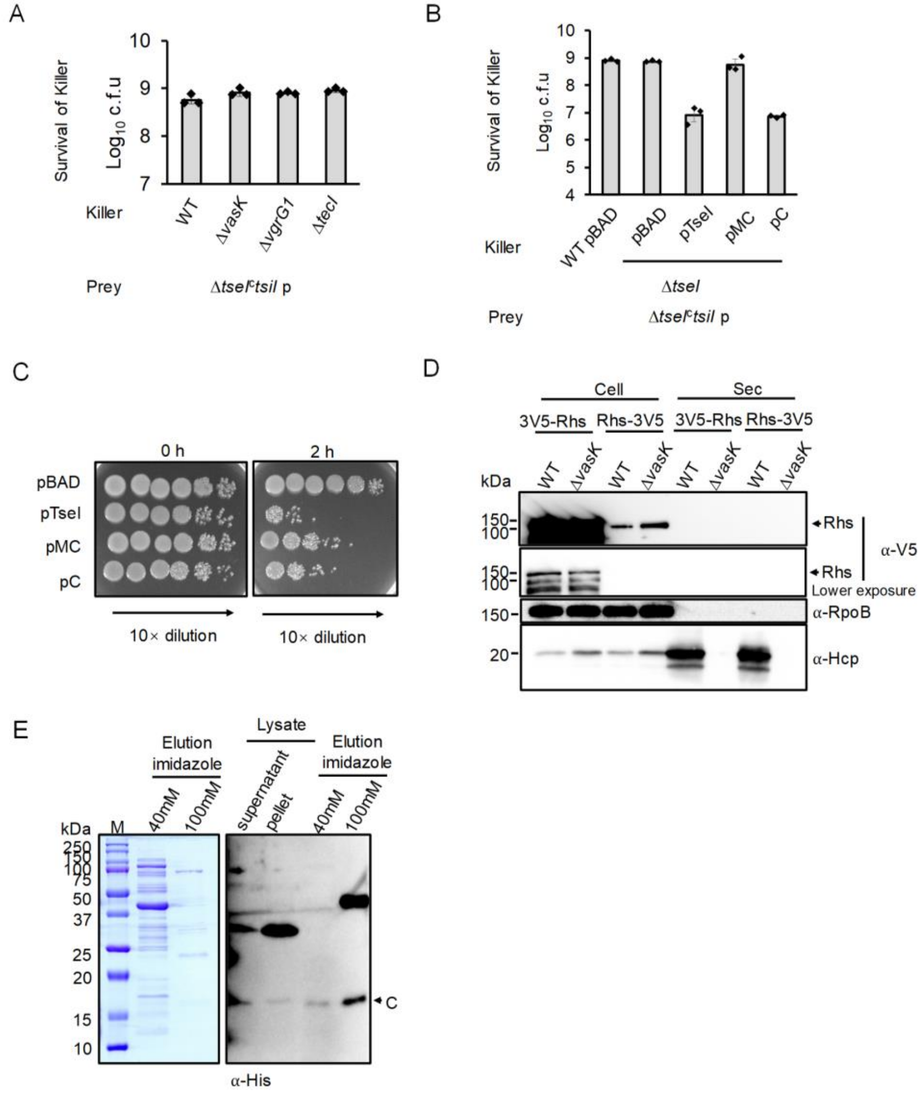

Supplementary Figure 7. Effect of TseI domain deletions on secretion and toxicity. A-B, Survival of killer strains during competition assays for which the survival of the prey cells is shown in Figure 4B and 4I, respectively. Error bars indicate the mean +/- standard deviation of three biological replicates. **C,** Toxicity of expressing plasmid-borne wild type TseI and its truncated mutants in *E. coli*. All proteins were cloned on pBAD vectors. Cells were induced with 0.01% arabinose for 2 h, followed by 10-fold serial dilutions and plating on LB media containing 0.2% glucose to repress expression. The 0 h samples showed the initial quantity of *E. coli* before induction. **D,** T6SS-dependent secretion of the TseI Rhs core. N-terminal or C-terminal 3V5 tagged Rhs on pBAD vectors were expressed in WT or the T6SS-null Δ*vasK* mutant. Detection of the Rhs core was shown in the top two images that differ only in exposure time so that both 3V5-Rhs and Rhs-3V5 samples can be discernible. The RNA polymerase subunit RpoB serves as a control for cytosolic expression and cell lysis, and Hcp is used as a positive control for T6SS secretion. **E,** Protein purification of non-toxic C-terminus of TseI H1497A. N-terminal His-tagged TseI$^{C\ H1497A}$ was expressed on a pET vector with the induction of 1 mM IPTG at 20 ℃ overnight. Cell lysate was loaded on a cobalt-column. Proteins eluted in 40 mM imidazole wash buffer and eluted in 100 mM imidazole elution buffer were subject to SDS-PAGE analysis on the left. The supernatant and the insoluble pellet of centrifuged lysates, and eluted proteins were tested for the C-terminus expression by western blotting analysis on the right using the α-His antibody. Source data are provided as a Source Data file. Data in (A-E) are representative of at least two replications.

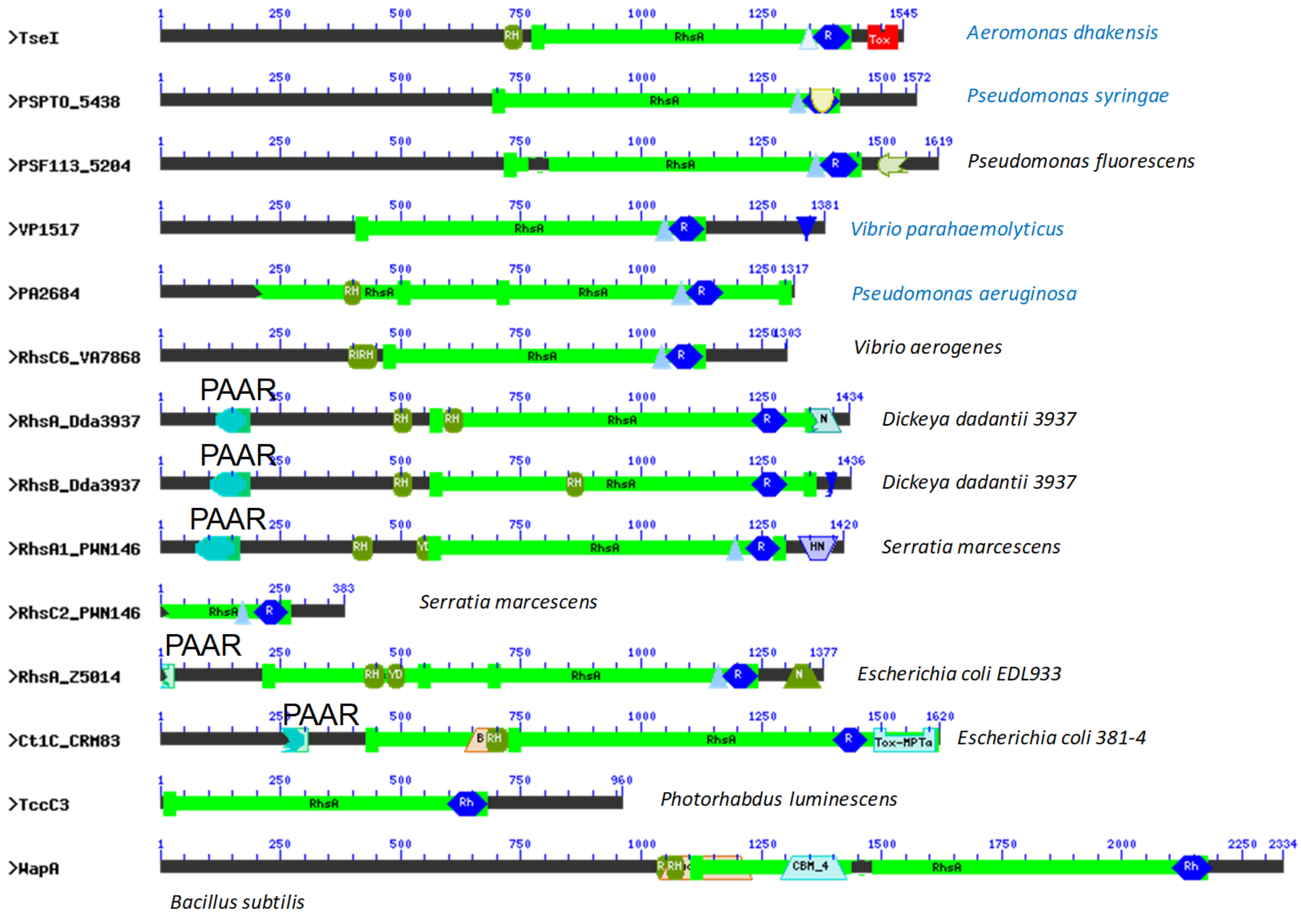

Supplementary Figure 8. Domain structures of TseI homologs. NCBI domain analysis of representative TseI homologs was performed with the Batch Web CD-Search tool using default settings (https://www.ncbi.nlm.nih.gov/Structure/bwrpsb/bwrpsb.cgi). Identified domains and species names are indicated.

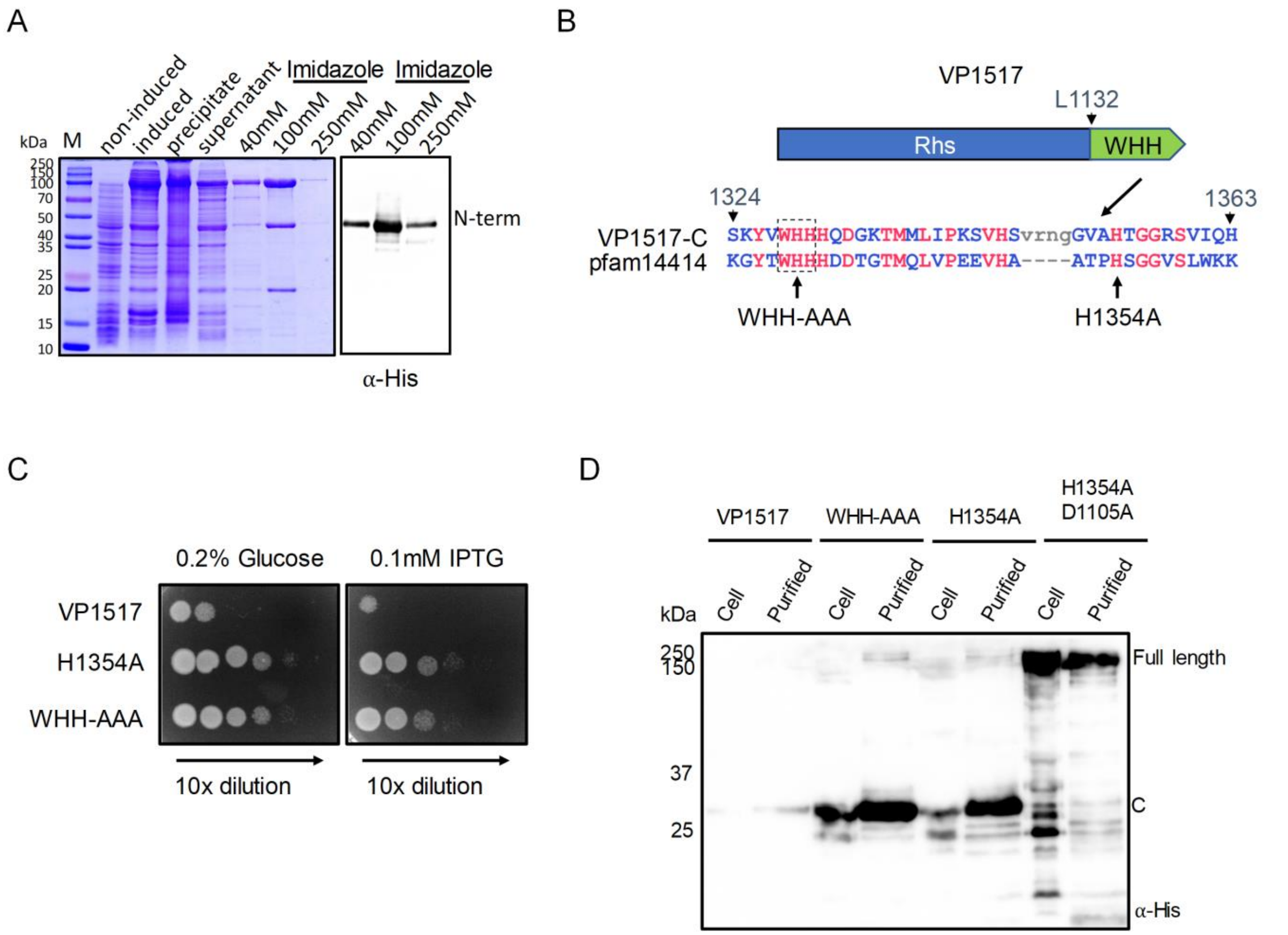

Supplementary Figure 9. Purification and toxicity assay of TseI homologs. A, Protein purification of PSPTO5438. N-terminal His-tagged PSPTO_5438 was expressed with its untagged immunity protein and purified on a nickel-column. The protein was eluted by an increasing gradient of imidazole buffer. To confirm the N-terminal cleaved fragment, western blotting analysis was performed using a commercial α-His antibody. **B,** Predicted catalytic residues of VP1517. The predicted Rhs and WHH domain are indicated. Sequence of the WHH region was aligned with the consensus sequence of pfam14414, a conserved domain family of the predicted HNH/Endonuclease VII toxin containing 4 conserved histidine residues. L1132 is the predicted cleavage site. Two predicted catalytic sites, WHH (1328-1330) and H1354, are indicated. **C,** Toxicity assay of VP1517. VP1517 and its mutants were cloned to pET22b vectors. Survival of *E. coli* BL21(DE3) expressing VP1517, VP1517^{H1354A} or VP1517$^{WHH-AAA}$ was compared on plates containing glucose (repression) or IPTG (induction) with 10-fold serial dilutions. Expression of VP1517 is toxic even in the presence of glucose. **D,** Western blotting analysis confirming the cleavage of VP1517 and its variants. The samples are the same as those used in Figure 5F. Data in (A, C-D) are representative of at least two replications.

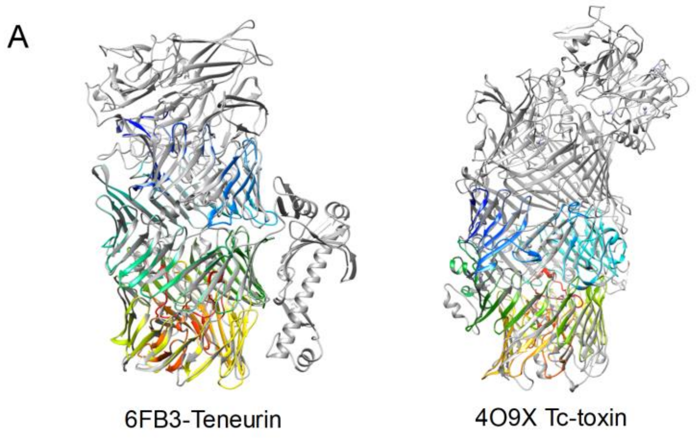

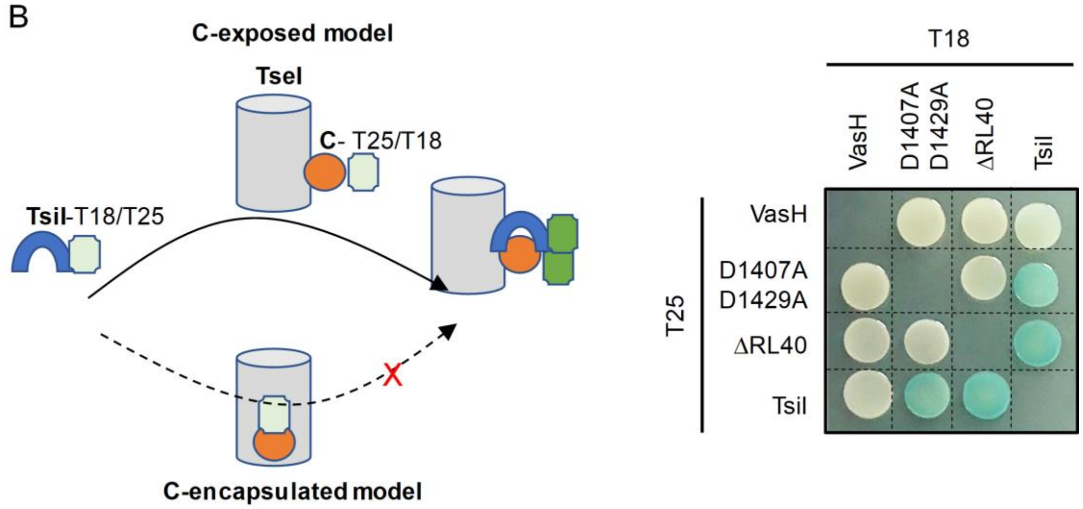

Supplementary Figure 10. Predicted models and two-hybrid assay of TseI and cleavage-defective mutants. A, Modelling of TseI with teneurin and Tc-toxin as the template by Phyre2. The template structures are depicted in grey while the predicted TseI is rainbow-colored with the C-terminus buried inside the shell. N- or C-terminus of TseI was not modeled by Phyre2 due to low similarity. **B,** Bacterial two-hybrid assay showing the interaction of non-cleaved TseI mutants with immunity TsiI. The left is a schematic for testing whether the C-terminus is exposed out of the beta-barrel for interacting with the immunity protein TsiI. TseI^{H1497A} mutants with protease inactivated mutations (D1407A and D1429A) or the protease sequence deletion (ΔRL40) were tested in BTH101 and results are shown on the right. The transcriptional regulator VasH serves as the negative control. Data in B are representative of three replications.

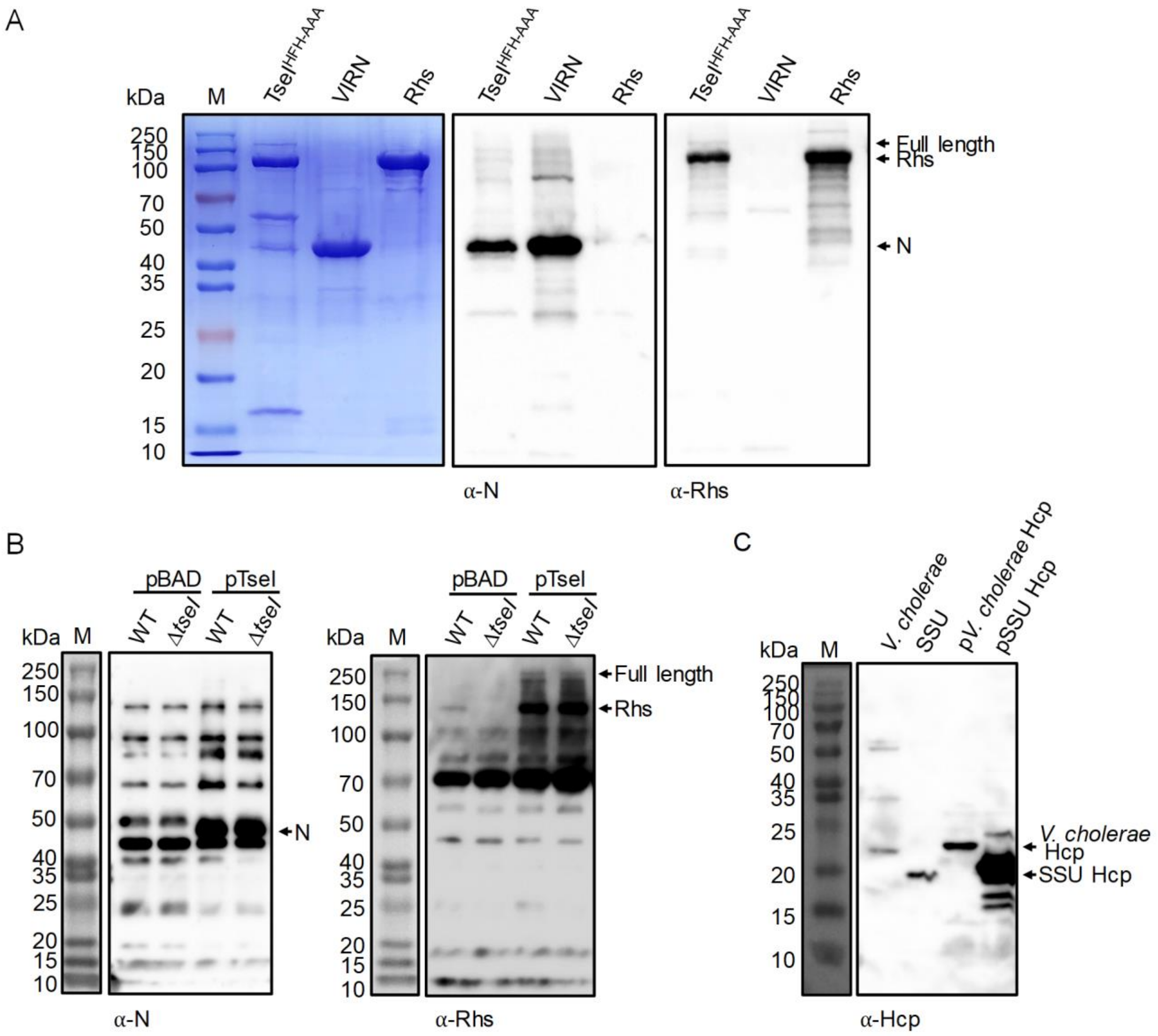

Supplementary Figure 11. Validation of Hcp, TseI N-terminus and Rhs core antibodies. A, Validation of the α-N and α-Rhs using purified proteins**.** N-terminal His tagged TseI$^{HFH\text{-}AAA}$, VIRN and Rhs were purified with a nickel column. As expected, the N-terminus was detected in full-length TseI$^{HFH\text{-}AAA}$ and VIRN samples with α-N antibody while the full-length TseI$^{HFH\text{-}AAA}$ and the Rhs were detected with α-Rhs. **B,** Validation of the α-N and the α-Rhs antibodies using cell lysates**.** Wild type (WT) and the Δ*tseI* mutants transformed with an empty pBAD vector or pBAD expressing full length TseI were tested. The Δ*tseI* mutant carrying pBAD was used as a control for non-specific signals.

Although the signals for both α-N and α-Rhs in WT were low, strong bands corresponding to the predicted molecular weights of N and Rhs fragments were detected in cells expressing TseI. **C,** Detection of Hcp in *Vibrio cholerae* V52 and *A. dhakensis* SSU using the custom α-Hcp antibody. Cell lysates of V52, SSU, and *E. coli* strains expressing plasmid-borne Hcp of V52 and SSU were loaded as indicated. One specific band corresponding to the predicted size of Hcp in SSU was found. This antibody could also detect the *V. cholerae* Hcp homolog. Data in (A-C) are representative of at least two replications.

Supplementary Table 1. Strains

Strain	Genotype	Description	Reference
Aeromonas dhakensis SSU	Parental	Parental strain	[1]
	Δ*vasK*	T6SS null, in-frame deletion of *vasK*	[1]
	Δ*tseI*	In-frame deletion of *tseI*	This study
	Δ$tseI^{c}tsiI$	In-frame deletion of the toxin-coding sequence of *tseI* and *tsiI*	This study
	$tseI^{H1497A}$	Chromosomal mutation of the TseI catalytic residue H1497	This study
	$tseI^{HFH\text{-}AAA}$	Chromosomal mutation of the TseI catalytic residues H1507, F1508 and H1509	This study
	$tseI^{D1407A}$	Chromosomal mutation of the TseI residue D1407	This study
	$tseI^{D1429A}$	Chromosomal mutation of the TseI residue D1429	This study
	$tseI^{E428A}$	Chromosomal mutation of the TseI residue E428	This study
	$tseI^{E429A}$	Chromosomal mutation of the TseI residue E429	This study
	$tseI^{\triangle RL40}$	In-frame deletion of *tseI* internal protease, from arginine 1394 to leucine 1433	This study
	Δ*vgrG1*	In-frame deletion of *vgrG1*	This study
	Δ*tecI*	In-frame deletion of *tecI*	This study
E. coli			
T-Fast	F- *proA+B+ lacIq* Δ*lacZM15 / fhuA2* Δ*(lac-proAB) glnV galK16 galE15 R(zgb-210::Tn10)TetS endA1 thi-1* Δ*(hsdS-mcrB)5*	Strain used for cloning and gene expression	TIANGEN
PIR1	F- Δ*lac169 rpoS(Am) robA1 creC510 hsdR514 endA recA1 uidA(*Δ*MluI)::pir-116*	Strain used for cloning	Invitrogen
SM10 (λ pir)	Km^R , *thi-1, thr, leu, tonA, lacY, supE, recA::RP4-2- Tc::Mu, pir*	Strain used for conjugation	Mekalanos lab
BL21(DE3)	F^- *ompT gal dcm lon* $hsdS_B(r_B^- m_B^-)$ λ(DE3 [*lacI lacUV5-T7p07 ind1 sam7 nin5*]) $[malB^+]_{K\text{-}12}(\lambda^S)$	Strain used for protein expression	Lab stock
BTH101	F', *cya-99, araD139, galE15, galK16, rpsL1 (*Str^R*), hsdR2, mcrA1, mcrB1, relA1*	Host strain used for two-hybrid analysis	[2]

Supplementary References

1. Liang, X. *et al.* Identification of divergent type VI secretion effectors using a conserved chaperone domain. *Proc. Natl. Acad. Sci.* **112**, 9106–9111 (2015).
2. Battesti, A. & Bouveret, E. The bacterial two-hybrid system based on adenylate cyclase reconstitution in *Escherichia coli*. *Methods* **58**, 325–334 (2012).
