# Supplementary material for: Intramolecular chaperone-mediated secretion of an Rhs effector toxin by a type VI secretion system
Source: Nat Commun. 2020 Apr 20;11:1865. doi: 10.1038/s41467-020-15774-z (PMC7170923; doi:10.1038/s41467-020-15774-z)
Supplement: Supplementary file 3 — Description of Additional Supplementary Files [file 41467_2020_15774_MOESM3_ESM.pdf]

Description of Additional Supplementary Files

File Name: Supplementary Data 1
Description: Sequence file of TseI homologs for Figure 2B.

File Name: Supplementary Data 2
Description: Sequence file of TseI homologs for Figure 5.

File Name: Supplementary Data 3
Description: Gene sequence of *hcp1-vgrG1-tecI-tseI-tsiI*.

File Name: Supplementary Data 4
Description: Plasmids used in this study.

File Name: Supplementary Data 5
Description: Primers used in this study.
