# Supplementary material for: Intramolecular chaperone-mediated secretion of an Rhs effector toxin by a type VI secretion system
Source: Nat Commun. 2020 Apr 20;11:1865. doi: 10.1038/s41467-020-15774-z (PMC7170923; doi:10.1038/s41467-020-15774-z)
Supplement: Supplementary file 4 — Supplementary Data 1 [file 41467_2020_15774_MOESM4_ESM.zip › 228713_2_data_set_4503145_q7ndn1.rtf]

>ref|WP_005299972.1MSNAGQAVFNEVIADYRNVLNAYRKDAESFFLGDMLSMDMEQTIKVGDKTITASSSSKKAQSVVTQCPLSGTLRLVHLFESVRFIPIGNTPYKVEAGKLVRGRFVSEAVAKEGTLDGKGIAEVGKLTPGKSYRVTFYPNVKKSDLDTLFTSYVPVQKDLEAWLTKEWNSEHKAAWARYSGTGGGFGSHALAAASGIGKALVGVWDDLKTIYNLLADPVGNAKKLAAFGVDAAAMAKAGASQMEAAMLVLQDEALLYLYAYALVCWVKMLPPDQQTEFGAQVISSVLIDVIIGVVLTGGAGLAARYGAKAASMAKNSDRVMRLVTSLINLSKKHNLAQHAQQAKKVLVAGAAPLSPAKKADLKLVDGNAKTLVEKGTAAKRRFQGHTEIKQVNKTPDASKQAVTPANRPHQPEANTCKNNCPVSMVTGEELLALQDGELPGLLPFVFGRLYRTSAVAQSCGMGSGWTHALAHRLERHGDTLTWWDEESLATELPLPSAARPMVTNLLSEAAVYLGDEADEVILAKAGSPFLHFTWHGDRGRLTGFSDPYGNRLTVRSDRQGRPAWVENEGGLALRLAYEGDHIMALELQRFDGIAWQPQATLQRYHYDDHGHLVAAENGAGECERYRYRDDGVIVERRLAGGAGFFWEWEGEGKAARAIRHWSDVAGFDVTYGWDDDKGEVTVLNADGSQEVYQHDSQARLVRQQDPDGAITEFAYNDKGQKVLARDPLGAETHYHYDEEGMLALEVAPDGSQTAYDYWDGRVRKVVQGEREWRFEHNPQGDLIARRDPLGNETRYGYTPQGKLCSVVHPDSTRTELVWNRLGQLIEEKGVDGGISRWRYDERGRQIVRQDPRGAITRYEWDATDRLSAVHLPGGGVRRYEYNAYGKVTAQWDELGRETRFEYHPGLHLVSRRINPDGSELKYRYDNAKLLLSEIENEHGEQHRIHYHPNGLVARETGFDGRTTAYRYDLNGRLSEKVEFGKQETELVTRYERDLMGRLLKKSLPDGREIAFEYDGHGQLIRVDDGAWPLAFDYDGAGHLVAEHQGWASSYFRYDAMGRLSHWQLPDGNRLGYHHQHGALGGIDLNGAELTRHQVVGGLELRRQQGALTQQYEYDEQGRLSALRLQRGKQVARERRYGYDRTGNLLRIDDSVQGEQHYRYDPLDRLLEVRGELTERFLHDPAGNLLGESSGGQFDGARTQGNRLLFSGDRHFEYDEFGRLARERRGKGQQLVTRYHYDCQHQLVRAELPDGNTACYDYDAFGRRIRKTVRGAKGEQVTEFLWQANNLIAESCYLLGDDMHRTDEQYRSFIYEPGSFKPLAQLEGEGQEAEVFHYQLDHLGTPLALTRVSGVTAWQVRYRAYGNVWREEIAEVATPLRFQGQYFDAETGLHYNRHRYYQPGTGRFITPDPIGLAGGLNNYQYAPNPTGWVDPLGLSNVPGQCPDASPSRRESFRAAKEAAGIPRSAQYKTHKYVYDKEYENRTVYEFDVHGSKKYIVLHDDDKFGRGPHFHGADDSKGSPMLPGKYKQYPGHFPEIKIGFRKGKIKK>ref|WP_042015713.1MSNVGQAVFNEVIADYRNVLSAYRKDAESFFLGDMLSMDMEQTIKVGDKTITASSGSKKAQSVVTQCPLSGTLRLVHLFESVRFIPIGNTPYKVEAGKLVRGRFVSEAVVKEGTLDAKGIAEVGKLTPGKSYRVTFYPNVKKSDLDTLFASYAPVQKDLEAWLTKEWNSEHKAAWARYSGTGGGFGGHALAAASGIGKALVGVWDDLKTIYNLLADPVGNAKKLAELGVDAAAMAKAGASQMEAAMLVLQDEALLYLYAYALVCWVKMLPPDQQTEFGAQVISSVLIDVIIGVILTGGAGLAARYGAKAATMAKNSDRVMRLVTSLVDLSRKHNLTQHAQQAKKVLITGAAPLSPAKKADLKLVDGNANTLVEKGTAAKRRFQGHTEIKQVNKTPDASKQAVTPNNRPHQPEASTCKNNCPVSMVTGEELLALQDGELPGLLPFVFGRLYRTSAVAQSCGMGLGWTHALAHRLERHGDTLTWWDEESLATELPLPSAARPMVTNLLSEAAVYLGDEADEVILAKAGSPFLHFTWHGEIGRLTGFSDPYGNRLTVRSDRQGCPAWVENEAGLALRLAYEGEHIMALELQRFDGIAWQPQATLQRYHYDDHGHLVAAENGAGECERYRYRDDGVIVERRLAGGAGFFWEWEGEGKAARAIRHWSDVAGFDVTYGWDDDKGEVTVLNADGSQEVYQHDSQARLVRQQDPDGAITEFAYNGKGQKVLARDPLGAETHYHYDEEGMLALEVAPDGSQTAYHYWDGRVRKVVQGEREWRFEHNPQGNLIARRDPLGSETRYGYTPQGKLCSVVHPDSTRTELVWNRLGQLIEEKGVDGGISRWRYDERGRQTVRQDPRGAITRYEWDAADRLSAVHLPGGGVRRYEYNAYGKVTAQWDELGRETRFEYHPGLHLVSRRINPDGSELKYRYDNAKLFLSEIENEHGEQHRIHYHPNGLVARETGFDGRTTAYRYDLNGRLSEKVEFGKQGTELVTRYERDPMGRLLKKFLPDGREIAFEYDGHGQLIRVDDGAWPLAFDYDGAGHLVAEHQGWASSHFRYDAMGRLSHWQLPDGNWLGYHYQHGALGGIDLNGAELTRHQVVGGLELRRQQGALTQQYEYDEQGRLSALRLQRGKQVARERRYGYDRTGNLLRIDDSVQGEQHYRYDPLDRLLEVRGELTERFLHDPAGNLLGERSGGQFDGARTQGNRLLLSGDRHFEYDEFGRLALERRGKGQQLVTRYHYDCQHQLVRAELPDGNTACYDYDAFGRRIRKTVKGAKGEQVTEFLWQANNLIAESSYQLGDDLRRTDERYRSFIYEPGSFKPLAQLEGEGQEAEVFHYQLDHLGTPLALTRTSGVTAWQVRYRAYGNVWREEIAEVATPLRFQGQYFDAETGLHYNRHRYYQPGTGRFITPDPIGLAGGLNNYQYAPNPTGWVDPLGLSNVPGQCPDASPSRRESFRAAKEAAGIPRSAQYKTHKYVYDKEYENRTVYEFDVHGSKKYIVLHDDDKFGRGPHFHGADDSKGSPMLPGKYKQYPGHFPEIKFGFRKGKVNK>ref|WP_017411072.1MSNAGQAVFNEVIADYRNVLNAYRKDAESFFLGDMLSMDMEQTIKVGDKTITASSSSKKAQSVVTQCPLSGTLRLVHLFESVRFIPIGNTPYKVEAGKLVRGRFVSEAVAKEGTLDGKGIAEVGKLTPGKSYRVTFYPNVKKSDLDTLFTSYVPVQKDLEAWLTKEWNSEHKAAWARYSGTGGGFGSHALAAASGIGKALVGVWDDLKTIYNLLADPVGNAKKLAAFGVDAAAMAKAGASQMEAAMLVLQDEALLYLYAYALVCWVKMLPPDQQTEFGAQVISSVLIDVIIGVVLTGGAGLAARYGAKAASMAKNSDRVMRLVTSLVNLSKKHNLAQHAQKAKKVLVAGAAPLSPAKKADLKLVDGNAKTLVEKGTAAKRRFQGHTEIKQVNKTPDASKQAVTPAHRPHQPEAKTCKNNCPVSMVTGEELLALQDGELPGLLSFVFGRLYRTSAVSQSCGMGIGWTHALAHRLERHGDTLTWWDEESLATELPLPSVARPMVTNLLSEAAVYLGDETDEVIVAKAGSPFLHFTWHGDSGRLTGFSDPYGNRLTVRADRQGRPAWVENEGGLALRLVYEGDHIMALELQRFDSIAWQPQATLQRYHYDDHGHLVAAENGVGECEQYRYRDDGVIVERRLAGGAAFFWEWEGEGKAARAIRHWSDVAGFDVTYDWDDDKGEVTVINADGSHEVYQHDSQARLVRQQDPDGAITESAYNAKGQKVLARDPLGGETHYHYDEEGLLVLEVAPDGTQTAYQYWDGRVRKIVQGEREWRFEHNLQGDLITRRDPLGSETRYDYTPQGKLCAVVHPDGSRTELVWNRLGQLIEEKGADGGISRWRYDERGRQIVRQDPRGAITRYEWDAADRLSTVHLPGGGIRRYEYNAYGKVTAQWDEQGRETRFEYHPGLHLVSRRINPDGSELKYRYDNAKLFLSEIENEHGEQHRIHYHPNGLVARETGFDGRTTVYRYDLNGRLSEKVEFGKQETELVTRYERDPMGRLLTKSLPDGREIAFEYDSHGQLIRVDDGAWPMAFDYDSAGHLLAEHQGWASSHFQYDAMGRLSHWQLPDGNQLGYHYQHGALGGIDLNGAELTRHQVVGGLELRRQQGALTQQYEYDEQGRLTALRLQRGKQVSRERRYGYDRTGNLLRIDDSVQGEVHYRYDPLDRLLEVRGELTERFLHDPAGNLLGESSGGQFDGARTQGNRLLLSGDRHFEYDEFGRLAVERRGKGQQLVTRYHYDCQHQLVRAELPDGSNACYDYDAFGRRIRKTVSGAKGGQVTEFLWQANNLIAESSYQLGDDQRRTDEQYRSFIYEPGSFKPLAQLEGEGQKAEVFHYQLDHLGTPLALTRTSGATAWQVRYRAYGNVWREEIAEVATPLRFQGQYFDAETGLHYNRHRYYQPDTGRFITPDPIGLAGGLNNYQYAPNPTGWVDPLGLSNVPGQCPDASPSRRESFRAAKEAAGIPRSAQYKTHKYVYDKEYENRTVYEFDAHGSKKYIVLHDDDKFGRGPHFHGADDSKGSPMLPGKYKQYPGHFPEIKLGFRKGKVKK>ref|WP_042048877.1MSNAGQAVFTEVIADYRNALKEYRKDAESFFLGDMLSMDMEQTIKVGDKTITASSSSKKTQSVVTQCPLSGTLRLVHLFESVRFIPIGNTPYKVEAGKLVRGRFVSEAVAKEGTLDDQGIAEVGQLTPGKSYRVTFYPDVQKGDLDTLFASYVPVQKDLEAWLTKEWNSEHKAAWARYSGSGGGFGSHTMAAASGIGKALVGVWDDLNTIYNLLADPVGNAKKLAEFGVDAVAMAKAGASQMEAAMLVLQDEALLYLYAYALVCWVKMLPPDQQTEFGAQVISSVLIDVIIGVVLTGGVGLAARYGAKAATMAKNSDRVMRLVTSLVDLSRKHNLAQHAQKAKKVLITGAAPLSPAKKADLKLVDGNARTLVEKGTAAKRRFQGHTEIKQVTKTPDASKQATTPNNHPHQSEAKTCKNNCPVSMVTGEELLALQDGELPGLLPFVFGRLYRTSAVAQSCGMGLGWTHALAHRLERHGDTLTWWDEESLATELPLPSAAKPMVTNRLAEAAVYLGDEPDEVIVAKAGSPFLHFTWHGNRGRLTGFSDPYGNRLTVRSDRQGRPARVENEGGLALRLVYEGDHIMALELQRFDGIAWQPQATLQRYHYDDHGHLVAAENGAGECERYRYRDDGVIVERHLAGGAGFFWEWEGEGKAARAIRHWSDVAGFDVTYGWDDDKGEVTVINADGSQEVYQHDSQARLVRQQDPDGAITEFAYNDKGQKVLARDPLGAETHYHYDEEGLLTLKVAPDGSQTDYHYWDGRVRKIVQGEREWRFDHNPQGDLIARRDPLGNETRYGYTPQGKLCAVVHPDGTRTELVWNRLGQLIEEKGVDGGISRWRYDERGRQIVRQDPRGAITRYEWDAADRLNAVHLPGGGVRRYEYNAYGKVTAQWDEQGRETRFDYHPGLHLVSRRINPDGSELKYRYDNAKLFLSEIENEHGEQHRIHYYPNGLVSQETGFDGRTTAYRYDLNGRLSEKVEFGKQETELVTRYERDPMGRLLKKSLPDGREIAFEYDAHGQLIRVDDGAWPLAFDYDSAGHLLAEHQGWASSHFQYDAMGRLSHWQLPDGNRLGYHYQHGVLGGIDLNGAELTRHQVVGGLELRRQQGTLTQQYEYDEQGRLTALRLQRGKLVARERRYGYDRTGNLLRIDDSVQGEQHYRYDPLDRLLEVRGELTERFLHDPAGNLLSQTQGDKFDAARTQGNRLLLSGDRHFEYDEFGRLALERRGKGQQLVTRYHYDCQHQLIRAELPDGSTARYDYDAFGRRIRKTVKGATGEQVTEFLWQANNLIAESSYLLGDDKRRTDEQYRTFIYEPGSFKPLAQLEGEGQKAEVFHYQLDHLGTPLALTRTSGATAWQVRYRAYGNVWREEIAEVTTPLRFQGQYFDAETGLHYNRHRYYQPGTGRFITPDPIGLAGGLNNYQYAPNPIGWIDPLGLSNLPSQCPDTLPRARISAEEQDVFKQFEQHHAGQFKDDHELVSAFEGLRDNESPWPVGFTPKSRTIEPGERFNMALGPGQPITSPGGFGSMDSIHSADFVWNELAVKQSWKPAGIDRVVTYEVTHPFEVLEGPVGPQVEKLYDGSYKYLTGGANQINLRLPRDPVEKMNYLKVVDVKDIK>ref|WP_042869798.1MSNAGQAVFTQVIADYRNALTEYRKDAESFFLGDMLGMDMEQTIKVGDKTIKASSSSKKAQSVVTECPQSGTLRLVHLFESVRFIPIGNTPYKVEAGKMVRGRFVSEAVADEGTLDAKGIAEVGKLAPGQSYQVTFYPKVKKSDLDALFASYVPVQQDLEAWLTKEWNSEHKAAWGRYSATGAGFGSHALAAASGVGKALVGVWDDLKTIYNLLADPVGNAKKLAEFGVDAAAMAKAGASQMEAAMLVLQDEALLYLYAYALVCWVKMLPPDQQTEFGSQVISSVLIDVIIGVILTGGAGLAARYGAKAASAAKNSDRVMRLVTSLVNLSKKHNLAQHAQTAKKVLIAGSAKLSPAKKADLKLVDGSAKTLVEKGTAAKRKFQPHSEIKQLKKTLDASKTATTPVNKPHQPEAKTCKNNCPVSMVTGEELLALEDAHLPGMLPFTFGRLYRTSAVERSCGMGAGWSHALAHRLERHGDRLTWWDQESLAIELPMPSATRPMITNQLSEAAVYLGDEPDEVIVAKAGSPFLHFTWHGKTGRLTAMSDLYGNRLTIRADEQGRPCWIENEGGLALRIVYQKAYLAAVELQHFDGINWQPEATLQRYDYDDAGHLVVAENGAGECERYRYRPDGVILERRLAGGAGFFWEWEREGKLARAVRHWSDVARFDVSYTWDDDKGEVTVSNADGSQEVYQHDSNARLIRQQDPDGAVSEFVYNDKGQKVLARDALGGETRYHYDEAGLLDREIAPDGSQTAYHYWDGRVRKVVQGDREWRFERNEQGDVIARRDPLGRETRYSYNAQGKLNTVVQPDGSRIELGWNRLGQLIEEKGANGGVTRWRYDERGRQIVRRDPRGAITRYEWNAADRLQAVYLPGGGSRRFEYNAYGKVTAEWDELGRETRYEYHPGLHLVSRRINPDGSELKYRYDNAKLFLSEIENEHGEQHRIHYFPNGLVARETGFDGRTTAYRYDLNGHLSEKVEFGKQETELVTRYERDPMGRLLKKTLPDGREIQFSYDQYGQLTQVDDGAWPLTFEYDTAGNLLAEHQGWASSYFKHDAMGRLSHWQLPDGNKLAYHYLNGELSGIDLNGAELTRHQRVGGLEMRRSQGALTQQYEYDEQGRLTALRLQRGKQVARERRYGYDHTGNLLQINDSVQGEQHYRYDPLDRLLEVRGELTERFLHDPAGNLLSQTSGGQFDGARTQGNRLLLSGDRHFEYDEFGRLAIERRGKGQSLVTRYHYDCQHQLVRAELPDGTTANYDYDAFGRRIRKTVRGTKSEHVTEFLWQANNLIAESSYQLGDDKRRTDEQYRSFIYEPGSFKPLVQLEGEGTDTEVFHYQLDHLGTPLALTRDNGATAWQVRYRAYGNVWREEIADVATPLRFQGQYFDAETGLHYNRHRYYQPETGRFITPDPIGLAGGLNNYQYAPNPTGWVDPLGLTNIPGQCPPGPNKKTSYEAETRREAFRQAKRDAGIPMTQQPVNITRPELLDGDGRVIMGTNNQPITTKQYEFINTKGENIFIQEHSLGHAKATPGHGLEPHFNIRPSDNLNTGSVLGTHGHYNFGVKK>ref|WP_103470572.1MSNAGQAVFTQVIADYCNALTEYRKDAESFFLGDMLGMDMEQTIKVGDKTIKASSSSKKAQSVITQCPLSGTLRLVHLFESVRFIPIGNTPYKVEAGKMVRGRFVSEAVANEGTLDAKGIAEVGKLTPGKSYRVTFYPKVKKSDLDALFASYVPVQKDLEAWLTKEWNSEHKAAWGRYSATGAGFGSHALAAASGVGKALVGVWDDLKTIYSLLADPVGNAKKLAEFGVDAAAMAKAGASQMEAAMLVLQDEALLYLYAYALVCWIRMLPPDQQTEFGSQVISSVLIDVIIGVILTGGAGLAARYGAKAASAAKNSDRVMRLVTSLVNLSKKHNLAQHAQTAKKVLIAGSAKLSPAKKADLKLVDGSAKTLVEKGTAAKRKFQPHSELKQLKKTPDASKTATTSANKPHQPEAKTCKNNCPVSMVTGEELLALEDAHLPGMLPFTFGRLYRTSAVERSCGMGAGWSHALAHRLERHGDSLTWWDQESLAIELPMPSAARPMITNQLSEAAVYLGDEPDEVIVAKAGSPFLHFTWHGKTGRLTAMSDLYGNRLTIRSDEQGRPCWIENEGGLALRIVYQKAYLAAVELQHFDGINWQPEATLQRYYYDDAGHLVVAENGAGECERYRYRPDGVILERRLAGGAGFFWEWEREGKLARAVRHWSDVARFDVSYGWDDDKGEVTVSNADGSQEVYQHDSNARLIRQQDPDGAVSEFVYNDKGQKVLARDALGGETRYHYDEAGLLEREIAPDGSQTAYHYWDGRVRKVVQGDREWRFERNEQGDVIARRDPLGRETRYSYNAQGKLNTVVQPDGSRIELGWNRLGQLIEEKGANGGVTRWRYDERGRQIVRRDPRGTITRYEWNAADRLQAVYLPGGGSRRFEYNAYGKVTAEWDELGRETRYEYHPGLHLVSRRINPDGSELTYRYDNAKLFLSEIENEHGEQHRIHYSPNGLVARETGFDGRTTAYRYDLNGHLSEKVEFGKQETELVTRYERDPMGRLLKKTLPDGREIQFSYDQYGQLTQVDDGAWPLTFEYDTAGNLLAEHQGWASSYFKHDAMGRLSHWQLPDGNKLAYHYQHGELSGIDLNGAELTRHQRVGGLEMRRSQGALTQQYEYDEQGRLTALRLQRGKQVARERRYGYDHTGNLLQINDSVQGEQHYRYDPLDRLLEVRGELTERFLHDPAGNLLSQTSGGQFDGARTQGNRLLLSGDRHFEYDEFGRLAIERRGKGQSLVTRYHYDCQHQLVRAELPDGTTANYDYDAFGRRIRKTVRGTKGEHVTEFLWQANNLIAESGSDGGYRSFIYEPGSFKPLVQLEGEGQDAEVFHYQLDHLGTPLALTRDNGATAWQVRYRAYGNVWREEIAEVATPLRFQGQYFDVETGLHYNRHRYYQPETGRFITPDPIGLAGGLNNYRYAPNPIGWVDPLGLMNKPGQCPDSEKVVEKKMEGPRPPVVHLDPQDIRFSQNSVSFNKTERGSGKPYTYDDLVASMRENGWQGDPIDVVKMPDGGLTSMDNTRVRAAREAGVKVQARVNDFERPLTEAERERFTKADTAPDTWGEAILLRINSQKPKVFAKSNPHGANVEPKLSGKK>ref|WP_058051647.1MSNAGQAVFTQVIADYRNALTEYRKDAESFFLGDMLGMDMEQTIKVGDKTIKASSSSKKVQSVVTQCPLSGTLRLVHLFESVRFIPIGNTPYKVEAGKMVRGRFVSEAVAKEGTLDAKGIAEVGKLTPGKSYRVTFYPKVKKSDLDALFASYLPVQQDLEAWLTKEWNSEHKAAWGRYNATGAGVGSHALAAASGVGKALVGVWDDLKTIYNLLADPVGNAKKLAEFGVDAAAMAKAGASQMEAAMLVLQDEALLYLYAYALVCWVKMLPPDQQTEFGSQVISSVLIDVIIGVILTGGAGLAARYGAKAASAAKNSDRVMRLVTSLVNLSKKHNLAQHAQTAKKVLIAGSAKLSPAKKADLKLVDGSTKTLVEKGTAAKRKFQPHSELKQLKKTPDASKTATTPVNKPHQPEAKTCKNNCPVSMVTGEELLALEDAHLPGMLPFTFGRLYRTSAVERSCGMGVGWSHALAHRLERHGDSLTWWDQESLAIELPMPSATRPMITNQLSEAAVYLGDESDEVIVAKAGSPFLHFTWHGKTGRLTAMSDLYGNRLTIRADEQGRPCWIENEGGLALRIVYQKAYLTAVELQHFDGINWQPEATLQRYYYDDAGHLVVAENGAGECERYRYRPDGVILERRLAGGAGFFWEWEREGKLARAVRHWSDVARFDVSYTWDDDKGEVTVSNADGSQEVYQHDSNARLIRQQDPDGAVSEFVYNDKGQKVLARDALGGETRYHYDEAGLLEREIAPDGSQTAYHYWDGRVRKVVQGDREWRFERNEQGDVIARRDPLGRETRYSYNAQGKLSTVVQPDGSRIELGWNRLGQLIEEKGANGGVTRWRYDERGRQIVRRDPRGAVTRYEWNAADRLQAVHLPGGGSRRFEYNAYGKVTAEWDELGRETRYEYHPGLHLVSRRINPDGSELKYRYDNAKLFLSEIENEHGEQHRIHYFPNGLVARETGFDGRTTAYRYDLNGQLSEKVEFGKQETELVTRYERDSIGRLLTKTLPDGREIQFSYDQYGQLTLVDDGAWPLTFEYDTAGNLLAEHQGWASSYFKHDAMGRLAHWQLPDGNKLAYHYLHGELSGIDLNGAELTRHQMVSGLEMRRSQGALTQQYEYDEQGRLTAQRSQRGKQVARERRYGYDRTGNLQQINDSVQGEQHYRYDPLDRLLEVRGELTERFLHDPAGNLLSQTLGSQFDGARTQGNRLLLSGDRHFEYDEFGRLAIERRGKGQSLVTRYHYDCLHQLVRAELPDGTTARYDYDAFGRRIRKTVSGAKGELVTEFLWQANNLIAENSYQLGDDKRRTDEQYRSFIYEPGSFKPLVQLEGEGTDTEVFHYQLDHLGTPLALTRDNGVTAWQVRYRAYGNVWREEIAEVATPLRFQGQYFDAETGLHYNRHRYYQPETGRFITSDPIGLAGGLNNYQYAPNPIGWVDPLGLMNQPGHCPGSEEIVETRMEGPRPPVVHLDPQDIRFSQNSVSFNKTERGSGKPYTYDDLVASMRENGWQGDPIDVVKMPDGGLTSMDNTRVRAAREAGVKVQARVNDFERPLTDEETRRFKTTSVPDTWGEAIVLRIEKQKPKSFSKNNQFGSDADPKLSGKK>ref|WP_058058709.1MSNAGQAVFTQVIADYRNALTEYRKDAESFFLGDMLGMDMEQTIKVGDKTIKASSSSKKAQSVVTQCPLSGTLRLVHLFESVRFIPIGNTPYLVESGKMEKGKFVPAKEERKGSLDAKGVAEIGSLKPGESYRVTFYPNVKKSDFDGLFRSYQVVQADLAAWLEREWASTHQPAWVNYQRNGGGGVAVAAGVLRGIGRALASVWDDLTGLYDLLADPMGNAEKLLKFGVNAADVAQAGAEKIESAMLVLQDEALIYLYVNALVSWLKMLPPDELVEFGTQAVVTVLFDVLVGIVLTGGAGIAVRYSAKVATTMTRAAKQQARMAKLAATLIAMSKKHNLVAHIDVAKPVLVTGGAPLNPLKKADLQLIDRGASTLVEKATAESRRHKSRTTIKQVGSTPDTSRHSTNAAGHASQTEAKTCKNNCPVSMVTGEELLALEDAHLPGMLPFTFGRLYRTSAVERSCGMGAGWSHALAHRLERHGDSLTWWDQESLAIELPMPSAARPMITNQLSEAAVYLGDEPDEVIVAKAGSPFLHFTWHGKTGRLTAMSDLYGNRLTIRADEQGRPCWIENEGGLALRIVYQKAYLTAVELQHFDGINWQPEATLQRYYYDDAGHLVVAENGAGECERYRYRSDGVILERRLAGGAGFFWEWEREGKLARAVRHWSDVARFDVSYTWDDDKGEVTVSNADGSQEVYQHDSNARLIRQQDPDGAVSEFVYNDKGQKVLARDALGGETRYHYDEAGLLECEIAPDGSQTTYHYWDGRVRKVVQGEREWRFERNEQGDVIARRDPLGRETRYSYNTQGKLSTVVQPDGSRIELGWNRLGQLIEEKGANGGVTRWRYDERGRQIVRRDPRGAITRYEWNAADRLQAVYLPGGGSRRFEYNAYGKVTAEWDELGRETRYEYHPGLHLVSRRINPDGSELKYRYDNAKLFLSEIENEHGEQHRIHYFPNGLVARETGFDGRTTAYRYDLNGHLSEKAEFGKQETELVTRYERDSMGRLLKKTLPDGREIQFSYDQYGQLTQVDDSAWPLTFEYDAAGNLLAEHQGWASSYFKHDAMGRLAHWQLPDGNKLAYHYLHGELSGIDLNGAELTRHQMVGGLEMRRSQGALTQQYEYDEQGRLTALRLQRGKQVARERRYGYDRTGNLLQINDSVQGEQHYRYDPLDRLLEVRGELTERFLHDPAGNLLSQTLGSQFDGARTQGNRLLFSGDRHFEYDEFGRLAIERRGKGQSLVTRYHYDCQHQLIRAELPDGTTARYDYDAFGRRIRKTVSGAKGELVTEFLWQANNLIAESSYQLGNDKHRTDEQYRSFIYEPGSFKPLVQLEGEGQDAEVFHYQLDHLGTPLALTRDNGATAWQVRYRAYGNVWREEITEVATPLRFQGQYFDAETGLHYNRHRYYQPETGRFITPDPIGLAGGLNNYQYAPNPTGWVDPLGLTNIPGQCPPGPNKKTSYEAETRREAFRQAKRDAGIPMTQQPVNITKPELLDGNGRVIMGTNNQPITTRQYEFINAKGESVFIQEHSLGHAKATPRHGLEPHFNIRPSDNLNTGSVLGTHGHYNFGVKK>ref|WP_082041607.1MSNAGQAVFNQVIADYRNALKEYRKDAESFFLGDLLSMDMEQTIKVGDKKVSVSSNSKKAQSVVTQCPLSGTLRLVHLFESVRFIPIGNTPYKVEAGKMDRGRFVSESVAKEGTLDAKGIAEVGKLVPGKSYRVTFYPNVKKADLDGLFASYAPVQQDLIDWLTKEWGSEHKAAWARYTMNGGGFGAHSLAAVSGIGKALVGVWDDLKTIYDLLADPVGNAKKLAEFGVDAAAMAAAGASQMEAAMLVLQDEALLYLYAYALVSWVKLLPPDEQTEFGTQVIATVLIDVIVGVVLTGGAGLAVRYSVKAAGAAKKQARMVVLVTSLISMSKKHNLVQHAQSAKKVLVAGVAPLNPAKKADLKLVDSGVMTEVVQGTAAKRRFEAHTEIKQVNNTPDASKQGRTPSDQAHQPEAQTCTNGCPVSMVTGEELLALEDAQLPGMLPFTFGRLYRTSAVALDRGMGAGWSHSLAHRLERHGETLTWWDDESLAIELPLPSGRMPMATNRLSSAAVYLGDTDDEVILAKAGSPFLHFFWHGNEGCLTALSDAYGNRLAVRADEKGRPCWLENEGGLALRLVYQGNRLCALELQQFDGLHWQPQSTLQRYHYDEAGRLVAAENGVGEQERYRYRPDGVILERRLAGGAGFFWEWEGEGNKARAIRHWSDVARFDVSYGWDDDKGEVTVQSVDGSLEVYRHDSNARLVRQQDPDGAITEFDYNDQGQKVLACDALGGETRYHYDDAGLLSLEIAPDGSETAYSYWDGRVRKVVQGEREWRFERNEQGDVIARVDPLGRQTRYGYTAQGKLSWVAQPDGSRIELLWNRLGQLIEEKGTDGGLTRWRYDERGRQILRRDPRGAITRYEWDGADRLRAVHLPGGGSRRFEYNPYDKVTAEWDELGRETRYEYHDRLHLLSRRINPDGSELKYRYDNAKLFLSEIENEHGEQHRIQYYPNGLVAQETGFDGRTIAYRYDLNGHLCEKVEYGKEEAELVTRYERDAMGRLLSKQLPDGREIQFAYDTYGQLIRVDDGVWPLAFEYDKGGNLTAEHQGWASSYFKHGVMGQLAQWRLPDGNHLDYRYQDGVLSGIDLNGSELTRHQVAAGLEMRRRQGALTQQYEYDEQGRLTALRLHRGQQVSRERRYGYDRSGNLLRVNDSAQVAAGNPQGEQHYRYDPLDRLLEVCGELTERFMHDPAGNLLSQTQGNQFEPARTQGNRLLFSGDRHFEYDEFGRLATERRGKAQRLFTRYEYDCQHQLIVAELPDGSRARYDYDAFGRRIRKTVTNGADEQVTEFLWQANNLIAESRSDGAYRSFIYEPGSFKPLVQLEGEGKQAEVYHYQLDHLGTPLALTHHDGHSAWQVRYRAYGNVWKQELAEVETPLRFQGQYYDTETGLHYNRHRYYQPDTGRFITPDPIGLAGGLNNYQYAPNPTGWVDPLGLMLKATNCLKHAFKFTVDKHGNMPKPRPGLNSHHGVMSAWMKHNFPEYDASQAPAVLMPEANHRATFGVYNTWRADMRKSMGGEFDWGKVTEDEVKSLSGKMFDAAEVPADIRKEYWDWYLRMNKVLME>ref|WP_041997575.1MSNAGQQLFSQVIKDYQQSLAAYRKDAESYWLGETFGMNMEQTIKVGDQKITASTSSSEVESVVTQCPLSGTLRLVHMFESVRFIPIGNTPYKIEPVKRVKGRYVNDGPIKSGTLDAKGIAEVVQLEPGKSYRVSFYPDLHKSDLETLFASYAPVQADLVAWLTQEWNASLSQDWDRYTASGAGLGSHAGAAVSGIGKALVSVWDDLKTIYDLLAHPQENLEKLAEFGIDAAAMAATGASQIEAGLLVLQDEALLYLYANALISWLRLLPPDQQTEFGAQVVATVLFDVLVGVVLTGGAGLAARYGVKAIGAAKQSERITRLVGALVELSKKHNLAQHAQGVKKVLLAGENKLNPGKKANLDLVDGGTTTKLEQGTAVTRDHHATTRIEQADNTPDSSSPATTGSGHASQRENETCLNNCPVSMVTGEELLALDDGQLPGMLPFTFTRLYRTSAVAMDSGMGAGWSHSLSHRLARHGDTLTWWDSEAKAITLPHPTRQMPMATNRLAKAAVYLGDDADEVIVAQADSPFLHFRWQGNTGRLTALSDTYGNRLEVRHDDQGRPSRVENQAGLALRLRHDNRRITAIELQRFDGIQWQSLTTLQRYHYNSQGQLGAAENGAGERECYEYRADGVILARRLAGGASFFWEWQGEGHLARAIRHWSDVAGFDVRYDWDDEKGQVTVNNADGSQEVYQHDDNARLIRQQDPDGAVSEFVYNDKGQKVLARDGLGAETRYQYDDRGQLSLEIAPDGSQTVYHYWDGKLRKLEQGDREWRFEHNEQGDPVRRLDPLGQQTRYGYTAQGKLAWVEQADGSRIQLGWNRLGQLIEEKAADGTLTRWRYDERGRQILRQDARGAITRYEWDDADRLRQITLPGGGTRRFQYNPYGKVTHEWDEQGRETRYEYHDRLHLISRRINPDGSELKYRYDNAKRFLSQIENEHGEQHRIHYHPNGLVAEETGFDGRTSRYAYDLNGRLTEKTEVGRSGTELVTLYRRDVMGRLTSKVLPDGQEISFHYDAHGQLTSVEDGHWPLAFSYDKAGRLTAEHQGWASSYFKHDALGQLSQWRLPDGNRLDYQHHNGTLTGIDLNGSELTRHQIVAGLELRRSQGALSQQYEYDEQGRLTALRLQAGQQITHQRQYGYDRSGNLLHIRDSQQGESRYDYDPLDRLLEVRGDLSERFLHDPAGNLLSQTQGSAFTSARTQGNRLLFSGDRHFDYDEFGRLTTERCGKGQALVTRYEYDCQHRLIQAELPDGSTARYHYDAFGRRIRKTLARQGQDPLITEFLWQANNLIAESCSDDTYRSFIYEPGSFRPLVQLEGEGEAAEPYHYQLDQIGTPLALTSHSGQTAWRVRYRAYGNVWKQEIAEVESPLRFQGQYHDAETGLHYNRHRYYQPDTGRFITPDPIGLLGGLNNYRYGTNPARWVDPLGLSNQDVLCPGGAVKAANTAGATKPADMPITQDKYDEIINLERGNRPENPKSYLPSEYVDAHEKLFEEQGGGFFQFANWLNPKSPHKTLPKGKFVGLSGDMDVVFDNAKKIQDPMSRARYLDKELALGLNEVQLQEISQSEIYYIRIKPGDKRFNYKMPTGNEPGALSGEWVPGGKTKGGAKEAALHGAEKIKHDGYVDKLISYFDDATRLQ>ref|WP_083603141.1MIPRLGSLSFIGLGAESPDASKVISEFKSCLGTYKELAESFWTGWALDADQTFKIGDELSVSKGKDYTSPVTNYATCPLNGEFTLVHAFEAARFVPIGNTPVRLEPVTRAWNGDEVTGPVINDEIGPSGIKVISGCKRGQLYRITFFPNVTKAQVEALYSSYQGVIQKLAAWLQSEWTNQFQPLWSTYQSANSGQRLKLQLEAALEGFEHALLSLWDDIKSLFQLIAHPYENAKKLAKYLSPDELKKLYTASKESLSTALLIASDEPLMFIYVAAIVSWVKLLPPQTCVEVLAQFTGELLINIVVGIILSGGAGLAIRVGIKALETLKSGQATALISKLADTLINASQSHALSVHADTLKPLAAHGEISEANTARKINAKVAPASKRPTPADTPSQTIENASTTSRNKTRRQTRLSREEKVDDVSKPAKDPNDKPSQECAKTCTNGCPVSMVTGEELLTLTDGELDGVLAFPWSRLYRTSAIEHNVGLGYGWSHSLAHRLEIQGDTVIWTDHENRRTEFPLPSQQRPAITNRLSAAAIYLGEDLSELVLAQAGAKPHFYHFRRIGQSARLISISDTYNNVLRISRDARDRIVRLTNTAGRALRFTYEHAHIVAVDYQVYRPADNEEASWYTIQTLATYRYNAQGQLLSATNAAGESEHYRYNDQHVIQERQLAGGATFFWEWEGEGKQSRCVHHWANFAQMDTRYVWEDNGTVTVHNSDGSQQVYQHDENARLVQQIDPDGAKHHKVYDEKGQLIAEKDPLGAITEYLHDEAGHLIAVIPPEDEPTYYEYHKGFVRTVSRGKAVWKYRRNAQGDITAQTDPDGNKTHYHYDNRGNLLSIQHPDGSRHELTWNPQGQLIEELLPDGGQRRYRYDALGRQITRQDESGAVTQYQWDAVGRLSQVTLPGGGTRAFTYNAYGKITAERDELGRVTRYEYADNLHLVSRRINPDGSQLRYRYDNARLLLTEIENERGEHYQLDYYPNGLIREETGFDGRTTAYAYDLNGHLTEKTEFGEDGSQLVTCYQRDAAGRLLVKTLPDDQKIHYAYDALGRLVSVDDGQWPLAYEYDLQDRLITEHQGWATFRYQYDALGQLSHCRLPDGSLLDYRYRHGGLLSAIDLNGQPLTAHQYLPGGREQYRQQGALLSHYHYDEQGRLQAHRISQQERSVYQRRYAYDANGNLAAIEDSRKGNKHFHYDPLDRLIAVRGDLPESFAHDPAGNLLSQTGQEGARLANVKGNRLLMQGDSHYDYDAYGNLTRERRGASQRLVTEYRYDSQHRLIAATLPDGRIAEYRYDAFGRRIAKIIDGQTTEFLWQGERLIAESGQNHYRSYVYEPGTFRPLAMLLGEGRAAEPYYYQLDHLGTPQELTSTAGSIVWSAKYRAYGNVAKLEVAELENPLRFQGQYFDQETGLHYNRHRYYNPNTGRFLTPDPIKLAGGLNNYQYVPNPTGWVDPLGLNNCPGQDLCNPNLNSSLSKDISYVNDGEPQPASPESKYKYLYRGDTRSQDIIFEDGFKSKGDSNDLQLHVYDNNEPPSNFISTSTSPEVGIDFGTAYRTKPGYLYTLRLLKGIDVNNEKGLKVPFPDEKEIAVPKEVKREDILGVTPLKADGSYKGYSIPNPRRI>ref|WP_017338151.1MNTPIGTLVLHVLDAKTPDVNLILKDFKNCLSDYKEWADGFLTGWALDVDQTFKVGNEVSVTKRKTKTGKVETYATCPLAGDFTLIHMFESARFVPIGNTPVKLEPVTKGTFYDDVTGPVIETTIGPSGIKVIDGCKKGQMYRITFFPNVSQSNVKALYDSYQGVIGNLNGWLQSEWSSKFQPQWASYSAADRAGRSVILLKRAMDGLEKALLRLWDDIKSLFELLAHPKENLEKLRKYLSDVEIDKLYAASKESIASGLLILSDEPLMFIYVSAIVAWVGMLPPQVIVDVVTAIASEFLINVVLGICLTGGAGIAVRVGTKTLSTVKSGTALKYLEELAAKLMSLSGKHSLPAHAEVSKPLIASAKSVPMKPTKTAALKIDEAAANGSKSQVPKTTDTPKQEVKDASSFPRGKKDKQTTLAPKEKVDDAPTQSKTPDDKSAACAKKTCTNGCPVSMVTGEELLTLTDGELGGLLPFSWTRLYRTSAAEIDCGLGYGWSHALAQRVDINGDEVIWTDHENRVTTFPLPSVQRPAITNSLSEAAIFLGDDPSELTLTQAGERTQFYHFRYNSKGATLIAISDSYDNRLHITRDIHGRIKRVDNGAGRALLVRYDRKHIVAIDYQQFSPADNLEDAWSTVQTLVTYGYDAQHRLIEAKNAAGEAERYAYNDQNVILERQLAGGASFYWEWEKEGKSARCIHHWASFSQMDAHYAWDDKGSVTVTNADGSEEVYTHDDQARLVAKVDPDGAEHLKAYDDKGRLIAEKDPLGAVTEYQYNEAGRLVAVIPPEDAPTTYEYYHGYVRVVNRGPAKWTYWRNDQGDITEQVDPDGNSTHYSYNRQGRLLEIRHPDGSRHQLGWNNLGQLLEEQLPDGGQRKYRYDALGRQITRQDESGAITHYQWNAANRLAQITLPGGATRAFTYNAYGKVTAERDELGRVTRYEYADNLHLVSRRINPDGSQLRYRYDNSRLLLSEIENERGEHYHLDYYPNGLIQQETGFDGRRTAYEYDLNGQLLKKIEFGDDGSELFTEYQRDAAGRLLVKTLADGDEIHYSYDALGRLVNVDDGHWPLAYEYDLQDRLITEHQGWGTLRYEYDNLGQLSHCRLPDGSKLDYRHQPGGRLSSIDLNGSRLTTHQFSAGREQQRQQGLLLSQYQYDEQGRLQAHTVSQQDRNLFRRRYAYDANGNLAGIDDSRKGKRSYHYDPLDRLINVRGTTPESFAHDPAGNLLGQGDQPSANLVNVKGNRLLMQGDRHYDYDAHGNLIRERRGTGQKLVTEYRYDCQHRLIGVSLPGGSIASYQYDAFGRRIAKTVDGHTTEFLWQGERLIAESADNRYRSYIYEPGSFRPLAMLDGEGSLKATPFYYQLDHLGTPQELTDYSGEIMWSAKYRAYGNLATLDVAEIDNPLRFQGQYFDAETGLHYNRHRYYNPGTGRFLTPDPIKLAGGLNNYQYVPNPTGWVDPLGLSCVPSNCPDDRRAELNQKYGRTGDINNDINARGRKETATEFYRTQGFEETSIPGHLNGIDFNQPVNVETLNRGKTVYQFQSPGAPQGNYYSLNPATTPSELGIGPLGDNRAAGTVELKLQGIYRTTEKTPVLKSTAKQIDDTWSVKGVTQPSSGGGTQVFSSAKGNFEKIP>ref|WP_123367297.1MNTPIGTLVLRVLDAKTPDVNLILKDFKNCLSDYKDWADGFLTGWALDVDQTFKVGNEVSVTKRKTKTGKVETYATCPLVGDFTLIHMFESARFVPIGNTPVKLEPVTKGTFYDDVTGPVIETTIGPSGIQVINGCKKGQMYRITFFPNVSQSNVKALYDSYQGVIGNLNGWLQSEWSGKFQPQWASYSAADRAGRSVILLKRAMDGLEKALLRLWDDIKSLFELLAHPKENLEKLRKYLSDVEIDKLYAASKESIASGLLILSDEPLMFIYVSAIVAWVGMLPPQVIVDVVTAIASEFLINVVLGICLTGGAGIAVRVGTKALSTVKSGTAIKYLEELAAKLMTLSGKHSLPAHAEVSKPLIASAKNVPMKPAKTAAIKIDEAAANGSKSQVPKTTDTPKQEVKDASSFPRGKKEKQTTLAPKEKVDDASTQSKTPDDKSATCAKKTCTNGCPVSMVTGEELLTLTDGELGGLLPFNWTRLYRTSAAAIDCGLGYGWSHALAQRVDINGDEVIWTDHENRVTTFPLPSVQRPAITNSLSEAAIFLGEDPSELILTQAGERTQFYHFRYNTQGATLIAISDDYGNRLHITRDIHGRIKRVDNGAGRALLVRYDRKHIVAIDYQQFSPADNLEDTWSTIQTLVTYSYDAHQRLIEAKNAAGEAERYAYNDQNVILERQLAGGASFYWEWEKEGKSARCIHHWASFSQMDAHYAWDDKGSVTVTNADGSEEVYTHDDKARLVAKVDPDGAEHLKAYDDKGRLIAEKDPLGAVTEYQYNEAGRLVAVIPPEDAPTTYEYYRGFVRVVNRGPASWKYWRNDQGDITEQIDPDGNSTHYSYSRQGRLLEIRHPDGSRHQLGWNNLGQLLEEQLPDGGQRKYRYDALGRQITRQDESGAITQYQWDAANRLAQITLPGGATRAFTYNAYGKVTAERDELGRVTRFEYADNLHLVSRRINPDGSQLRYRYDNSRLLLSEIENQRGEHYHLDYYPNGLIQQETGFDGRRTAYEYDLKGQLLKKTEFGDDGSELVTEYQRDTAGRLLVKTLADGEKIHYSYDALGRLVNVDDGHWPLAYEYDLQDRLITEHQGWGTLRYEYDQLGQLSHCRLPDGSKLDYRHQSGGRLSSIDLNGSRLTTHQFSAGREQQRQQGLLLSQYQYDEQGRLQAHSVSQQDRNLFQRRYAYDANGNLAGIDDSRKGNRSYHYDPLDRLINVRGTTPESFAHDPAGNLLGQGDQPTANLANVKGNRLLMQGDRHYDYDAYGNQIRERRGTGQKLVTEYRYDCQHRLIGVSLPGGSVASYKYDAFGRRIAKTVDGQTTEFLWQGERLIAESASNRYRSYIYEPGTFRPLAMLDGEGPLKAEPFYYQLDHLGTPQELTDYSGEIMWSAKYRAYGNLATLDIAEIDNPLRFQGQYFDAETGLHYNRHRYYNPGTGRYLTPDPIKLAGGLNNYQYVPNPTGWVDPLGLSDTCPTPDCKLPNNSANATKPDSIPMSQAKYDEVINLEKGNRPVDTREYLPEQYVNMHEEAFKQQGGSFVVIDDWIERSTYPTFPPRKFVGLPSEMDTVVAKYKASGGNWQVLNRELNLGTSDLSSAKIYLVKIKPDDPRFKYEIPNGNEAGAYPKEWVPGGETKSGTKEAALIGSEKINHGADMNKLLSQFDDWEQLQ>ref|WP_108183121.1MNTPIGTLVLRVLDAKTPDVSLILKDFKNCLSDYKDWADGFWTGWALDVDQTFKVGNEVSVTKRKTKTGAVETYATCPLVGDFTLIHMFESARFVPIGNTPVKLEPVTKGTFYDDVTGPVIETMIGPSGIKVVEGCKKGQTYRITFFPNVSQNDVKALYDSYQGVIGKLDSWLQSEWSSKFQPQWASYSAADRAGRSVILLKRALDGFEKALLRLWDDIKSLFELLANPRKNYEKLKKYLSDVEIDKLYAASKESIASGLLILSDEPLMFIYVSAIVAWVGMLPPQVVVDVMTAIASEFLINVLVGICLTGGAGLAVRAGTKVLSTVKSGEAVKYLEDLASTLMTLSNKHALPAHAEVSKPIIASAKNVPMKPAKTAALKIDEATTEAPKAGSRKAEPKSHVEGLPPKKQLPKTADTPKQEVKDASSFPRDKKKNQSTLQPKEKVDDVPDQSKTPDDKSATCAKKTCTNGCPVSMVTGEELLTITDGELGGLLPFSWTRLYRTSAAEIDCGLGYGWSHALAQRVDINGDEVVWTDHENRVTTFPLPSVQRPAITNSLSEAAIFLGDDPSELVLTQAGEKPLFYHFRFNSKGATLIAISDNYDNRLHITRDIHGRIKRVDNGAGRALLVRYDRKHIVAIDYQQFLPADNLEDAWNTVQTLTTYEYDIHHRLVKATNSLGESERYAYNEQNVILERQLAGGASFYWEWEKEGKSARCIHHWASFSQMDARYAWDDKGSVTVTNADGSEEVYSHDDKARLVAKVDPDGAEHLKVYDDKGRLIADKDPMGAVTEYQYNEAGRLIAVIPPEDAPTTYEYYKGFVRVVNRGTAKWIYWRNDQGDITEQIDPDGNSTHYTYDRQGRLLEIRHPDGSRHQLVWNNLGQLLEECLPDGGQRKYRYDALGRQITRQAETGAVTHYQWDAANRLAQITLPGGATRAFTYNPYGRVTAERDELGRITRYEYADDLHLVSRRINPDGSQLRYRYDNARLLLTDIENERGEHYQLDYYSNGLIQQETGFDGRRTAYEYDLNGKLLKKTEFGDDGSELVTGYQRDTSGRLLVKTLADGEEIHYSYDALGRLVNVDDGHWPLAYEYDVQDRLITEHQGWGTTRYEYDNIGQLSQCRLPDGSTLDYRHLSGGRLSSIDLNGSRLTTHQFDAGREQQRQQGVLLSQYQYDEQGRLQAHTVGQQERNLFQRRYAYDANGNLAGIDDSRKGNRSYHYDPLDRLVSVRGAIPETFAHDPAGNLLGQNDLPAANLANVKGNRLLMQGDRHYDYDAYGNLSRERRGAGQQLVTEYRYDCQHRLIDVTLPGGSTASYKYDAFGRRIEKTVDGQTTEFLWQGERLIAESAENRYRTYIYEPGSFRPLAMLDGEGPLKATPFYYQLDHLGTPQELTDYSGEIMWSAKYRAYGNLATLDVAEIDNPLRFQGQYFDAETGLHYNRHRYYNPGTGRFLTPDPIKLAGGLNNYQYVPNPTGWVDPLGLNCVPGECPDEVPAPEKDVRKFSSTKYKPEEVLGRKVYKNTMDVEPGVPTTIDKSVDPRIRKKIEEDGWSNLDLMKNGNAPIGPDGKYINLHHVTGDEPGPMVELTGTVHKKYHKELHGTIEDGDSFRNDPKLELQYSKFRKAYWKERAKDFE>ref|WP_019366452.1MIPRLGSFSFIGLGAESPDASKVISEFKSCLGTYKELAESLWTGWALDADQTFKIGDELSISKDKDYTSPVTNYATCPLNGEFTLVHAFEAARFVPIGNTPVRLEPVTRAWNGDEVTGPVINDEIGPSGIKVISGCKRGQLYRITFFPNVTKAQVEALYSSYQGVIQKLAAWLQSEWTNQFQPLWSTYQSANSGQRLKLQLEAALEGFEHALLSLWDDIKSLFQLIAHPYENAKKLAKYLSPDELKKLYTASKESLSTALLIASDEPLMFIYVAAIVSWVKLLPPQTCVEVLAQFTGELLINIVVGIILSGGAGLAIRVGTKALETLKSGQATALISKLADTLINASQSHALSVHADTLKPLAAHGEISEANTARKINAKVAPASKRPTPADTPSQTIENASTTSRNKTRRQTRLSREEKVDDVSKPAKDPNDKPSQECAKTCTNGCPVSMVTGEELLTLTDGELDGVLAFPWSRLYRTSAIEHNVGLGYGWSHSLAHRLEIQGDTVIWTDHENRRTEFPLPSQQRPAITNRLSAAAIYLGEDLSELVLAQAGAKPHFYHFRRIGQSARLISISDTYNNVLRISRDARDRIVRLTNTAGRALRFTYEHAHIVAVDYQVYRPADNEEASWHTVQTLATYRYNAQGQLLSATNAADESEHYRYNDQHVIQERQLAGGATFFWEWEDEGKQSRCVHHWANFSQMDTRYVWEDNGTVTVHNSDGSQQVYQHDENARLVQQIDPDGAKHQKIYDEKGQLIAEKDPLGAITEYQHDEAGHLIAVIPPEDEPTYYEYHKGFVRTVSRGKAVWKYRRNAQGDITAQTDPDGNTTHYHYDSRGNLLSIQHPDGSRHELTWNPQGQLIEELLPDGGKRRYRYDALGRQITRQDESGAVTQYQWDAVGRLSQVTLPGGGTRAFTYNAYGKITAERDELGRITRYEYADNLHLVSRRINPDGSQLRYRYDNARLLLTDIENERGEHYQLDYYPNGLIREETGFDGRTTAYAYDLNGHLTEKTEFGEDGSQLVTCYQRDAAGRLLVKTLPDDQKIHYAYDALGRLVSVDDGQWPLAYEYDLQGRLITEHQGWATFRYQYDVLGQLSHCRLPDGSLLDYRYRNGGLLSAIDLNGQPLTAHQYLPGGREQYRQQGALLSHYHYDEQGRLQAHRISQQERSVYQRRYAYDANGNLAAIEDSRKGNKHFHYDPLDRLIAVRGDLPESFAHDPAGNLLSQTGQEGARLANVKGNRLLMQGDSHYDYDAYGNLTRERRGAAQRLVTKYRYDNQHRLIATTLPDGRVAEYRYDAFGRRIAKTIDGQTTEFLWQGERLIAESGQNHYRSYVYEPGTFRPLAMLHGEGKAAEPYYYQLDHLGTPQELTSAAGSIVWSAKYRAYGNVAKLEVAELENPLRFQGQYFDQETGLHYNRHRYYNPNTGRFLTPDPIKLAGGLNNYQYVPNPTGWVDPLGLNNCPGIASCSTGTAETPTANINSGEPNAPSPERSREERQSKIERLSEANAKRRVKEYEERYDMHTITKHGPEIPRDKLKQRAIDGTDPSNGELPKKAKGNPSSQFKNWKLQLHAINAALTREARGLPLHTGVDHKGNNIVRVDLPGAGRGYKPNKKDPQNPKFNENMNGAEVKFDKDNTRRPFTAFPVDNL>ref|WP_117163635.1MSVLMGGISGALTAKQPDAQAIINDFKKCLTDYREHAEAWYGGLLDAEQQFKVGDEVGTADKDSKQSNTLYANCPANGKLKLVHSFESARFVPIGNTPVRLVPVEDGRVYGKNEVGQAIDLKIGPSGILEVTGLKPNQQYAITFFPNPTRAQIDSLFNSYQGVIGDLSGWLQTEWSTDFLPLWQAHTNASMGGRALQQLESAWKGFLKAIMGLWGDIKGLYDLVAHPRENYEKLKKFFTEEEIKKIYNASKEAIQTALLIASDEPLMWIYVAAIVAWVKMLPPQTCTEVLAELTTEVLLNILIGIVLTGGIGLAVRVGAKGLNAAKNSGKVMKLIEDFTSMLMKISKKNATGHAETAKPLLLNGNAPMNPARKASVEIAPPKPAETAVAPKKKPPVTGGAVETDAQIQARAKKENATRIEQQEKVDDAAKQSKTPADQPAQCADRTCTNGEPVSMVTGEELLTLTDGELVGLLPLEWTRLYRSSAVEIDSRLGYGWSHSLSHRLQLDDEGVLWTDNENRQTRFPMPTEQRPAITNSLAQAAIYLGDAPGELILTQAGPKARFYHFRAGRLTTISDAYDNQVHISYDLVDRIQRIDNGAGRALLLRYDDRHIVAVDQQQQRSEFDERGERQNPWLTIQTLVTYSYNARHQLVSATNAVGETEYYRYNDQHVILERQLAGGASFFWEWEREGKLSRCVRHWANYSQLEARYEWDDKGTVKVHNADGSEQVYVHDENARLVSETTPDGAETQKAYDDKGRLIAVKDPLGAITEYQYNDAGRLIAVIPPEDATIRYNYFDGQLVEVLRGKARWQYDRNQQGDITRQIDPHGNETHYRYDRQGRLLEIRHPDGSLHQLGWNGLGQLLEERLPDGGQRKYRYDALGRQITRQDETGSITHYQWDAANRLAQVTLPGGATRAFTYNPYGRVTAERDELGRVTRYEYADNLHLVSRRINPDGSQLRYRYDNSRLLLTDIENERGEHYQLDYYANGLIQQETGFDGRRTAYEYDLNGQLLKKTEFGDDESELVTEYQRDAAGRLLVKTLPDGEEIHYSYDALGRLVNVDDGNWPLAYEYDLQDRLITEHQGWGTLRYEYDSVGQLKHCRLPDGSKLDYHHHRGGQLSSIDLNGSRLTAHQFSGGRERQRQQGLLLSQYQYDEQGRLQAHSVSQQDRSLFQRRYAYDANGNLAGIDDSRKGNRSFHYDPLDRLINVRGATPETFAHDPAGNLLGQGDLPTANLANVKGNRLLMQGDRHYDYDAYGNQIRERRGAGQKLVTEYRYDAQHRLIGVSLPGGSTATYKYDAFGRRIAKTVDGHTTEFLWQGERLVAESAENRYRSYIYEPGSFRPLAMLDGEGPLKATPFYYQLDHLGTPQELTDYSGEIMWSAKYRAYGNLAALDVAEIDNPLRFQGQYFDAETGLHYNRHRYYNPGTGRFLTPDPIKLAGGLNNYRYVPNPTGWVDPLGLATVPVDCPGATPKRPNNSANATKPDHLPMTQEKFDEIIDMDKSDRPPNPGDYLPDSYISSHRALFEKEGGSFVVVESWITDSRFPSFPARKFVGLSSEMEEVVAKYRAAGNDWRVLNNELNLGSNLEGEGIYLVRIKPGDPRFSYEMPNGKENGAYPKQWVPGGATKSGTSEAALVGSEKIVHDQDIDTLMKHFEDWEKLQ>ref|WP_110680648.1MNFVIGSLIGQLNAKQPDVQVILKQFRNCLHDYREKSEAWYGGVLDAEQQFKVGDEVDTQDKDDKDPATLYATCPANGKLTLVHSFEAARFVPIGNTPARIVAVEDSSFFGKNEIAKAIEVTIGPSGIKEVPGCKPGQQYKVTFFPNPTRAQIDALYASYQGVIGQLEGWLNGEWSSQFLPLWRAHNAASMSERALQQLEAAWKGLVKVIMGLWEDIKSLYDLVAHPRENYQKLRKFFTEEQIKRIYNASKEGLETALLIASDEPLLWIYVAAIVAWVSMLPPQTCIEVLAELTGEVLLNILLGVVLTGGIGLAVRVGTKALTAAKNGGRVVKLIEDFTSMLMKFSQGHAKSHASSAKPLLAHGSGGMQPTRKATLKIDPPTPAQSAQPPAKPTGRRSGGKPSQPPSQVPPSAAAKTGTPQIEPDATVYPRSKPQNATSLKQVEPVDDASAPAQTPNGKPADQADKTCTNNCPVSMVTGEELLTLTDGTLEGVLPFTWDRLYRTSAAELDCGLGFGWSHSLAQRLELDGDQLVWTDHENRRTRFPIPSEQRPAITNRLSRAAIYLGQDPAELILAQAGEAPRFYHFRRDALGGYLIAISDAYGNRLSLSRDLAGRLQRLSGAGRALLLRYEGSHIVAVDYQIQRPAENAEASWHSVQTLVRYQYNAQGQLIGASNALGETEHYRYDDQHVILERRLAGGAAFYWEWQGQGKQARCVHHWANFGQMDARYTWDDQGSVTVVNADGSEETYVHDQNARLVRQVDPDGAETLRSYDDKGQLVAEQNPLGAITEYHYDEAGRLESRIPAEGEAVHYSYFDGQVRSVRRGKAIWKYERNAQGDITEQIDPLGQSTRYRYDARGRLLEVQHPDGSQHLLVWNAQGELIEEQLPGSGIRRYRYDAFGRLITRQDEHGALTQYQWDAVGRLLQVTLPGGTSRAYSYNAYGKVTSERDEQGRVTRYEYADGLHLVSRRINPDGSQLHYRYDNARLLLTAIENERGEHYRLDYHPNGLISQETGFDGRRTAYRYDLAGHLIEKKEFAEDGSERITAYQRDSAGRLLVKTLPDGEQIHYAYDGLGRLVSVDDGQWPLAYAYDLRDRLIEEHQGWATLRYEYDALGQLGHCRLPDGSRLDYRYRPGGELAGIDLNGQPLTRHQFDKGRERYRRQGTLLSEYDYDEQGRLQAHRVRSDHRQLLQRQYRYDASGNLAAIEDSRKGSRSFHYDPLDRLVAVRGDLPESFAHDPAGTLLAQNSAASEGLANVKGNRLLMQGDSHYDYDAFGNLVRERRGTGQKLVTEYRYDSQHRLIAASLPDGRQVQYRYDAFGRRIAKTVDGQTTEFLWQGERLIAESGPAHYRSYVYEPSTFRPLALLQGEGEASQPFYYQLDHLGTPQELTSAGGQILWSVKYRAYGSLAKVEVAEIDNPLRFQGQYFDAETGLHYNRHRYYNPGTGAFLTPDPIKLAGGLNNYQYVPNPTGWVDPLGLAVVKGDCPAPDRAQLNEKYGRTGNLNDDINSRGRKEVATEFYRAQGFSDDSIPGHLNGIDFNKPVSIETLNRGKTVYQFQSPGAPQGNYYSLNSETSPSELGIGPLGDNRAAGTVELKLQGIYRTNQKTSALKSTAKAIDDTWSVKGVTQPSSGGGTQIFTSKKSNFEKIQ>ref|WP_050682437.1MNTPIGTLVLRVLDAKTPDVNLILKDFKNCLSDYKEWADGFLTGWALDVDQTFKVGNEVSVTKRKTKTGKVETYATCPLAGDFTLIHMFESARFVPIGNTPVKLEPVTKGTFYDDVTGPVIETTIGPSGIKVIDGCKKGQMYRITFFPNVSQSNVKALYDSYQGVIGNLNGWLQSEWSSKFQPQWASYSAADRAGRSVILLKRAMDGLEKALLRLWDDIKSLFELLAHPKENLEKLRKYLSDVEIDKLYAASKESIASGLLILSDEPLMFIYVSAIVAWVGMLPPQVIVDVVTAIASEFLINVVLGICLTGGAGIAVRVGTKTLSTVKSGTALKYLEELAAKLMSLSGKHSLPAHAEVSKPLIASAKSVPMKPAKTAAIKIDEAAANGSKSQVPKTTDTPKQEVKDASSFPRGKKEKQTTLAPKEKVDDASTQSKTPDDKSAACAKKTCTNGCPVSMVTGEELLTLTDGELGGLLPFSWTRLYRTSAAEIDCGLGYGWSHALAQRVDINGDEVIWTDHENRVTTFPLPSVQRPAITNSLSEAAIFIGDDPSELILTQAGERKQFYHFRYNSKGATLIAISDNYDNRLHITRDIHGRIKRVDNGAGRALLLRYDRKHIVAIDYQQFSPADNLEDAWSTVQNLVTYGYDAQHRLIEAKNAAGEAEHYAYNEQNVILERKLAGGASFYWEWEKEGKSARCTHHWASFSQMDAHYAWDDKGSVTVTNADGSEEVYTHDDQARLVAKVDPDGAEHLKAYDDKGRLIAEKDPLGAVTEYQYNEAGRMVAVIPPEDAPTTYEYYRGFVRVVNRGPAKWTYWRNDQGDITEQVDPDGNSTHYSYNRQGRLLEIRHPDGSRHQLGWNNLGQLLEEQLPDGGQRKYRYDALGRQITRQDESGAITHYQWDTANRLAQITLPGGATRAFTYNAYGKVTAERDELGRVTRYEYADNLHLVSRRINPDGSQLRYRYDNSRLLLSEIENERGEHYHLDYYPNGLIQQETGFDGRRTAYEYDLNGQLLKKTEFGDDGSELVTEYQRDAAGRLLVKTLADGEEIHYSYDALGRLVDVDDGHWPLAYEYDLQDRLITEHQGWGTLRYEYDNLGQLSHCRLPDGSKLDYRHQPGGRLSSIDLNGSRLTTHQFNAGREQQRQQGMLLSQYQYDEQGRLQAHTVSQQERNLFRRRYAYDANGNLAGIDDSRKGNRSYHYDPLDRLINVRGTTPESFAHDPAGNLLGQGDQPTANLANVKGNRLLMQGDRHYDYDAHGNLIRERRGAGQKLVTEYRYDCQHRLIGVSLPGGSVASYKYDAFGRRIAKTVDGHTTEFLWQGERLIAESADNRYRTYIYEPGSFRPLAMLDGEGPLKATPFYYQLDHLGTPQELTDYSGEIMWSAKYRAYGNLATLDVAEIDNPLRFQGQYFDTETGLHYNRHRYYNPSTGRFLTPDPIKLAGGLNNYQYVPNPTGWVDPLGLSSTCPGPDCKLPSNSANATKPDHLPMSQEKFDEIIDMNKSDRPPNPGDYLPDNYISAHRELFAKEGGSFVVVESWIKGSKFPSFPPRKFVGLSSEMEDVVAKYRAAGNDWRVLNNELNLGAKSLEGDGIYIVRIKPDDPRFSYEMPNGKENGAYPNEWVPGGATKSGTKEAALIGSESVTHDSDVGTLLKNFDDWEKLQ>ref|WP_058958962.1MNTPIGSLVLRVLNAKTPDVSLILKDFNNCLSDYKEWADSFWTGWALDVDQTFKVGNEVSITERKTKIGAVETYATCPLAGDFTIIHMFDSARFVPIGNTPVRLEPVTKGYVYDDVTGPVIETTIGPTGIKIIDGCKKGQMYRITFFPKVSQSDVKALYDSYQGVISKLSGWLQSEWSGKFQPQWATYSAADRTGRSVILLKRAMDDLEKALLRMWDDIKSLFELLAHPKENYEKLRRYLSDVEIDKLYTASKESIASGLLILSDEPLMFIYVAAIVAWVGMLPPQVVVDVVSAIASEFLINVVVGICLTGGVGIAVRVGTKVLSTVKSGEAVRYMEELAATMMRLSSEHSLPAHAEVSKPLIVSARNVPMNATKTGEIKIDEAAANGSKSQVPKTTDTPKQEVKDASSFPRKKTEKQTTLEPKEKIDDAPDQSRTPDDKSAASADKTCTNGCPVSMVTGEELLTLTDGQLGGLLSFEWTRLYRTSAAEIDCGLGYGWSHALAQRVDINGEEVIWTDHENRVTTFPLPSMQRPAITNSLSEAAIFLGNDPSELILTQAGEKARFYHFRYDKKGATLIAMSDSYDNRLHITRDIHGRIKRIDNGAGRALLVRYDRKHIVAIDYQLFSPADNLEDAWSTVQTLVSYEYDAQKRLINATNAAGEAERYRYDEHNVILERQLAGGASFYWAWEKEGKSARCIHHWASFSQMDAHYVWDDKGSVTVTNADGGEEVYTHDDKARLVAKVDPDGAEHLKAYDDKGRLIAEKDPLGAVTEYQYNEAGRLTAVIPPEDLPTTYEYFHGFVRVVNRGKAKWVYWRNDQGDITEQVDPDGNSTHYRYDPQGRLLEIRHPDGSRHQLGWNGLGQLLEERLPDGGQRKYRYDALGRQITRQEETGAITHYQWDAANRLAQITLPGGATRAFAYNAYGKVTAERDELGRITRYEYADNLHLVSRRINPDGSQLHYRYDNARLLLTEIENERGEHYHLDYYSNGLIQRETGFDGRSTAYEYDLNGQLLKKTEFGDDGSELVTEYQRDSAGRLLVKTLADGEEIHYSYDALGRLVSVDDGHWPLAYEYDLQDRLITEHQGWGTTRYKYDKLGQLSHCRLPDGSKLDYHHQRGGQLGSIDLNGSRLTTHQFSSGREQQRQQGLLLSQYQYDDQGRLQAHSVSQQDRHLFHRHYAYDANGNLAGVNDSRKGNRSFHYDPLDRLINVRGSTPESFAHDPAGNLLGQGDQPAANLANVKGNRLLMQGDRHYDYDAYGNQGRERRGTGQKLVTEYRYDCQHRLIGVSLPGGSVASYKYDAFGRRIEKTVDGHTTEFLWQGERLIAESADNRYRTYIYEPGTFRPLAMLDGEGPLKAAPFYYQLDHLGTPQELTDYSGEIMWSAKYRAYGNLATLDIAEIDNPLRFQGQYFDAETGLHYNRHRYYNPGTGRFLTPDPIKLAGGLNNYQYVPNPTGWVDPLGLSADCPGNKNDSIESRKHEPEPPELSRSGAFKQAKSEAGIPKSQHPDQIYDPTTGKTQQYRYVRMTDRAGESILNSEGKPTLTREYQYTRGDGSKIIIQDHSAGHQYHEANKVGDQKAHFNLRPIENPRTGKVPGAKDHYYFKD>ref|WP_058783083.1MTTPRPPLGTLPITLAATQPDIRAVVQDFRTCLVDYRGFAEAWYGGVLDVEQKFTVGQEVVTRAKDSKRQVSLYATCPVDGKLTLIHCFEAARFVPIGNTPVSIVPVNEGQGPRRSGAAPAGAAQSAVIGADGTYTFAGCTPYQRYRVTFFPNVTGAQLETLYGSYQGVIDDLQAWLQQEWTQKHQPSWATYRSASAGGKVGLQLEAAWNGLLRSILNLWNDITQLFDVIAHPRKHLAKLQGLLDPQVLEQLYNAGKEQLHTALLIASDEPLLWIFLCALIAWVKLLPPTTAAEVVGALTGEVLLNVLIGVVLTGGLGLAVRLGGKLAQVAEGGRALGLLQELAEALMQRCARHAKPHSEAAKPILAQGSADLDASRKATVELKNGAAVEQIRADAAPYARGKRRKATRLSQESRVDDVSIPARTPSGKPAEPAALTCTDGCPVSMVTGEELLTLTDAELDGPLPFAWTRLYRSSACDSDLGLGPGWSHALAHRLERQGEQLVWIDQENRRTPFPLPSQQRPAISNRLARAALYLGEQPDELILAQAGDSPRFYHFRRLELIAISDAYGNRLTVERDAQGRIQRLGNGLRALRLDYRQGRIAAVDYQVRQPGDHHEASWHSLQTLVRYRYAANGQLLAASNALDETERYVYDDRHVILERHLAGGAAFFWEWQGEGRDARCVRHWSSLGQLDSRYTWTDDGSVQITFSDGSQQHYTHDANARLVRQVDPDGAETQKTYDAQGQLLSERDPLGALVRYEYDAAGQLLARYPAQDEPTYYSYCDGQLLSLTRGDATWRFEHNALGDLTARTDPDGHTTRYRYTAQGRLAAIEHPDGSCHSFTWNVAGQLIEEQLPDGGIRRYRYDALGRQTYHQQENGAITHYEWDALGRLRQLKLPGGASRAFNYNAYGKVTAERDELGRITRYEYANGLHLVTRRLNPDGSTLSYRYDHARLLLTDIENERGEHYQLDYHPNGLIRSETGFDGRTCSYAYDLAGNLSEKTEHGSDGSTLTTRYERDREGRLQRKVLPDGSQVDYAYDDLGRLIGVDDGQWPLAYRYDLQDRLIEEHQGWASFTYGYDACGQLSHSRLPDGNRLHYYHGAGGALAGIDLNGQPLTRHQHAPGGLELQRQQGALSSHYRHDDQGRLLAHRLENRAERLLERRYHYDASGNLLGIDDSRKGQTRYLYDPLDRLVAVRGELPESFAHDPAGTLLGGGEAGSIQGNRPLLHGDRHLDYDAHGNLVRERRGRAQALVTHYRYDSQHRLILATLPDGSEAHYRYDAFGRRIAKTVAGRTTEFLWQGDRLVAESGPDGYRSYLYEPGTFRPLALLVGEGPQAVTPYHYHLDHLGTPQELTDASGTLAWSARYRAYGNLARLDIEQIAQPLRFQGQYHDPETGLHYNRHRYYHPETGSFITPDPIRLAGGLNSYRYAPNPTGWVDPLGLANVKGQCPGGENDKRPDKYYESRRAAFRQAKQDAAIPASENPTKIGRVKLTSAGETVYNSGGPVLTREYHYNNIYGDRVVIQEHSHGHSEFPQDTASGKPHFNVREFNPETGNGHRTKTLNLRAIAKHYVFE>ref|WP_122316998.1MSILMGEIAGNLTATQPDAQALINNFKKCLTDYRKHAEAVYGGLIDAEQQFKVGDEVGTADKDSKAANTLYANCPANGKLKLVHSFEAARFVPIGNTKVRLVPVEDGKLYGKNEVGKALEYTIGPSGILEVTGLKPNQQYEITFFPNPTPAQINSLFSSYQGVIGDLSGWLQTQWSTEFLPLWQAHTNASMAGRSLQQLESAWKGFLKAIMGLWSDIMGLYDLVAHPRENYEKLKKFFTEEEIKKIYNASKKAIETALLIASDEPLMWIYVAAIVAWVKMLPPQTRTEVLAELTTEVLLNILIGIVLTGGIGLAVRVGAKGLNAAKNSGKVMKLIEDFTAMLMNLSKKNAAGHAETAKPLLLNGNAAMNPARKASVEIAPPKPAETAVAPKKKPPVTGGTVETDAQIQARAKRENATRMEQAEKTDNAPEQSKTPADQPAQCADKTCTNGEPVSMVTGEELLTLTDGELGGVLPFEWTRLYRSSAVEVDSRLGYGWSHSLSHRLQLDDDGVLWTDNENRQTRFPMPTEQRPAITNSLAQAAIYLGTAPGELILTQAGPKPRFYHFRAGRLTTISDPYDNQVHISYDLVDRIQRIDNGAGRSLLLRYDDRHIVAVDQQQQRSEYDERGERQDPWLTLQTLVTYAYNDRQQLVSATNAVGETEYYRYNDQHVILERQLAGGASFFWEWEREGKFSRCVRHWANYSQLEARYEWDDKGTVTVYNADGSEQVYVHDENARLISETAPDGAETQKAYDEDGRLIAEKDALGAVTEYQYNTAGRLVAVIPPEDAPTHYNYFDGQLVEVRRGKARWQYDRNDQGDITRQVDPHGNETHYRYDLHGRLLEIRHPDGSRHTLGWNGLGQLLEEHLPDGGQRKYRYDALGRQITRQEETGAITHYQWDAVNRLSQVTLPGGATRAFSYNPYGRVTAERDELGRVTRYEYADNLHLVSRRINPDGSQLLYRYDNSRLLLTEIENERGEHYHLDYYSNGLIQQETGFDGRRTAYEYDLNGQLLKKTEFGDDGSELVTEYQRDSAGRLLVKTLADGEKIHYSYDALGRLVNVDDGHWPLAYEYDLQDRLITEHQGWGTTRYEYDKLGQLSHCRLPDGSKLDYRHQAGGQLSSIDLNGSRLTSHQFAAGREQQRQQGLLLSQYQYDEQGRLQAHSVSQRDKHLLQRRYAYDANGNLAGIDDSRKGNLRYHYDPLDRLINVRGATPESFAHDPAGNLLGQGDQPAANLANVKGNRLLMQGDRHYDYDAYGNQIRERRGAGQKLVTEYRYDVQHRLIGVSLPGGSTASYKYDAFGRRIEKTVDGHSTEFLWQGERLIAESAENRYRSYIYEPGSFRPLAMLDGEGPRKATPFYYQLDHLGTPQELTDYSGEIMWSAKYRAYGNLAALDISEIDNPLRFQGQYFDAETGLHYNRHRYYNPGTGRFLTPDPIKLAGGLNSYQYVPNPTGWVDPLGLNNCPGATMHNGVPISNNARGHLENIDGYSTKTGVKGAHNRNDFLQAATDKNLHIISETQSPTTPGLSEIVYGRDSLDRAGNVVGVKQFGNPKTVYDPSVISTDRMFEAGKEAAASGYSQAKASGNRVYSASHDGIIFRIYLDETLTTVTNFHPTMK>ref|WP_082228549.1MSVLMGGIVGGLTAKQPDAQAIINDFKKCLKDYREHAEAWYGGILDAEQQFKVGDEVGTADKDSKKENTLYANCPANGKLVLIHSFESARFVPIGNTPVQLTPVVNGRFIGKNEVGPTINKTIDASGILEVSGLTPNQQYKITFFPNPTRAQIDSLFNSYQGVIGELGGWLQTEWSTSFLPLWQAHTDASLGGRALQELEAAWEGFMKAIMGLWGDIKSLYDLIAHPRENYEKLKNFFTEEQIKKIYDASADAIHTALLIASDEPLMWIYVAAIVAWVKMLPPQTCTEVLAQMSTEFLLNILIGVVLTGGLGLAVRVGTKAVQGVQSSGKVIKLIEDFTGMLMKVSKSKATPHTEASKPLLLNGDSKFNPARKADVQIAPPKPAETATAPKNRPLITGGSDSNFNPTPKADVQIAAPKPAETATAPKSKLPGTVGKVESDAQIQTRKKDEPASRIEQVETVDNAPEQPKNPAKKPAQCVDDTCSDGEPVSMVTGEELLTLTDGELGGLLPFEWTRLYRSSAVEIDSRLGYGWSHSLSHRLQIDDEGVLWTDNENRQTRFPMPTEQRPAITNSLAQAAIYLGNEPGELILTQAGAKTRFYHFRAGRLTAISDAYENRLHISYDFVDRIQRIDNGAGRALLLRYEDRHIVAVDQQQQRPEYNERGERQDPWLTIHTLVTYRYNALNQLVSSTNAMGETEHYRYNDQHVILERQMAGGASFFWEWEREGKLSRCVRHWANYAQMEARYEWDDNGSVIVHNADGSELVYVHDENARLVSETAPDGGETQNAYDDNGRLIAVKDPMGAITEYQYSDAGRLIAVIPPEGVPTRYNYFNGQLIDVQRGKARWKYERNRQGDITQQTDPDGNETFYSYDRQGRLLEIRHPDGSRHQLGWNNLGQLLEERLPDGGQRKYRYDALGRQITRQDEFGAITQYQWDAVDRLTQVTLPGGATRAFTYNPYGRVTAERDELGRITRYEYADNLHLVSRRINPDGSQLRYRYDNSQFNLTEIENERGERYQLDYYPNGLIQQETGFDGRRTAYEYDLNGQLLKKTEFGDDGSELVTEYQRDAAGRLLVKTLPDGEEIHYSYDALGRLVNVDDGHWPLAYEYDVQDRLITEHQGWGTTRYAYDKVGQLSHCRLPDGCTLDYRHLSGGRLSSIDLNGSRLTSHQFNAGREQQRQQGLLLSQYQYDEQGRLQAHSVSQRDKHLFQRRYNYDANGNLAGINDSRKGNRSYHYDPLDRLISVRGAMPESFAHDPAGNLLGQNDLPAANLANVKGNRLLMQGDRHYDYDAYGNLIRERRGNGQKLVTEYRYDCQHRLIGVSLPGGSTATYKYDAFGRRFEKTVDGHTTEFLWQGERLIAESAENRYRSYIYEPGSFRPLAMLDGEGPLKAAPFYYQLDHLGTPQELTDYSGEIMWSAKYRAYGNLAALDVSEIDNPLRFQGQYFDAETGLHYNRHRYYNPGTGRFLTPDPIKLAGGLNSYQYVPNPTGWVDPLGLSNNCPGKAKKGFQRKVDDTSNNKVENGEPELPKGHDSTITRKAAFRQAKRVGGVPLSQQPSRSYKEVLTDQPGRVMSRVYEFNLKDGRIVTIREHSLGHIKGNEGPHFNTEVTGTTGTKAALKEGDNSHTYFKKD>ref|WP_124200354.1MISSFYAMFMLRGVNAKQPDASQLIGEFSSGVLNRYEEYANSWLWGWALDVQQTIALSDGNGNELTRLKADKDANILEDVASCPVNGQLKLLHTFESTSFVPIGNTPYEITRIGSRTPQFKGVLDADGMASISGCAPNTLYQVKFYPEVSIQEIDALYRSYDDVIAKLNEWLLEQWNGPLSAEWKRLAPVGDRRRMEQINDAYLDGMGKALVNLWDDVVELFGILANLDEHAAKLLEFINEADFEKLKAQAKELLGTALLIASDEPLLFIYASAIVCWTQLLPPTTAAEMVAELSTGLLLDILLGIILTGGAGLAVRLAVKGSKAVKASEGAMLLRKAMQVLIDTSCRHSLQLHAEKLKPLAVRGEVMANSSNKITAQIQSTAQGIARTEPDTLPLSRGKRANTSLSSSQATPDASAPGRTPAGKPADQAAQTCVKDCPVSMVTGEELLTLTDCELDGPLRFAWTRLYRTSAAEIDNGLGFGWSHALSHRLDRDGDELVWTDHENRRTRFPIPDAQRPAITNRLSRAAIYLGDDPSELVLAQAGEKPRFYHFCKDPDGAYLIAISDAYGNRLELSRDRLGRPRRLSHKAGRALLLCYQGRHIERVEYQVYQPADNLEACWHTAQVLARYRCNERGQLIEAGNALYEAERYRYDARNVILERQLAGGASFFWEWEGEGKQARAVHHWASFPQMDSRYVWNEDGSVTAINADGSEEVYVHDDNARLVRQVDPDGGETLRHYDEKGQLVAERDPLGTITEYRYDQAGRLEALLPAEGEPTCYSYFDGFVRSVRRGEAQWKYERNAQGDITRQTDPEGNVTHYAYDHRGCLVEITHADGSLHQLTWNPLGQLIEEQLPDGSVRRYRYDTLGRQITRQDESGAITRFQWDAAGRLSQITLPGGASRMYRYNAYGKVTSECDEQGRVTRYEYLDDLHLVSRRINPDGSQLRYRYENARLLLSEIENERGERYRLDYHGNGLISQETGFDGRRTAYRYDLNGQLLEKTEYGDDGSELRTTYQRDSTGRLLAKTLPDGNRVDYRYDTLGRLVAVDDGTWPLAYEYDLRDRLVREHQGWATLHYAYNALGQLIHCRLPDGNRVDYRYQTGGTLSAIDLNGQSLTRHQFGSGRERQRQQGELLSQYHYDEQGRLLAHQVSQRRHHLYQRQYRYDTSGNLAAIEDSRKGIRSFRYDPLDRLLGVRGETPESFVHDPAGNLLAQGGQFDARQMEVRGNRLLTQGDRHFDYDAYGNLVRERRGTGQKLVTEYSYDCQHRLIGVSLPDGRQVAYRYDAFGRRIAKQVDGRNTEFLWLGERLLAESGDRHYRTYLYEPDSFRPLALLDGEGPEQVEPCYYQLDHLGTPQELTRADGRLCWSARYRAYGNVLKLDIAEVDNPLRFQGQYFDAETGLHYNRHRYYNPSTGRYLTPDPIRLAGGLNSYRYVPNPTGWVDPLGLASSKTRCPEALDNNPRKGINSPSGSKKPAGPIVELDAQGNEIYYRTLSEQHLEILRNNFEVPPTSETFISPLQSYSQEYDGKLVRLTASPGTMNELSKIGVTANSGTGLLLSDLPPARKGWKQNNALFKLEALKKPTINEGGGVINTGLGDGKALEIFNKNLIDFEVID>gb|EKN46199.1MNISMGSIVGNLTAEQPDAQVIIKDFKKCLADYRKHAEAWYGGILDAEQQFQVGDEVGTQDKDSKSSADLYATCPANGKLKLVHSFESARFVPIGNTPVRLVPVVDGLFMGKNEAGGPINVTIGPSGIKEVTGLKPNQQYKITFFPNPTPAQIDSLFNSYQGVIGELSGWLTTQWSTDFLPLWQAHSDASMTGRAMQQLDSAWKGFLKAIMGLWGDIKSLYNLVAHPRENYEKLKKFFTEEEIKRIYNASKEAIHTALLIASDEPLMWIYVAAIVAWVKMLPPQTCTEVLAELTTEVLMNILIGVVLTGGIGLTVRVATKALKATQNSGKVMALIEDFTSMLMKLSRKNAQAHAETAKPLLLNAEAKLNPARKTDVAIAPPKPAVTATAPKKKPPISGTVEPDAQVQARGKKKNATKVEQKEHVDDAPAQSKNPADEPAEPAKKTCTNGDPVSMVTGEELLTLTDGELGGLLNLEWTRLYRTSAVEIDSRLGYGWSHSLSHRLQLDDEGVLWTDNENRQTRFPMPTERRPAITNSLASAAIFVGDAPGELILTQAGQKPRFYHFRAGRLTTISDAYDNQLHIEYDLVDRIQRIDNGAGRSLLLRYNDRHIASVEYQQQRPEYDEQGKRQDPWLTVQTLVTYHYNERHQLVRATNAAGESEHYRYSDQHVILERQLAGGASFHWEWEREGKASRCVRQWANYSQMDIRYVWNDNGTATVINADGSEQVYVHDTNARLVSQTDPDGAKLQKIYDEQGRLIAEKDALDAVTEYRYNDAGRLIALIPSEGLPTYYGYFDGQLVSVERGEARWKYDRNRQGDITVQIDPDGNETHYSYDLHGRLLEIRHPDGSRHTLGWNGLGQLLEERLPDGGQRKYRYDALGRQITRQEETGAITHYQWDAANRLAQITLPGGATRAFTYNAYGKVTAERDELGRITRYEYADNLHLVSRRINPDGSQLRYRYDNARLLLTEIENERGEHYHLDYYSNGLIQRETGFDGRTTAYEYDLNGQLLKKTEFGDDGSELVTEYQRDSAGRLLVKTLADGSAVHYRYDALGRLVSVDDGHWPLAYEYDLQDRLITEHQGWGTTRYEYDALGQLSHCRLPDGSKLDYHHQRGGQLGSIDLNGSRLTTHQFIAGREQQRQQGLLLSQYQYDDQGRLQAHSVSQQDRNLFHRHYAYDANGNLAGVNDSRKGNRSFHYDPLDRLISVRGSTPESFAHDPAGNLLAQNDLPVANLANVKGNRLLMQGDRHYDYDAYGNQIRERRGTGQKLVTEYRYDCQHRLIGVSLPGGSTASYKYDAFGRRIEKTVDSHTTEFLWQGERLIAESATNRYRTYIHEPGTFRPLAMLDGEGPLKAQPFYYQLDHLGTPQELTDYSGEIMWSAKYRAYGNLATLDIAEIDNPLRFQGQYFDAETGLHYNRHRYYNPGTGRFLTPDPIKLAGGLNNYQYVPNPTGWVDPLGLSSACPGPDCKLPTNSANATKPDHLPMTQEKFDEIIDMDKADRPPNPGDYLPESYVTAHRELFAKEGGSFVVIESWITGSRFPDFPQRKFVGLTSEMESVVAKYKASGNDWKVLNEELNLGAKNLDGEGIWIVKIKPDDPRFSYEMPNGRENGAYPNEWVPGGATKSGTKEAALIGSEKVTHNSDVGTLLKNFEDWEQLQ>ref|WP_053225210.1MITIIGKMLLTIGDTQPDVKQVLQELEHCIEGYRDRADSWYGGMIDVEQMFRVGDEVVTRDKDDQAPAAMYAQCQRDSTVTLVHAFEAARFIPIGNTQVRLVPVGGGVEIKALIDATGITRVEGCLPNQQYHVTFYPQITSEQLDAFFNSYQEVLSDLSQWLSTQWDTEYLSLWQAHQEADALGRSWIEIKSQWSGLIKSLLAVWDDIVGFLKLISRPQQSYEKFKKFLSEEQLKLLLNASKETLETALLIASDEPLMWSYFSALIAWLKLLPPQVRSEIQARLTTDVLINIVLGIIFSGGIGLAVRLSIKGANTVVESAKATRLLEGFVDLLMRTSKARSTHHATVGKPILLQSEGRLDPGLKADLDIKPQSGLTPAQSIPDATVIARSKPKARTTLEVVEHVDDAPSQSNTPNGKPAEPAKDTQTHGCPVSMVTGEELLTLTDGVLDGALPLEWTRLYRTSAVEVNGDLGFGWSHTLSHRLQREGDELLWTDHENRQTRFPQPSAQRPAIINSLAKAAIYLGDHDCELILAHAGKSSRFYHFNDLRLTAISDAYGNRLKITYQQGRLHRIDNGAGRALLLRYTDARLSAVDYQQYDDSRGFAERSNDHYAKCGIHLERWATLYTQVTYRYNAAGQLISASNALEETEHYRYDAQHVIQERQLAGGAAFYWQWQHTGKQARCIRHWANFGQMDATYEWDDQGSVTVKNRDGSQQVYVHDGNARLVSQIDPDGAEHQKAYDDKGRLIAEKDPLGAVTEYQYNEAGQLTALIPPADEPTFYSYFNGYVRKVQRGLASWKYERNNQGDITCQTDPDGNLTHYKYTAKGQLSGLYYPDGSQHTLTWNPLGQLLDETLPDGTQRRYRYDAQGRTITRQDERGAITQYQWDAANRLSQITLPGGKTRAWSYNAYGKVTAERDERGHITRYEYADNLHLISRRINSDGSQLNYRYDNARLLLTGIENERHEHYQLEYYPNGLIQQEIGFDGRKTAYAYDLKGQLLEKTEYPDAEHPDSAPLVTAYARDPAGRLTNKTLPDGSEITYTYDALGRLTAVDDGTWPLAYEYDLQDRLLSEHQGWATLRYKYDTLGQLSDCRLPDGNRLHYHYQKGGALKGIYLNGKTLTGHRYEAGLETVRQQGYVVSHYQYDEQGRLQDHAVSQLNARGDIEGQPLYGRHYRYDANGNLSLLSDSRKGQKSYAYDPLDRLTEVRGQLTEHFVHDPAGNLLDQSIDGQRHSRYANIKGNRLLMQGDSHFEYDAYGNLRQERRGTGQRLTRDYRYDSQQRLIGITLPDGSQVTYRYDAFGRRISKDHGGTTTQYLWQGERLIAESTQQKGLRGISHYCSYLYEPGTFKPLALLQGEGDAAQVYHYQLDHLGTPQELTDHRGQIAWSAHYRAYGNVLKLDKAEITNPLRFQGQYYDEESGLHYNRHRYYNPNTGRYLTPDPIKLAGGLNSYQYVPNPTGWVDPLGLSSIKGRCPGQADSEPKVEQPEAQKSNSHDDPVTPAPVLHSQETQRVRHYTNRRGSTGIEQDGMITAQDNNRVYVESARKKALDQQSAEEKYQIGRGRGRDYVEFDVPVSQLEEVKNPRYGAVELTIKGNVKLERPEFFRRK>ref|WP_109521272.1MDRVAFVDDQLTTFKDSLKLYREMTESNFAQWVDKGPQFFDRPAIFGMNRTVKLGTEKATVSINDNDFSTVAICPKDGRLLIESKFVSVNDIPVGNITVDITPTAGGASKPVTLDEQGKGVYKGIAGEKYYVRVQSQVTKEQVNDLFGSYDNLTQQLQTWLDGEWKNYRPLWPTETLSTAYLAQLNGAAAGTWDAIKGAFDGIKRIFEILKDPQAFAHELGQSATDLIKLAKETPAAMERLMLLASDQAALFLLLKTTALWLIQLPPSVAVGKFSEQAASVLVTILIDIVLALVLTYAAAGSGIAYLGVRLANYGAKILEAVVGFVKAVFRILKAFIESIDKYKKVAARAAVGAFEKGKVKIGWGRQDHAKIREHEHADDHSKQSTNPDEKSATCAKKTCTNGCPVSMVTGEELLTLTDGKLGGLLPFSWTRLYRTSAVEIDCGLGFGWSHALAQRVDITDDEVIWTNDENLATTFPLPSAQRPAITNNLSEAAIFLGEDPSELILTQAGERAQFYHFRYDSKGATLIALSDNYGNRLHITRDIHGRIKRVDNGAGRALLIRYDRKHIVAIDYQQFVPADNLEDAWSTIQNLATYGYDAQHRLIEAKNAAGEAERYTYNDQNVILERRLAGGASFYWEWEKEGKSARCIHHWASFSQMDAHYVWDDKGTVTVTNADGSEEIYTHDDQARLVAKVEPDGAERLKVYDEKGRLIAEKNPLGAVSEYQYNDAGRLAAVIPPEDAPTEYEYSNGFVSEVRRDESIWKYQRNNQGDITLQIDPHGNETHYSYDRQGRLLEIRHPDGSRHQLGWNNLGQLLEERLPDGGQRKYRYDALGRQITRQEETGAITHYQWDAVDRLTQVTLPGGATRAFTYNPYGRVTAERDELGHVTRYEYADGLHLVSRRINPDGSQLRYRYDNSRLLLTEIENERGEHYHLDYYANGLIQQETGFDGRRTAYEYDLNGQLLKKTEFGDDGSELATEYQRDAAGRLLVKTLADGEEIHYSYDALGRLVNVDDGNWPLAYEYDLQDRLITEHQGWGTTRYEYDQLGQLSHCRLPDGSKLDYRHQSGGRLSSIDLNGSRLTTHQFNVGREQQRQQGLLLSQYQYDEQGRLQAHSVSQQDRNLFQRRYAYDANGNLAGIDDSRKGSRSYHYDPLDRLINVRGSTPESFAHDPAGNLLGQGEQPAANLANVKGNRLLMQGDRHYDYDAYGNLTRERRGTGQKLVTEYRYDCQHRLIGVSLPGGSIASYKYDAFGRRIEKTVDGSATEFLWQGERLIAESADNRYRTYIYEPDSFRPLAMLDGEGPLKATPFYYQLDHLGTPQELTDYSGEIMWSAKYRAYGNLAALDVAEIDNPLRFQGQYFDAETGLHYNRHRYYNPGTGRFLTPDPIKLAGGLNNYQYVPNPTGWVDPLGLDTCPGADGCKPDFDADNATTKPAVDEGQPDVPDAGRKAHQFNSFHEYKVPENLYLKSDKVQFNRANKDLINKLNGDPAFRKNMYSRNPDLKTWVDDPKRNMGSSPTGYTWHHNEKPGVLQLVHRADHGGEHSVYHPTGKGGRDIWGGGREGREGKIKTE>ref|WP_102900571.1MISIAEQRITFVDSQLNAFTLSLNKYREALKSSWNQPFEIGSKVANTPSLLGMERVIRVGNTSKSVNMEDGYFASNVAKCPLQGPLLIHSKFESVFDIPIGDIEVEIIAQEGGAITKIRLDSEGKASWAGGTPGKQYTVRVHQEVSPDQIDELFEAYSKLAGDLEKKLREKWEGPTGYRQQWSSLSLSGTVIAVGKGIFEGGFDAIKGVWEGIKTVLDMLQDPAKFREELGASVDRLENVARQSPHIMKQAMVLASDEAALFLMMNCAFIWLASLPPMKTAAEQAKMTTSFLLTIAIDIVVSIVLTIAAEGTGLIYLGSKLGKYGKVLYNLVVGFIESIFEVIKGFMTYVAKYVRVGLRGVAKRVRNGTTEMRFDGRRNARLAGSEVADDAPRQATTPAGKSTEPTLRTCTDKCPVSMVTGEELLTLTDGQLDGLLPFEWTRLYRTSAVEIDCGLGYGWSHALAHRVEIVGDEVIWTDHENRATVFPLPSEQRPAITNSLSRAAIFLGDDPSELVLAQAGKRPSFYHFRFNSRGATLIAISDSYGNRLHVTRDIHGRIKRLDNGAGRALLLRYDRKHIVAVDYQQFLPADNLEDAWNTVQTLVTYCYDTQHRLIEAKNAAGEAEHYRYNEQHVILERQLAGGAAFYWEWENEGKLSRCTHHWANFSQMEAHYAWDDKGSVTVTNADGSEEVYTHDDQARLISKIDPDGAEHLKAYNDKGQLIAEKDPLGAITEYRYNDNGLMTAVIPPEDEATTYEYINGFVSDVHRGKASWKYQRNRQGDITQQIDPDGNATLYSYDGQGRLLEIRHPDGSRHQLGWNNLGQLLEERLPDGGQRKYRYDALGRQITRQEESGAITQYQWDAANRLAQVTLPGGATRAFTYNAYGNVTAERDELGRITRYEYADNLHLVSRRINPDGSQLRYRYDNSRLLLTEIENERGEQYRLDYFANGLIQQETGFDGRRTAYEYDLNGQLLKKTEFGDDGSELVTEYQRDAAGRLLVKTLADGKEIHYGYDALGRLVNVDDGNWPLAYEYDLQNRLITEHQGWGTTRYEYDSVSQLKHCRLPDGSKLDYRHQPGGQLSSIDLNGSRLTSHQFSAGREQQRQQGLLLSQYQYDEQGRLQAHTVGQRDKSLFQRRYNYDANGNLAGIDDSRKGNRSYHYDPLDRLISVRGATPESFVHDPAGNLLGQNNEGTTNLANVKGNRLLMQGDRHYEYDAYGNLSRERRGTEQKLVTEYRYDCQHRLIGVSLPGSNTVSYTYDAFGRRIEKTVDGYTTEFLWQGERLIAESAENRFRSYIYEPGSFRPLAMLDGEGPRKATPFYYQLDHLGTPQELTDYSGEIMWSAKYRAYGNLAALDVSEIDNPLRFQGQYFDVETGLHYNCHRYYNPGTGRFLTPDPIKLAGGLNSYQYVPNPTGWVDPSGLASGPVTCPQPVAGGKWEFNPKVDLDWRKGAGTPHDQMTTGLNEAFKRTGVPRDEFTPTKWGKSQEGKSFPTEWRVLTGKNKGAEVNIDDPRLVPSSDGPADPHIGYQTPGKRGGGGAVRGHIMLDFVPVSRARIGDPQ>ref|WP_074986248.1MDQIAVIEQELNDFRNSLTRYREMTESWYARAADAGSRATDLPSLLGMERSIRAGSSSKSVSLGDSDFGLVAQCPLSGILEIESKFESAYEVPLGRIQVDVRSRDDNSLQQTLTLGDDGKASFKGQPGKWYSIQVNSAVSAQQVDELFSRYDGLTNQLETWLRGQWADFRPQWQAHGVSSVLSAAGTGILEGSWQAVKDVWEGIKLIFEILQDPMKYAHMLGDKASELAALAKSAPQKMARVMLLASDEAVLYLLLRSATLWLSALPPSQIAGTAAKEISRILVSVLIDILIAIVLTVAVEGTGIAYLAVRLAKYGKRLVDAVIGFVKSVFGILEGVLDYIEKYRVVAARGIASGVQRGKAELVWDARKNSTLREVDARPNVDRQATTPNERPAQACKETCTGGEPVSMVTGEELLTLDDANLDGLLPFVWSRLYRTSAVEVDHGLGYGWSHGLAHRLLIEDGQVTWVDHENRRTRFPLPDRQRPAIHNRLSKAAIFIGADPAELVLAQAGDKPRFYHFRHDGKGGDLVAISDAYGNRLHVGRDLAGRISRLSNGAGRALAFRYEQSRIQAVEYQQQVPADTEEASWRSVQTLASYVYNTAGQLLEAHNALGEVERYRYDEQHVILERHLAGGASFYWAWEGAGKQARCVRQWSSIEPLDFQFEWDDEAGTATLLQSDGGRQVFVHDGNARLVRQVDPDGAVHEKAYNPQGQLVAERDPLGAVTEYHYDEAGHLVALVPPEEEPTSFEYRDGFVSAVERGKANWRYRRNAQGDVIQQTDPDGHSTFFRYDERGRLLQIQHPDGSSHQLLWNGLGQLIEERLPDGGARRYRYDVFGRQITRQDEQGAVTRFEWDALGRLRQVTLPGGASRDYEYNAYGKVTAERDELGRVTRYEYDGGLHLVTRRINPDGSQLRYRYDNARLLLSEIENERGELYRLDYHPSGLISRETGFDGRTCAYAYDLNGHLLEKTEYGEDGSELVTRYQRDASGRLLVRTQPDGSEVHYRYDALGRLVGVDDGEWPLAYEYDLQNRLTREHQGWATLQYGYDALGRLSHCRLPDGSTLDYRHRAGGELSAIDLNGQRLTSHQFSFGRERQRQQGQLLSQYQYDEQGRLLAHQVSQRERALYQRRYSYDAGGNLASVEDSRKGARSYHYDPLDRLVAVRGDLPESFAHDPAGNLLTQNAQQGDPRRVNVQGNRLLMQGDCHYDYDAYGNLLRERRGAGQKLVTQYRYDSQHRLVGVQLPDGRQVSYRYDAFGRRIAKEVDGKTTEFIWQGDRLVAESGAGRYRSYVYEPGSFRPLAMLDGEGPLKAEPFYYQLDHLGTPQELTSYGGEILWSARYRAYGNVARLEVAEIDNPLRFQGQYYDAETGLHYNRHRYYNPNTGRFLTPDPIKLAGGLNHYQYVPNPTGWVDPLGLASTHGDCLNASPPLTTAKIEEITTIPKGARPDPSEYLSSEYIKNHLSQFDNG>ref|WP_005786367.1MIPVIAGFVLAPMDPKAPDVTRVLADFRTCLNTFDAWADSLLSGSALAMEQVFKVGEDVALVAPASSDKPSRTVAQCKAQGGLTLVHLFESTRFVPIGNTQVELQALARDGSPVGAPLHRTIGPSGILEVNDCTRDQQYQITFYPNVSKDHVKALYASYTSVIAGLEADLRKQWDEHFQLQWADFVKAPAYQRSGMQGMAFATGLGKALYNLWDNITELYDLLANFKSNSEKLLKYLSQAELDALLALGKDTLAKGLLVLSDEPLLFIYVAAVVAWIRMLPPPQMYELLGEITGEVLINLLLIWATRGIGVQLRLGAQMLSRVKSGQARALLELLARQVAGPRLDTHVEAAKPVLLSSAATPIRVVPAVPLKAGNQLVSNPVPAVRSKARQTVLVRQEHVDDAPAVASNPKGDAAAPADKTVTNGCPVSMVTGEELLTLTDGALDGVLPFEWTRLYRTSAVEVDCGLGFGWSHALAHGLCVSGDSVVWTDHENRSTTLPLPTAARPAITNSLAEAAIYLGSTPDELVLAQASRFYHFRDGVLTAISDAYDNRLRICRDRSGRIERLDNGAGRSLLLRYELDRIVAVDYQVHRATGREPYVWETEQNVVSYAYDEAGRLVCATNAVGESERYRYDDQHVILERQLAGGASFFWEWERAGKAARCVRHWASFSQMDTRYAWGDDGRVTVHNADGSQEVYVHDDRARLVQRIDPDGAEHFKSYDDKGRLTVEQDPLGAVTAYQYDEAGRLVALFPGDDEPTSYEHDNGFVRVVRRGEAVWKYERNEQGDVTHKTDPDGNSTDYSYNKYGQLTGVWYPDHSCHRLVWNERGQLLEEQLPNGGIKRYRYDDLGRQIAREDEHGAQTVYEWDSVGRLIRLVLPGGTTREFSYNPYGKIIAERDELGHVTRYEYADGLHLISRRINADGTQVKYRYDNVRLLLTEIENEVGETYQLDYHPNGLIQQETGFDGQRTAYVYDLNGNLLEKTEHGDDGSQLITRYERDHAGRLVRKTLPDASVVDYAYDRQGNLLSVEDGHWALAYEYDRQNRLTAEHQGWGTLRYGYDACGQLQKLRLPDNNRLTFNHDKGGHLATVELNGEVLTAHLFKSGREHQRQQGQLLSHYHYDEQNRLHAHAVTQQQNYLYQRQYDYDKSGTLTRLLDTRKGQHDYRYDPLNRLTRADHSQDVQEHFAHDPAGNLLMQDRPGPDIVAGNRLMIQGDRHYDYDAFGNLIRERRGKGQQLVTEYRYDCQHRLISITKPNGETASYRYDPFGRRVSKTVDGKTTEFFWQGDKLIAEHHADCHRSYIYEPSSFRPLALLEGYGPQVTTPYHYQLDHLGTPQELTTPEGEIVWSAHYRAYGQIARLDVGKIDNPLRFQGQYFDQESGLHYNRHRYYNPDIGRYLTPDPVKLAGGVNGYQYVPSPTGWIDPLGLSTNCPGSGKKKPTCSLPAEPDIPDVSRKGAFRQAKRDANVPMTQNPDVMTHPKSGRTTQYIVEKMTDLNDDNILDESGRFINSRVYQFTRADGSKVLIQDHSAGHKFGRPDGIGDQKAHFNLRPIDKPRNGTVNGGKDHYNFRKNR>ref|WP_083365836.1MIPVIARFILAPMDAKAPDVEGVLRDLRKCLNTFDEWAESFWSGSALDVEQVFKVGDEVALVAPASSKKPNRTVATCKAQASLTLVHMFESTRFVPIGNTPVMLQAIAPDGSPMGSPIHRTIGPSGILEVSDCTRDQRYQITFYPDVSKDHIKALYASYQSVIARLERSLRDEWKKTFKPQWDDFANATPFERSAMQGLALSTGIGKAFYNLWDNFTQLYDVLADLKTNSQKLLQYVSQAELDELLKLGKDAIAQGLLVLSDEPLLFIYLSAMVAWIRMLPPPQMYELLGEMTGEVLINLFLIWATRGMGVQLRLGMQVLGHIKSERVRKWLQMLADQLIGPRLDAHVEAARPVLLGSSATPIRVVPDAPLKAGDQVVANAVPAVRSKSQRTVLVQQEHVDDVPASARNPNGDAAASLDKTATNGCPVSMVTGEELLTLTDGTLDGILPFEWTRLYRTSAVEVDVGLGFGWSHALAHRLVVVGDAVVWTDHENRSNTLPLPSTSRPAITNSLAEAAIYLGSSPDELVLAQASRFYHFRDGVLTSISDAYDNRLRISRDFLGRVERVDNGVGRSLFLRYASGRIVAVDYQIHRVVDDGPFVWVTEQTVVSYAYDDLGRLVSATNAVGESEVYRYDEQHVILERGLAGGASFFWEWERSGKAARCVRHWASFSQMDTRYAWDDNGQVTVFNADGSQEVYVHDQRARLVQRVDPDGAEHFKSYDEKGRLTVEQDPLGAITAYQYDEAGRLVAVFPGDDEPTTYEHDNGFVRVVRRGQAVWKYERNDQGDVIRKIDPDGHVTDYTYNKYGQLIGVWYPDHSCQRLVWNERGQLLEEQLHNGGIKRYRYDDLGRQIAREDEHGALTQYQWDSVGRLTRVVLPGDGFKEYSYNPYGKITAERDELGNITRYEYADGLHLISRRINADGSQVNYRYDNARLLLTEIENEVGETYRLQYHSNGLIQQETGFDGQRTAYLYDLNGNLSEKTEHGDDGSQLVTRYERDHAGRLIRKTLPDGNTVDYAYDRQGNLLSVEDGHWSLAYEYDSQNRLTAEHQGWGTLRYGYDACGHLQNLRLPDNNRLTFNHDKGGHLATVELNGSVLTSHLFTAGREQQRAQGNLLSHYQHDHQGRLFNQSINDAEGPLYRRHYDYDKSGNLTRLLDTRKGEHRYHYDPLNRLTRADHTQGEQERFGHDPAGNLLMHNRPGPDIVAGNRLMIQGDHHYDYDAFGNLIRERRGKGHALVTEYRYDCQHRLIGVKTPNGQTASYRYDPFGRRISKTVDGITTEFFWQGDKLIAEHHANRHRSYLYEPDSFRPLALLEGFGPRETQAYHYQLDHLGTPQELTAPDGEIVWSAHYRAYGEISRLDIEKVDNPLRFQGQYFDQESGLHYNRHRYYNPDVGRYLTPDPVKLAGGLNTYQYVPNPTGWVDPLGLNANCPGSSQKKPTCVLPAEPETPDISRRGAFREAKRDAKIPMTQQPDVMTHPKSGRTTQYKKEKMTDLNDDNVLDNNGRTINTRVYQFTRADGSKVLIQDHSAGHTFGRADGVGDQTTHFNLRPINKPRNGHVPETKEHYNFRIK>ref|WP_083374620.1MDSKTRDVESVINDFKRCLNSYDVWAESFFSFSALDIEQVFKVGDEVALVAPIDRSILPSSTVATCKANGTLTLVHMFQSAKFVPIGNTPVVLQRVDPAGGPLGEPIYKTIGPSGILEVTECDRNQQYRINFYPNVSKAHFKALYASYQSVIAPLEGWLRSEWATTFEPLWTNYSEANVLKRYLSLHQAYARGFGEALYSLWDNIKQLFQWIAHPLIHAEKLLHYLSQAELEKLLSLSADTLAKGLLVLSDEPLLFIYLSALVSWMRMLPPPYMNELLGEISVEVLINLLLGFATAGMGVVVRMSTKVLSGIKSRRAREWLEHIAQQFGKSRVDEHAEVAKPILLGGPVTSIKTVPSAQLKAGDQVVSNPVPLVRSKTRQTALVRQEHVDDMPASGKNPNGDAAAPSDKTVTNGCPVSMVTGEELLTLTDGALDGILPFEWTRLYRTSAVEVDCGLGFGWSHSLAQRLVVTGDSVVWTDHENRITEFPLPTASRPAISNSLAEAAIYLGSLPDELVLAQASRFYHFRDGVLTAISDGYDNRLRIFRDLLGRVERLDNGVGRSLLLRYELGRIVAVEYQVHRAEGPGPFVWVTEQNVISYAYDGAGRLVSATNAVGESEVYRYDDQHVILERQLAGGASFFWEWERSGKAARCVRHWASFSQMDTRYAWDDNGRVTVHNADGSQEVYVHDQRARLVQRIDPDGAEHLKSYDDKGQLTVEQDPLGAVTAYQYDDAGRLVALFPGDDEPTSYEHDNGFVRVVRRGQAVWKYERNDQGDITRKTDPDGNVTDYSYNKHGQLIGVWYPDHSCHRLTWNERGQLTEEQLPNGGVKRYRYDDLGRQVAREDEYGALTQYQWDSVGRLVRVVLPGGATREYSYNPYGKITAERDELGRVTRYEYADGLHLISRRINADGTQVKYRYDNVRLLLTAIENEVGETYRLQYHPNGLIQQETGFDGQRTAYVYDLNGNLQEKTEHGDDGSQLVTRYERDHAGRLVRKTLPDGKIVDYTYDRRGNLLGVEDGHWALAYEYDSQNRLTAEHQGWGTLRYGYDACGHLKNLRLPDNNRLTFNHGKGGHLATVELNGDTLTSHLFKAGRERQRQQGQLVSHYQYDEQNRLHAHVVSQQKHDLYQRQYDYDKTGNLSRILDTRKGEHHYQYDPLARLTRVDHSQDVQERFGHDPAGNLLMQDRPGPDIVAGNRLMIQGDHHYDYDAFGNLIRERRGKGHQLVTEYRYDCQHRLIGITQPNGQTASYRYDPFGRRISKTVDSKTTEFFWQGDKLIAEHHADRHRSYLYEPDSFRPLALLEGFGPKDTQPFHYQLDHLGTPQELTDRDGEIVWSAHYRAYGEIARLDINKIDNPLRFQGQYFDPESGLHYNRHRYYNPDIGRYLTPDPVKLAGGINAYQYVPNPTGWVDPLGLSACPGSEGCKSSLGIDDPARQHRFEEGAPPLPDAHHRFPNDPNDLTKVLGVAPKISKTKHDTQRVEWRPNSDTRIRFESHPGDKGPHVPRHHGEHYHIELKPNKLSWNQAEKQKLLMKVKPENYEPGHGTGFLPGERHPGT>gb|ANF85709.1MDAKTVDVETVLRDFRECLNTFDDWAASFWSFSALDVEQVFKVGDEVALVAPINRSIFPGSTVATCQANGTLTLVHMFQSTRFVPIGNTPVVLQRVDPNGGPLGEPIHKTIGPSGILEITECDRNQQYRISFYPNVSTEHVKALYASYQSVIGELEARLRGEWNSTFQAQWKDYTDATALDRRRMVEAAFLSGMGKALYGLWDNLTQLYELLADIKPNSEKLLQYISQAELDELLKLGNDAIANGLLVLSDEPLLFIYVSAMVSWMRMLPPQEMNELMGEITGEVLINLLLIWATAGMGVAVRLGTQVLGHIKSGRTRDLLELLATKLVMPRLEPHAVVVKPLLLSSTATPIKTVPVASLKAGEQLVSNAVPAVRKKTQQTALVRQEHVDDVPAAGKNPNGDAAAPADKTATNGCPVSMVTGEELLTLTDGTLDGVLPFEWTRLYRTSAVDVDCGLGFGWSHALAQRLVVDGDSVVWTDHENRSTALPLPTVARPAITNSLAEAAIYLGALPDELVLAQASRFYHFRDGVLTTISDAYDNRLRISRDRLGRIERLDNGVGRCLFLRYEQGRIAAVDYQVHRAKGHEPFEWVTEQNVVSYAYDEVGRLVSATNAVGESEVYRYDDQHVILERQLAGGASFFWAWERAGKAARCVRHWASFSQMDTRYAWDDNGRVTVQNADGSREVYVHDQRARLVQRIDPDGAEHFKSYDDKGRLTVEQDPLGAVTAYQYDDAGRLVALFPGEDEPTSYEHDNGFVRVVRRGEAVWKYERNDQGDVIRKTDPDGHITDYSYNKYGQLTGIWYPDNSCHRLVWNERGQLLEEQLPNGGIKRYRYDYLGRKVAHEDEQGALTQYQWDSVGRLTRVVLPGGATREYSYNPYGKITAERDERGHVTRYEYADGLHLISRRINADGSQVNYRYDNVRLLLTAIENEVGETYQLDYHPNGLIQQEIGFDGQRTAYAYDLNGNLLEKTEYGDDGSQLVTGYERDHAGRLVRKTLPDGNLVDYAYDRQGNLLSVDDGHWALAYEYDPQNRLTAEHQGWGTLRYGYDACGQLQNLRLPDNNRLTFNHDKGGHLSTVELNGETLTSHLFKTGREHQRQQGQLLSHYHYDEQNRLHAHAVSQQQHTLYQRQYDYDKTGNLTRVLDTRKGEHHYHYDPLARLTRTDHSQGVQERFGHDPAGNLLMQDRPGPDIVAGNRLMIQGDHHYDYDAFGNLIRQRRGKGHQLVTEYRYDCQHRLIGITQPNGKTASYRYDPFGRRISKTVDDITTEFFWQGDKLIAEHHADRHRSYIYEPDSFRPLALLEGFGPKETQPYHYQLDHLGTPQELTAPDGEIVWSAHYRAYGEIARLDIDKVDNPLRFQGQYFDAESGLHYNRHRYYNPDSGRYLTPDPVKLAGGINAYQYVPNPTGWVDPLGLSKCPGNCGPFEDVVDPANSAIVKGSQPALPAPEEIEWTAHGYKHSPSKKMPWKDILATTKSGPAKYKPGINIENLEREAYKNGRTVTNGKTWKVMEYPESIGASEGVSSRWIRIELSAKTIHGHPISEQEFRRLTK>ref|WP_010456574.1MFVPDKVLALNNAIGLLVVAAMDPERPDVEALFADFRLCLNDYDAWAESFWTGKALDIEQVFKVGHDVRLVAPKNSTTPVSSTVAACPAAGPLTLVHMFDAARFVPIGNTPVMLEPLNADGTFGKPVYDEIGPSGILEIRECERSLRYRITFFPNVSAEHVKALYASYQGVISGLEGWLRNEWTTEFEPSWAEFAEAGFIKRYGQLQQADWRGFENSLNGLWDDVKQIYALIADLQANSEKLLEYLTETELETLLEASAESIANLLLVLSDEPLMFIHLAAITSWLRMLPPQFIAEVVAQIRTGILIGFLLARFTGPAGLKLGISAKVLDKVKSERARKWLAAASLRLAELGTQSDLTTHAGVLKPLAVNVRNAPLNPAPAVPLQINLVTAPVLTVQNPVAIARDKSAAMTRLGRQEHRDDVSDQAKNPNGDSADCGPLTCTNGCPVSMVTGEELLTLDDGSLNGLLPFAFTRLYRTSAVEMDCGLGWGWSHSLAHRLELDAEQVVWTDHENRRTTFPLPGLSRPAIHNSLSRSAIYLGNKPEELIVALAGESPRFYHFENGRLTSISDAYNNRLHITRDRQDRIQRVDNGAGRSLLLRYERRHIVTVEYQSFHPADGLVDAWRTEQTLIAYRYDARHRLIEATNAAGESERYDYDDQHVILQRQLAGGASFFWEWERSGKAARCVRHWATFSQMDTRYAWDDDGAVLVKNADGSEEVYVHDDKARLVRRVEPDGAEHLNTYDDHGRLTTERSPSGAITQYRYDEIGRLIALIPPEDEPTSYEYRHGFLHARYRGKAVWKYRRNAQGDVTEAIDPDGHITHYHYDAQGRLLSIRYPDTSRHVFVWDAIGQLVEETLPDGSQRRFSYDVSGRQIARQDEHGAVTQYQWDAVGRLIQTVLPTGASRAFRYNAYGKITAERDELGRVTRFEYDDDLHLVSRRINPDGTQLRYRYDNARLLLTEIENESGERYRLDYTPGGLIRQETGFDGRRTAYAYDLNGHLLEKTEFGDDGSQLFTSYQRDTAGRLLVKNLPDGVNVEYRYDSLGRLVSVDDGHDHPLEFEYDKHNRLITEHQGWGTLRYGYDACGKLNHLRLPDNSQLDYHHAKGGALTAIDLNGTRLTSHTYKADRELQRQQGMLSSRYDYDEQGRLKAHAISQPQHPLYRRDYTYSANGNLEHIADTRHGQRSYQYDPLDRLIRVRHSRDQQPESFAHDPAGNLLMQDRPGPATLKGNRLLMQGDRHFDYDPFGNLIRERRGAAQKLVTEYRYDCQHRLIGVTLPNGNTASYRYDAFGRRICKTVDDHITEYFWQGDNLVAESGREHYRSYVYEPGTFRPLAMLDGKGPHQACPFYYQLDHLGTPQELTDYSGDIIWAAQYTAYGRLTRLNRDTHQVLDQPLRFQGQYYDAETGLHYNRHRYYNPDLGRYLTPDPSKLAGGLNGYQYTRNPTGWVDPLGLNDCPGGDGCKGPSFGDEDPAGKVKVDEGEPGVPSPKPKKRYLYRGDSRESWEIFEAGFEPLGDSMDLYLHALDNRSPPSFFVSTSTSEKEAIKFATGYGYEDGFVYVIKNIRGIDVNKKLGIISPHRGEVEIALPGGVDSRDILGVTPVNADGSYKGYSTPNPRRK>gb|QAY85078.1MFVPDKVLALNNAIGLLMVAAMDPKKPDVEALLADFRLCLNDYDAWAESFWTGKALDIEQVFKVGNEVRLIAPKTATTPVSSTVAACPATGPLTLVHMFDAARFVPIGNTPVMLEPLSSDGTFGEPVHHEIGPSGVLEIPECDRSLRYRITFFPNVSAAHVKALYASYQDVIDDLEGWLRNEWTTEFEASWAEFAEAGFIKRYGQLQQADWRGFENSLNGLWDDVKQIYALITDLQANSQKLLEYLTETELETLLNASAESIANLLLLLSDEPLMFIHLAAITSWLRMLPPQFVAEVVAEIRTGILIGLLLARFSGSAGLTLGISTKVLDKVKSDRARNWLAAASLRLAELGTRSDLTTHAGVLKPLAVHARKASVNPAPTVPLQISAGGTPVLLVNNPVAIARDKSAATTRLTRQEHRDDAPEQAKNPNGDSADCGPLTCTNGCPVSMVTGEELLTLDDGSLNGLLPFAFTRLYRTSAVEIDCGLGWGWSHSLAHRLELDAEHVVWIDHENRRTTFPLPSAERPAIHNSLSRAAIYLGDQPEEWVLALAGEAVRFYHFRSGCLTAISDAYNNRLRITRDGQARIQRIDNGAGRSLLLRYERRHIVAVEYQSFSPADAPENAWRTEQTLATYRYDARQRLIEATNAAGESERYDYDDHHVILQRQLAGGASFFWEWERSGKAARCVRHWASFAQMDTRYTWDDDGAVLVKNIDGSEEVYVHDDKARLVRRVELDGGEHLKAYDDQGRLIAEQDPLGAVTEYRYDEVGRLVALIPPEDEPTSYEYRNGFLHARYRGEAVWKYQRNAQGDVTKATDPAGQVTHYHYDGNGQLLSIRYPDTSRHLFVWNGLGQLVEETLPDGGQRRFSYDALDRQLTRQDEHGAVTKYQWDAVGRLIQTTLPNGASRAFSYNAYGKITAERDELGRVTRYEYLDDLHLVSRRFNADGTELKYRYDNARLLLTEIENESGEKYRLDYTPGGLIRQETGFDGRRTAYAYDLNGHLLEKTEFGDDGSQLFTSYQRDCAGRLLVKNLPDGVSVAYRYDSLGRLVSVDDGHDHPLEFAYDQQDRLITEHQGWGTLRYAHDACGQLNHLRLPDGSKLDYHHAKGGALTAIDLNGARLTTHQFVFGRERQRQQGQLLSDYAYDDQGRLKAHSVSQQHQPLYRRDYAYSANGNIDSIADTRHGQRNYQYDPLNRLIRVRHLRDQPPESFAHDPAGNLLMQDRPGAAKVWGNRLLMQGDRHYDYDAFGNLIRERRGTAQKLVTEYRYDCQHRLVGVTTPDGRCANYRYDAFGRRISKTVDGKTTEFFWQGDHLVAESSREHHRSYVYEPGTFRPLAMLDGKGPRQACPFYYQLDHLGTPQELTDYSGDIIWAAQYTAYGRLTRLNRDTHQVLDQPLRFQGQYFDAETGLHYNRHRYYNPDVGRYLTPDPSGLAGGINGYQYTRNPTGWVDPLGLSDCPGGDGCRKPSFGDEDPAGKTKIGEGEVILPKPSIKTEYLYRGDLKHPDEVFKSGFKSKGESRDLLLHAMDSDDPPSYFISTSPSRRVGIQFGTKFETRKGFLYSLKKIDGIDVNKELKNLVPFGDETEIAIPNKIDTADILGVTPLKKDGSYVGYSTPNPNRK>emb|SDR02002.1MNPAIGRLALTPMDVKTVDVESVFRDFRECLNTFDDWAHSFWSFSALDVEQVFKVGDEVALVAPINRSVIPSSTVATCPANGTLTLVHMFQSTRSVPIGNTPVMLQRVDPNGGPLGEPIHKTIGPSGILEITECDRNQQYRISFYPNVSKDHVKALYASYQSVISGLEARLREEWAGTFQDQWKDYAEAKPLDRRRMLEAAFVSGLGKALFNLWDNVTQLYDLLADLKPNSEKLLAYLSQAELDELLKFGKDTIANGLLVLSDEPLLFIYVSAMVSWMRMLPPQEMNELWGEITGEVLINLLLIRVLGAMGVAARLGAQVLSAIKSKRARELLELLAKQLVEPGLESHVEAVKPLLLSGPATAIKTVPVAPLKAGEHVVSNPVPAVRNKTQKTVLVRQEPVDDMPVSGKNLNGDAAAPSDKTVTNGCPVSMVTGEELLTLTDGTLDGILPFEWTRLYRTSAVEVDGGLGFGWSHSLAHRLVVSGDSVVWTDHENRMTSFPMPTVARPAITNSLAEAAIYLGASPDELVLAQASRFYHFRDGVLVSISDAYDNRLRISRDFLGRIERLDNGAGCALLLRYASGRIVAVDYQVQRAEGRGPYAWITEQTVVSYTYDDAGRLVSATNAVGESEVYRYDEQHVILERGLAGGASFFWEWERAGKAARCVRHWASFSQMDTRYAWGDDGRVTVHNADGRQEVYVHDDRARLVQRIDPDGAQHFKSYDDKGRLTVEQDPLGAVTAYQYDEAGRLVALFPGDDEPTSYEHDNGFVRVVRRGLAVWKYERNDQGDVIRKIDPDGHVTDYSYDKYGQLVGVWYPDHSCQRLVWNERGQLIEEQLPNGGIKRYRYDDLGRQIAREDEHGALTQYQWDGIGRLIRVVLPGGACREYSYNPYGKITAERDELGRVTRYEYADGLHLISRRINADGTQVTYRYDNVRLLLTEIENEVGETYRLAYRPNGLIQQEVGFDGQRTAYAYDLNGNLREKTEYGDDGSQLVTRYERDHAGRLVRKTLPDGNKVDYAYDRQGNLLSVDDGHWALAYEYDSQNRLTAEHQGWGSLRYGYDACGQLQHLRLPDNNRLVFNHAKGGHLATVELNGAPLTSHLFKAGQEHQRQQGHLLSHYHYDDQHRLHAHAITQQQDHLYQRQYNYDKTGNLTRLLDTRKGEHHYHYDPLGRLTRADHSQDLQERFGHTPAGNLLMHDRPGPDIVAGNRLMIQGDRHYDYDAFGNLIRERRGKGHQLITEYRYDCQHRLIGITQPNGQTASYRYDPFGRRISKTVDGITTEFFWQGDKLIAEHHADRHRSYLYEPDSFRPLALLEGFGPKDTKPYHYQLDHLGTPQELTAPDGEIMWSAHYRAYGQISRLDKGKIDNPLRFQGQYFDQESGLHYNRHRYYNPDIGCYLTPDPVKLTGGINAYQYVPNPTGWVDPLGLSTCPGGDGCKPSTSVPTATESVNHGEPALPQLTRVERQARIDELAEANAHRRLSEIEQAFPRAHFLEKHGAQTTLQSQLERVQTGKNPTTGVIERYAGGTMSGQPKIPSAATRYLSHRDQLNAIYRAKLIFRRTNLATSREPTDMGKTIGEGYKKDTLEYGTQRKATVILDTDGNPITAYTDY>emb|SDK58939.1MDPKAPDVKGVLADFRTCLNTFDEWADSLLSGSALTVEQLFKVGEDVALVAPISSEKPSRTVAQCKAQGGLTLVHLFESTQFVPIGNTRVMLQAIAQDGSPVGAPIHHTIGPSGILEVNDCSRDQRYQITFYPNVSKDHVKALYASYGSVIAGLEADLRKQWDERFKPQWADFSQAPAYKRSGLQGIAFASGIGKAFYNLWDNITELYDLLANLKSNSEKLLKYISQADLDELLKLGKDTLAKGLLVLSDEPLLFIYLAAMVAWIRMLPPPQMYELLGEITGEVLINLLLIWATRGIGVQLRLGAQMLSRVKSGQARALLELLAKQATGPRLETHVEVAKPVLLGSAATPVKVVPAVPLKAGEQLVANPVPAVRSKTRQTVLVRQEAVDDVPAVGSNPKGDAAGPADKTATSGCPVSMVTGEELLTLTDGVLDGILPFEWTRLYRTSAVEVDCGLGFGWSHGLAHRLAVSGESVVWTDHENRRTQFPLPTDSRAAITNSLAEAAIYLGSAPDELVLAQASRFYHFRDGVLVSISDAYDNRLRIVRDRSGRIERLDNGCGRSLLLRYELDRIVAVDYQVHRAKGREPYVWETEQNVVSYAYDEDGRLVCATNAVGESERYRYDEQHVILERQLAGGASFFWAWEGSGKAARCVRHWASFSQMDTRYAWGDDGRVTVHNADGSQEVYVHDDRARLVQRIDPDGAQHFKSYDEKGRLTVEQDPLGAVTAYQYDEAGRLVALFPGDDEPTSYEHDNGFVRVVRRGEAVWKYERNDQGDVTRRTDPDGQVTDYSYNKRGQLTGVWYPDHSCHRLVWNERGQLLEEQLPNGAIKRYRYDDLGREIAREDEHGAQALYEWDAVGRLLKLTQPGGATREFSYNPYGKIIAERDELGHVTRYEYADGLHLISRRLNADGTQVKYRYDNARLLLTEIENEVGETYQLTYHPNGLIQQETGFDGQRTAYAYDLNGNLQEKTEHGDDGSQLITRYERDHAGRLVRKTLPDGSIVEYTYDRQGNLLSVEDGHWALAYEYDHQNRLTAEHQGWGTLRYGYDACGQLQKLRLPDNNRLTFNHDKGGHLATVELNGEVLTSHLFRAGREHQRQQGQLLSHYHYDDQNRLHAHAVTQQENYLYQRQYDYDKSGNLTRLLDTRKGQHDYRYDPLTRLTRADHSQDVQERFAHNPAGNLLMQDRPGPDIVAANRLVIQGDRHYDYDAFGNLIRERRGKGQQLVTEYCYDCQHRLIEVKKPNDETASYRYDPFGRRISKTVEGKTTEFFWQGDKLIAEHHADCHRSYIYEPGSFRPLVLLEGYGPQATTPYHYQLDHLGTPQELTDTAGEIVWSAHYRAYGQIARLDVSKIDNPLRFQGQYFDQESGLHYNRHRYYNPDIGRYLTPDPVKLAGGINGYQYVPNPTGWVDPLGLNTCPGRDGCQSTTGPDSPIAKARVDEGQPSAPQPTGDHRRARIEELSETNAKRRILEYEAKYDMHMVGKHGPEVEPSKMSQRSIDGTDPITGSLPLKRKGVPSSQFNSWKLQLQAWTKATSRVERGLPRYTGIDGNKNDIVRLELPGAGRGYRPNKMDPNNPLFNPSMNGAEMKFREDGTPFTLFPIKE>ref|WP_049709322.1MIPVIGKFVLSPMNAKAPDVEGVLRDFRDCLNTFDEWAESFWSGSSLEVEQVFKVGEEVSLVAPASSKKPNSTVAVCKAQGALTLVHMFESTRFVPIGNTPVMLQAIAPDGSPIGAPIHRTIGPSGILEINDCTRDQHYQITFYPNVSKDHVKALYASYQSVIAGLEGRLRDEWKTTVKPQWSSFANAAPLERSAMQAVAFSTGIGKALYNLWDNFTQLYDLLADLKTNSEKLLQYITQAELDELLKLGKDAIAHGLLVLSDEPLLFIYLSAMVAWIRMLPPPQMYELLGEITGEVLINLFLIWATRGMGVQIRLGAQVLGHIKSGRVRQWLEMLADQLVGPRLEAHIEAAKPLLLGGAGTPIKVVPDVPLKAGDQVISNPVPMARDKAQRTVLVRQEPIDDAPAVAKNPAGDATASSDKTATNGCPVSMVTGEELLTLTDGALDGILSFEWTRLYRTSAVEVDCGLGFGWSHSLAHRLAVSGDSVVWTDHENRSTTLPLPSVSRPAITNSLAEAAIYLGSLPDELVLSQGSRFYHFCDGVLTAISDAYDNRLRIFRNYLGQIERLDNGVGRSLFLRYASGRIVAVDYQVERAVDDGPFVWVTEQKVVSYAYDGTGRLVSATNAVGESEVYRYDDQHVILERGLAGGASFFWEWERSGKAARCVRHWASFSQMDTRYVWEDNGQVTVFNADGSQEVYVHDQRARLVQRVDPDGAEHFKSYDENGRLTVEQDPLGAITAYQYDEAGRLVALFPGDDEPTSYEHENGFVRVVRRGLAVWKYERNDQGDVTRGTDPDGHSTDYSYNKYGQLTGIWYPDHSCQRLVWNERGQLLEEQLPNGGIKRYRYDDLGRQVAREDEHGALTQYQWDAAGRLLKLTQPDGSTREYSYNPYGKITAERDELGSVTRYEYADGLHLISRRINADDSQVKYRYDNVRLLLTEIENEAAETYRLQYHSNGLIQQETGFDGQRTAYVYDLNGNLQEKTEHGDDGSQLVTRYERDHAGRLVRKTLPDGNIVDYAYDRHGNLLSVNDGHWALAYEYDPQNRLTAEHQGWGTLRYGYDACGQLEKLRLPDNNRLTFNHDKGGHLSAVELNGAVLTSHLFSAGREQQRAQGKLLSSYQHDDQGRLFNQGIADAEGIVYRRHYDYDKSGNLTRLLDTRKGEHHYHYDPLSRLTRADHSQGEQERFGHDPAGNLLMQNRPGPDIVAGNRLMIQGDRHYDYDAFGNLIRERRGKGHQLVTEYRYDCQHRLIGLTQPNGQTASYRYDPFGRRISKTIDEKTTEFFWQGDKLIAEHHADRHRSYVYEPDSFRPLVLLEGFGPKETKPYHYQLDHLGTPQELTAPNGEIVWSAHYCAYGEITRLDINKIDNPLRFQGQYFDPESGLHYNRHRYYNPDIGRYLTPDPVKLAGGINAYQYVPNPTGWVDPLGLSANCPPKGKYPSACKTWNEPEAPDISRKGAFREAKRDAGIPMSQSPDMVVDPDTGKTKQYAIEKMTDKNEKRILDDNGMPIDTRVYQFTRSNGSKILIQDHSAGHKFGRVDGIGDHSSHFNLRPIDKPRKGYIEGAKKHYPFRK>ref|WP_057704309.1MDVKTVDVEGVFRDFRDCLNTFDDWAESFWSFSALALEQVFKVGDEVALVAPINRSLFPGSTVATCQANGTLTLVHMFQSTRFVPIGNTAVLLQRVDPNGGPLGEPIHKTIGPSGILEITECDRNQQYRVSFYPNVSREHVKALYASYQSVIAGLEGRLRNEWTGTFEAQWKNYTDISPLDRRRLLETAFLSGMGKALYDLWDNVTQLYELLANLKPNSDKLLQYLSHTELDELLALGNDSIAKGLLVLSDEPLLFIHVSALVSWMRMLPPPQMTELWGEITGEVLINLLLIYALGAMGIAVRLGSRVLSGVKSGQARALLELLAKQVSGPRLDTHVEAAKPVLLNSAAVPVKAVPVVPLKAGETLVSNPVPVVRSKTQRTTLVRQESVDDAPAVASNPKGDAAAPADKTVTHGCPVSMVTGEELLTLTDGALDGVLPFAWTRLYRTSAVELDCGLGFGWSHGLAHRLAVSGDSVVWTDHENRSTTLPLPTSARPAITNSLAEAAIYLGSAPDELVLAQASRFYHFRNGVLVSISDAYDNRLRICRDRSGRIERLDNGAGRSLLLRYELDRIVAVDYQVHRAEGRAPYVWETEQNIVSYAYDEAGRLVCATNAVGESERYRYDDQHVIVERQLAGGASFFWEWEGAGKAARCVRHWASFSQMDTRYAWGDDGHVTVHNVDGSQEVYVHDDRARLVQRIDPDGAQHFKSYDAKGRLTVEQDPMGAVTAYQYDDAGRLVTLFPGDDEPTSYEHDNGFVRVVRRGEAVWKYERNEQGDVIRKTDPDGHSTDYSYNKHGQLTGVWYPDHSCHRLVWNERGQLLEEQLPNGGIQRYRYDDMGRQVAREGEHGAQTIYEWDSVGRLIRIVLPGGATREFSYNPYGKIIAERDELGHVTRYEYADGLHLISRRINADGTQVKYRYDNARLLLTEIENEVGETYQLDYHPNGLIQQETGFDGQRTAYAYDLNGNLLEKTEYGDDNSQLITRYERDHAGRLVRKTLPDDSVIAYAYDRQGNLLSVDDGHWALAYEYNRQNRLTAEHQGWGTLRYGYDACGQLQHLRLPDNNRLTFNRDKGGHLATVELNGDVLTSHLFKSGREHQRQQGQLLSHYHYDDQNRLHAHAVTQQQNHLYQRQYDYDKSGTLNRLLDTRKGEHLYRYDPLNRLTRADHSQDVQERFAHDPAGNLLMQDRPGPDIVAGNRLMIQGDRHYDYDAFGNLIRERRGKGQQLVTEYRYDCQHRLISVIMPNGETARYRYDPFGRRVSKTVDGKTTEFFWQGDKLIAEHHADCHRSYLYEPNSFRPLALLEGFGPEETQPYHYQLDHLGTPQELTDTEGEIVWSAHYRAYGQIARLDVGKIDNPLRFQGQYFDRESGLHYNRHRYYNPDIGRYLTPDPVKLAGGINGYRYVPNPTGWVDPLGLTTCPSGDACRSKAEVANPPKTVTVNKGEPSPPTNTSNEYLYRGDDKTPDQIFKNGFKSKGDSNDLYLHALDSNNPPSNFISTSPSKLVGIDFATQYGTRKGFLYTLKKIPGRDVNKELGKLAPFDNEAEIAIQGKIKTGDILGATPMKRDGTYVGYSIPNPNRNIK>ref|WP_083362194.1MIPVIAKLVLTPMDATAPDVESVFRDFRDCLNTFDTWAERFWSGSALELEQVFNVGEDVTLSAPVSSRTPSAVVATCPAQGSLTLVHLFESTRLVPIGNTPVRVQAIAPDGSALGAPLHRTIDASGRLEIPECTRGQPYHITFYPNVSKDHVKALYASYQSLIAGLESRLREEWKDTFQAQWTEFAKAPPLKRSALQGTAFAGGLAKALYNLWDNITQLYDLIANLKANSEQWLRYVSQRELDELLKLGKDAIAHGLLVLSDEPLLFIYLSAMIGWIRLLPPPQMVELVGEITGEVLINLLLIWATRGMGVKIRLGVQVVGHIKSGRVRRWLEMLAEQLAQPGLAQHVEAAKPLLLGSAATPIKAVAPLKVGEALVPNAVPTVRKKTQRTALVHESPVDDVPVSAKNPAGEASASADKTVTNGCPVSMVTGEELLTLTDGILDGILPFEWTRVYRTSAVEIDVGLGFGWSHALAQRLEMSGDSVVWTDHENRSTTLPLPTIARPAITNSLAEAAIFLGDLPDELVLAQASRFYHFRDGVLVAISDAYDNRLRISRDVLGRIERLDNGAGRSLWLRYAQGRIVAVDYQIHRAKGREPYDWVTEQTVMSYAYDDAGRLVSATNAVGECEVYRYDEQHVILERGLAGGASFFWAWERSGKAARCVRHWASFAQMDTRYVWADDGRVTVHNADGSQEVYVHDQRARLVQRMDPDGAQHFKSYDEKGRLTVEQDPLGAVTAYQYDDAGRLVALFPGDDEPTSYEHDNGFVRVVRRGEAVWKYERNEQGDVTRKTDPDGHVTDYSYNKQGQLIGVWYPDHSCQRLVWNERGQLFEEQLPSGGIRRYRYDDLGRQVAREDEHGALTQYQWDSVGRLVRLILPGGACREYSYNAYGKITAERDELGNVTRYEYADGLHLISRRLNADGTQVQYRYDNARLLLTEIENEVGETYRLAYHPNGLIQQEIGFDGQRTAYVYDLNGHLQEKTEHGDDGSQLITRYERDAAGRLVRKTLPDGNKVDYAYDRQGNLLSVEDGHWALAYEYDRQNRLIAEHQGWGTLRYGYDACGQLQRLRLPDNNRVTFNHDKGGHLGTVELNGEVLTSHLFKAGKEQQRQQGQLLNHYHYDDQHRLHAHAITQQENRYYRRQYDYDKSGNLARILDTRKGEHHYAYDPLHRLTRADHSQDLQERFGHKPAGNLLMHDRPGPDIVAGNRLMIQGDRHYDHDAFGNLIRERRGKAHALVTEYRYDCQHRLIAVKKPNGQIAHYRYDPFGRRISKTVDGVTTEFFWQGDKLIAEHHADRHRSYLYEPDSFRPLALLDGFGSDATSPYHYQLDHLGTPQELTAPDGEIVWSAHYRAYGHISRLDVGKIDNPLRFQGQYFDEESGLHYNRHRYYNPDVGRYLTPDPVKLAGGINAYQYVPNPTGWVDPLGLSQCPGGDGCKASTTAENPAENVNHGEPALPQLSRAERQAKIDKLAEANAYRRLDELEKASPGAHFLEKHGAQTSLESQLERVMTAKNPTTGEIERYLRGPHIGEPRPPSAATHFLSHRNQLNAIYRAQLIFKQINLSASKRPMDMGETIGEGYKKENYEYGKTRNARVFLNEDGQPITAYGDFE>ref|WP_072024084.1MDPEAPDVERVLRDLRNCLNTFDAWADSFFSGSALKVEQVFKVGDEVALVAPISSDTPSRTVAQCKAQGGLTLVHLFESTRFVPIGNTPVTLQAIALDGTPMGAPLHRTIGPSGILQVPECTRDQQYQITFYPNVSEEHIRALYASYQSVIAGLEADLRQQWRERFQPQWADFAKAPPFKRSVKQGAAFATGIGQAFYNLWDNITELYDLLANLKSNSEKLLQYLSQAELDELLKLGKDTLAQGLLVLSDEPLLFIYLSAMVSWIRLLPPPEMYELLGEITGEVLINLLLIWATRGMGVQLRLGAQVLSSIKSGRARALLELLARQVVGPRLETHVQTAKPLLLSSAATPIKAVPVAPLQAGDTLVPNAVPMVRNKTQRTALVRQESVDDVPAVASNPKGDAAASADKTVTNGCPVSMVTGEELLTLSDGALDGILPFEWTRLYRTSAVDVDCGLGLGWSHALAHRLSVSGDSVVWTDHENRSTTLPLPTAARPAITNSLAEAAIYLGSAPDELVLAQSSGFYHFHDGVLTAISDAYDNRLRIFRDRLGRIERLDNGAGRSLLLRYEFDRIVAVDYQVHRAKGHEPFAWVTEQNVVSYSYDDAGRLVSATNAVGECEVYRYDEQHVILERQLAGGASFFWEWERAGKAARCVRHWASFSQMDTRYAWADDGSVNVHNADGSQEVYVHDQRARLVQRIDPDGARHFKSYDDKGRLTVEQDPMGAVTAYQYDDAGRVIALFPGEDEPTSYEHDNGFVRVVRRGESVWKYERNEQGDVIRRIDPEGNATDYSYDKHGQLTGVWYPDNSCHRLGWNERGQLLEEQLPNGGIKRYRYDDLGRQVAREDEHGALTQYQWDAVGRLLKLTQSDGTYREFSYNPYGKIITERDELGHVTRYEYADGLHLISRRINADGTQVKYRYDNVRLLLTAIENEVGETYQLDYHPNGLIRQETGFDGQRTAYAYDLNGNLLEKTEHGDDGSQLVTRYERDHASRLVRKTLPDGNTVDYTYDRQGNLLSVEDGHWALAYEYDKQNRLTAEHQGWGTLRYGYDACGQLKNLRLPDNNRVTFNHDKGGHLATVELNDKLLTSHLFHAGREHQRQQGQLLSHYHYDDQNRLHAHAITQQQNHLYQRQYDYDKAGNLTRLLDTRKGEHRYRYDPLQRLTRADHSQDVQERFAHNPAGNLLMQDRPGPDIVAGNRLMIQGDHHYDYDAFGNLIRERRGKGHALVTEYRYDCQHRLIEVTQPNGQKASYRYDPFGRRISKTLEEKTTEFFWQGDKLIAEHHADRHRSYLYEPDSFRPLALLEGFGPEDVKSYHYQLDHLGTPQELTSPDGEIAWSAHYRAYGEISRLDIGKIDNPLRFQGQYYDQESGLHYNRHRYYHPDIGRYLTPDPVKLAGGINAYQYVTNPTGWLDPLGLSQCPGECASVKGAEDPAHNAKSEENEPTPPSPENVEWTAHGYKHSPSKKMPWKEILAATKSGPAKYKPGIDIENLEREAYKNGRTATNGKPWKVMEYPDSIGASEGLPSRWVRIELSARTIHGHPISEQEFRRLTK>ref|WP_114882015.1MLPSDRLLYLNNSIGLLVVAALNPDQPDAEKLFQEFRLCLNDYESWAEQFWTGTALDVDQVFKVGNDVQLSAPVGSRKPISTSVVMCPVAGPLTLVHMFEAARFVPIGDTPVTLEPVISDVGGVLKFGEPQHYTIGPSGILEVDDCDRGQRYRITFFPDVSTAHIQTLYASYQGLIDGLEKWLREEWVGFQPQWAEFSSAGFLERYGQLQQADWRGFETALNGVWDDVKQLFALLADLQANSEKLLQYLSGVELEALLEASSEAIANGLLMLSDEPLLFIHLAAFTSWLKMLPPQYLAEVVAEVRVELLIGFLLMRVSAGMGVPLRLSAKVLGKIKSPRAREWLAASALRLAELTSAPDLARHAAALKPLMINARPAPLRPTPAVPLEIRTADAQVLTVPNPAAIARNKSHGTTRMERHEPRDDASAQAKNPNGDSADCVPRTCTNGCPVSMVTGEELLTLTDGVLDGLLPFEFSRLYRTSAVEIDVGLGFGWSHSLAHRLEIDGASVVWVDHENRRTRFPLPSVERPAIHNSLSRAAIFLGDEPEELIVALAGDAARFYHFRAGRLTAVSDAYGNRLRITRDRLDRVERLDNGAGRSLLLRYERAHLVAVDYQVWREAAWRTEQTLVSYRFDARHRLIEATNAVGESERYDYDDRHVILQRQLTGGASFFWEWERSGKAARCVRHWASFSQMDTRYVWDDAGSVRVQYVDGREEVYVHDDTARLVRQVAADGGEQLKAYDAQGRLIAEQDALGAVTEYRYDDAGRLIALIPPDDTPTSYEYRNGFLHRRSRGEAEWTYRRNAQGDITEAVDPDGHVTHYHHDPQGRLLSIRYPDSGRHVFVWNDLGQLVEESLPNGGVRKFSYDALGRRITAQDEHGAVTRHVWDAVGRLIQATSPTGATRAWSYSAYGQITAERDELGRITRYEYDDDLHLVSRRIKPDGTRLQYRYDHAQLLLTEIENESGEKYRLDYTPTGLIRQETGFDGRRTAYAYDRNGHLLEKTEFGDDGSTLVTGYQRDAAGRLLLKTLPDGVEVSYRYDRLGRLVGVDDGQDHPLAFEYDLQDRLVREHQGWGTLRYTYDACGQLTRMRLPDNSKLDYHYAKGGALTAIDLNGALLTRHVYLNGREQQRQQGLLLSEYAYDEQGRLHSHAVGHQRSALYRRDFAYSANGNLEHIADTRHGQRSYQYDALDRLIRVRHTRDDVPENFAHDPAGNLLLQDRPGPTSIKGNRLLMQGDRHYDYDAFGNLIRERRGRAQQLVTAYRYDSQHRLIGLTRPDGSTASYQYDAFGRRIRKTVDGQTTEFFWQGDHLIAESSKEQHRSFIYEPGTFRPLAMLDGQGPKRACPFYYQLDHLGTPQELTDYSGEIVWSAKYSAYGKVTSLELATEDYLDQPLRFQGQYFDAESGLHYNRHRYYDPDVGRYLTPDPVRLAGGLNQYRYVPNPTGWVDPLGLTSNCPPPNRPGCSVPDRTAGTKVDEGEPKTPGPIQENHYLYRGDEREPWDIFEGGFEPWGESKDLFLHALDNKSPPSWFVSTSISEKEAAKFATGYGYAEGYIYILKNIRGIDVNKELGAMSPHRREKEIAVPGGINNRDIVGATPVNEDGTYVGYSIPNPYRR>emb|SEE78334.1MNAAIGFMVLAAMDFKTRDVEAVLSDFRQCLNTYEQWAESFWSFSALEVEQVFKVGDEVALVAPISSKAPSSTVAMCKASGSLTLVHLFQSTRFVPIGNTPVMLQAVPKDGGPLGEPIYKTIGPSGILEVTDCTRDQSYRITFYPNVCADHVKALYASYQSLIAPLESQLRNDWTSTFEPLWQDYSEANFVQRYRLLDHAYARGFGDALHELWDNIEQLYEWLADLGPNSEKLLQYLTQAELDALLELSQEAIANGLLVLSDEPLLFIYLSAMVSWLRMLPPPYLNELLGQISAEVLINLLLVWVTAGMGVTVRLGAQVLGKVKSGRVRQWLEQLARPFAQTGLGPHAEALKPLLLGSPATQIKTVPSVPLKAGAIEVANPVPLVREKSAPKTALIKHEAVDDAPSSAKNPNGAASASADKTATHGCPVSMVTGEELLTLTDGELQGVLPFAWTRLYRTSAVEVDSGLGFGWSHSLAQRLEVAGESVVWTDHENRSTTFPLPTAARPAISNSLAEAAIYLGAASDELVLAQGATFYHFQGGVLTAISDGYDNRLRISRDVSGRILRLDNDAGRALLLRYERGHIVAVDYQVFRVESEADDAWVTEQNVVSYRYDEQWRLVEATNAVGESERYQYDSLAVITERQLAGGASFFWAWERSGKAARCVRHWASFAQMDTRYVWDDHGRVTLLNADGSQEVYVHDPRARLVQRVDPDGGEHFKSYDAQGRLTVEQDPLGAITAYQYDEAGRLVALFPGDDEPTAYEYEHGFVRVMRRGAAVWKYQRNDQGDITRQTDPDGNVTEYSYDKRGQLLAVWYPDHSCQRLKWNDLGQLTQEQLPNGGVRRYRYDALGRQIAREDEHGALTHYHWDAVGRLLKVVLPGGATRELSYTPYGKVTAERDELGRVTRYEYADGLHLISRRINPDGSQLKYRYDNTRLLLTEIENESGERYQLAYHPNGLIQQETGFDGKRTAYVYDLNGHLQEKTEFGDDDRQLITAYQRDNAGRLIRKTLPDGSTVDYTYDRLGNLLSVDDGHWPLHYEYDRQNRLTAEHQGWGTLRYGYDNCGQLDHLRLPDNNHLTFNHDKGGHLATVELNGRTLTSHLFKAGREQQRQQGQLLSHYHYDDQGRLAQHEHRHGLRRYTYDPAGNLTRLLDTRKGQHDYHYDPLDRLTRADHSQDVQERFAHDPAGNLLMQDRPGPGIVVGNRLLLQGDRHYDYDAFGNLIRERRGRAQQLVTEYRYDCQHRLIGITQPDGSQASYRYDPFGRRISKTVAGKTTEFFWQGDKLIAEHTHGQHRSYLYEPDSFRPLALLDGFGPANTQAFHYQLDHLGTPQELTSADGKIVWSAHYQAYGKITRLDVGTVDNPLRFQGQYYDRESGLHYNRHRYYNPDNGRYLTPDPVKLAGGVNGYLYVPNPTGWVDPLGLACKAGNCPDSKGKANSGVLTAHEKGGGHLIKKHVARTDEELAERFDSEPNIPASSTFQTLEEAETIVSKSLATHHQEIRKFLNGNKSKLLIRDSSTQPVGRSLLKGAKNSVAAYKFLLVLKRSPKMPDGYLLLTGYPEK>emb|SDR40676.1MGFMVLAAMDTKTRDVEAVLADFRQCLNTYDAWAESFWRFSALELEQVFKVGDEVALMAPISSKAPSSTVAMCKASGTLTLVHLFESTRFVPIGNTPVMLQAIPADGGPLGEPIYKTIGPSGILEVTECTRDQLYRITFYPNVSRDHVKALYASYQSILAPLESQLRKDWTDTFEPLWQEYREANFVQRYRLLDQGYANGFGHVLHELWDNIEHLYQWLADLRPNSEKLLQYLAQTELDALLELSQDAIANGLLVLSDEPLLFIYLAAMVSWMRMLPPPYLNELLGQITAEVLINLLLVWVTAGMGVTVRLGAQVLGKVKSGRVRQWLEQMARPFADTRLDRHAEALRPLLLGSPATPIKTVPSVPLKAGAMEVTNPVPLVREKSAPKTTLIKHQAVDDAPSSAKNPNGQASASADNTATHGCPVSMVTGEELLTLTDGELDGVLPFAWTRLYRTSAVEVDCGLGFGWSHSLAQRLCVVGGSVVWTDHENRSTTFPLPSTARPAISNSLAEAAIYLGAASDELVLAQGARFYHFHDGVLTAISDAYDNRLRISRDGLGRILRLDNGAGRALLLRYDRGHIVAVDYQVFRVDSEADDAWVTEQNLVAYRYDEDWRLLEATNAVGESERYQYDSLSVITERQLAGGASFFWAWERSGKAARCVRHWASFAQMDTRYAWDDNGSVTLLHADGSQEVYVHDPQAHLVQRVDPDGGKHFKSYDAQGRLTVKQDPLGAITTYQYDEAGRLVALFPGDDEPTSYEHEHGFVRVMRRGEAVWKYQRNDQGAITRQTDPDGNLTEYSYDKRGQLLAVWYPDHSCQRLTWNDLGQLIGEQLPNGGVRRYRYDVFGRQVAREDEHGALTQYQWDAVGRLIKVVLPGGATRELSYNPYGKITAERDELGRVTRYEYADGLHLISRRINPDSTALRYRYDSTRLLLTEIENEAGETYQLAYHANGLIQQETGFDGQRTAYVYDLNGHLQEKTEFGDDDRQLITTYQRDHAGRLIRKTLPDGSTVDYTYDRLGNLLSVDDGHWPLHYEYDNQNRLTAEHQGWGTLRYGYDHCGQLETLRLPDNNRLTFNHDKGGHLATVDLNGRTLTSHLFQAGREHQRQQGQLLSHYHYDEQGRLAQHEHAHGLRRYTYDKSGNLTRRLDTRQGQHDYHYDPLDRLTRADHSQAPTERFAHDPAGNLLMQDRPGPGIVVGNRLLLQGDRHYDYDAFGNLIRERRGRAQQWVTEYRYDCQHRLTGVTQPDGSQASYRYDPFGRRISKTVAGKTTEFFWQGDKIIAEHTAGQHRSYLYEPDSFRPLALLEGFGPANTKAFHYQLDHLGTPQELTNAQGKIVWSAHYLAYGQIARLDVGIVDNPLRFQGQYFDRESGLHYNRHRYYNPDNGRYLTPDPVKLAGGLNGYLYVPNPTGWVDPLGLNSCPGKEKCEPVTEGQNPSTNTETNEGAPSAPTPATKKQYLYRGDSRHPNEIFKHGFKSRGKSTDLLLHSRDNTNPPSNFVPTSTSRDVGMTFATSFGLEDGFLYTLKKIPGRDVNKELGILSDYKIEKEIAIPDRIDRKDILGATPVNEDGTSANYSLINPYRK>gb|PKA78208.1MIAATSQTIGLLVLAAMDSKTRDVESVLSDFKRCLNSYDTWAESFFSFSALDIEQVFKVGDEVALVAPINRSILPSSTVATCKANGTLTLVHMFQSARFVPIGNTPVVLQRIDPAGGPLGEPIHKTIGPSGILEVTECDRNQQYRVNFYPDVSKAHYKALYASYQSVIASLEGWLRREWTTTFAPLWKNYSEANFLKRYLALHEAYARGFGQALNSLWDDIKQLYEWITHPLVYAEKLLHYLSQAEFEKLLALSTDALAKGLLILSDEPLMFIHLSAFVGWMRLLPPPYMNELLGEISGEVLINLLLVRATGGMGLVTRMSPKVLGGIKSRRAREWLERIAGQFGKVRAETHTELARPILLGGPVADIKVIPDAPLKAGDKVISNAAQAVRNKRVQRTALVRQEHVDDVPASGKNPAGDAAAPADKTVTNGCPVSMVTGEELLTLTDGTLDGILPFEWTRLYRTSAVDIDCGLGFGWSHSLAHRLVVAGDSVVWTDHENRITEFPLPTVSRPAITNSLAEAAIYLGSSPDELVLAQDARFYHFRDGALTAISDAYDNRLQVLRGYSGRVERLDNGIGRSLFLRYKHGRIVAVEYQIQRAEGPGEFVWRTEQTVVSYAYDDAGRLVSATNAAGESEVYRYDDQHVILERQLAGGASFFWAWERSGKAARCVRHWASFSQMDTRYAWGDDGQVTVHNADGSTEVYVHDSRARLVQRIDPDGAEHFKSYDDKGRLTVEQDPLGAVTAYQYDDAGRLVALFPGDDEPTSYEHDNGFVRVVRRGEAAWKYERNDQGDVTRKIDPDGSVTDYSYNKYGQLTGVWYPDHSCHRLVWNEHGQLVEEQLPNGGVRRYRYDDLGRQVGREDEYGALTQYQLDSVGRLVRVVLPGGTTREYSYNPYGKITAERDELGHVTRYEYGDGLHLISRRINSDGTQVKYRYDNTRLLLTEIENEVGETYRLNYHPNGLIQQETGFDGQRTAYVYDLNGNLQEKTEHGDDGSQLVTRYTRDHAGRLVRKTLPDGKTVDYTYDRQGNLLGVNDGHWALAYEYDSQNRLTAEHQGWGTLRYGYDACGQLKNLRLPDNNRLTFHHGKGGDLSTVDLNGTPLTSHLFKAGRERQRQQGQLISHYGYDEQNRLYAHAVTQQRHDVYERHYHYDKTGNLSRILDTRKGERRYQYDPLARLTRVDHTQDGQERFGHDPAGNLLMQDRPGPDIVAGNRLAIQGDHHYDYDAFGNLIRERRGKDHKLVTEYRYDCQHRLIGITRPDGQTASYRYDPFGRRISKTVDGKTTEFFWQGDKLIAEHHADRHRSYIYEPGSFRPLALLDGFGPKATQPFYYQLDHLGTPQELTAPNGEIVWSAHYKAYGKISRLDASKIDNPLRFQGQYFDPESGLHYNRHRYYNPDLGRYLTPDPVKLAGGINAYQYAPNPTGWVDPLGLNSCPGENGCKPDSTLREPTAGINQGEPALPQLTRAQRQVRINELGEANAHRRLTELEASIPGAHFLEKHGAQTSLESQLERVQTAKNPTTREIERYITGPHAGEPRTPSAATHFLSHRDQLNAIYRAQLIFKQINLSASKRPMNMGKTIGEGYKKDGYEYEKTKKARVILNQDGQPITAYGDF>ref|WP_098479655.1MIEVTRQAIGYLVLAAMDSKTRDVESVLSDFGRCLNTYETWAESFFSFSALDIEQVFKVGDEVALVAPINRAILPSSTTAMCQANGTLTLVHMFQSARFVPIGNTPVMVQRIDPEGGPLGEPIHRTIGPSGILEITECDRHQQYRISFYPNVSKDHFKALYASYQSVIAPLDAWLRSEWETIFEPLWKGYAEASFPKRYLALHQAYARGFGEALYSLWDTVKQLFQWLGEPLAYAEQLLHYLSADELEKLLSLGADTLAKGLLVLSDEPLLFIHLSAMVSWMRLLPPPYMNELLGEISADVLINLLLCLATAGMGMAVRISTNVLGGIKSQRARRWLERMAGQFGKSPMDEHVELARPVLLGGPATSIKTVPVAPLKAGEHVVSNPVPAVRNKTQKTALVRQEPVDDVPVSGKNPNGDAAAPSDKTVTNGCPVSMVTGEELLTLTDGTLDGILPFEWTRLYRTSAVEVDGGLGFGWSHSLAHRLVVSGDSVVWTDHENRSIEFPLPSVARPAITNSLAEAAIYLGASPDELVLAQASRFYHFRDGVLVSISDAYDNRLRISRDFLGRIERLDNGAGCALFLRYVSARIVAVDYQVQRAKGYEPYEWVTEQNVVSYAYDDAGRLVSATNAVGESEVYCYDDQHVILERGLAGGASFFWEWERSGKAARCVRHWASFSQMDTRYAWGDDGRVTLHNADGSQEVYAHDDRARLVQRIDPDGAQHFKSYDAKGRLTVEQDPLGSVTAYQYDEAGRLVALFPGDDEPTSYEHDNGFVRVVRRGLAVWKYERNDQGDVIRKTDPDGNITDYSYDKYGQLVGVWYPDHSCQRLVWNERGQLLEEQLPNGGIKRYRYDDLGRPVAREDEHGALTQYQWDSVGRLVRVVLPGGACREYSYNPYGKITAERDELGHVIRYEYADGLHLISRRINADGTQVNYRYDNVRLLLTEIENEVGETYRLDYHPNGLILQETGFDGQRTAYVYDLNGNLQEKTEHGDDGSQLVTRYERDHAGRLVRKTLPDASVVEYAYDRQGNLLSVDDGHWALAYEYDQQNRLTAEHQGWGTLRYGYDACGQLQHLRLPDNNRLVFNHSKGGHLATIELNGALLTSHLFKAGQEHQRQQGQLLSHYHYDDQHRLHAHAITQQQDHIYQRQYDYDKTGNLTRLLDTRKGEHHYHYDPLGRLTRADHSQDLQERFGHTPAGNLLMQDRPGPDIVAGNRLMIQGDRHYDYDAFGNLIRERRGKGHQLVTEYRYDCQHRLIGITQPNGQTASYRYDPFGRRISKTVDGITTEFFWQGDRLIAEHHADSHRSYLYEPDSFRPLALLEGFGPEETKPYHYQLDHLGTPQELTDPDGEIVWSAHYRAYGQISRLEKGTIDNPLRFQGQYFDQESGLHYNRHRYYNPDIGCYLTPDPVKLAGGINAYQYVPNPTGWVDPLGLSCKIGNCPDSKNDLTKKAPPGVLTTHEKAGGHLIEKHVGRTDEQLAARFESEPHIPASSTFKTLEEAEAIVSKSLTAHQQEITTFLSGNKEKLLIKGSSSQPVGVSMLNGATKSVPVYKFLLVIKRAPKMPDGYLLLTGYPEK>gb|AMW84563.1MAAMDPKTRDVDGVLNDFTHCLNTYDAWAESFWSFSALDVEQVFKVGDEVSLVAPITRSLYPSSTVATCKANAPLTLVHMFQSTRFVPIGNTPVMLQEVAQDGGPLGDPIHKTIGPSGILEVTECTVDRQYRVSFYPNVSREHIKALYASYQSEIGSLEGRLRGEWTNTFKPIWESYSKADFVDRYTSLQLAYAQGFGNVLYGLWDGIAQLYQWLADIRPNSEKLLQYLSQAELEALLKLSNEAIAKGLLILSDEPLLFIYLSAMVSWMRMLPPHYMLELLGEITTEVLLNLLLLWATGGMGVAVRLGAQVLGKVKSERAREWLEQIARLGGTARMDGHAEVLKPVSLQSHAVPVKAAAVPLKVGPDQVSNPAPMVREPTARTTLVKQEPVDDVPASAKNPGGDAAAPADKTATNGCPVSMVTGEELLTLTDGSLEGVLPFAWTRLYRSSAVELDCGLGFGWSHSLAQRLVVSGESVVWTDHENRSTTFPLPSAARPAITNSLAEAAIYLGGLPDELVLAQASLFYHFRDGVLTSISDAYDNRLRISRDYSGRIQRIDNHVGQGLLLRYDRGHIVAVDYQVQREIGWVTEQNVVAYKYDDAWRLIEATNAVGESERYRYDDQQVILERELAGGASFFWEWERSGKAARCVRHWASFSQMDTRYVWDDNGTVTVHNADGSQEVYVHDQRARLVQRIDPDGAQHFKSYDEKGRLTVEQDPLGAVTAYQYDEAGRLIALFPGDDAPTSYEHDNGFVRVVRRGDAVWKYQRNDQGDITRKTDPDGNYTEYTYTRHGKLSGVWYPDNSSQRLIWDERGQLIEEKLANGGVRHYRYDDLGRQVACEDEHGALTQYQWDAMGRLLKVIQPDGATREFSYNPYGKITAERDELGRVTRYEYADGLHLISRRINPDGTQLKYRYDNVRLLLTEIENEVGETYRLEYHANGLIQQETGFDGRRTAYAYDLAGHLLEKTEYGDDGSQLVTGYARDAAGRLVRKTLPDGSSVNYTYDRLGNLLSVDDGHWPLHYEYDLQNRLTAEHQGWGTLRYGYDECGQLNYLRLPDNNRLAFHHDKGGDLGTVELNSNLLTSHLFKSGREHQRQQGQLLSHYHYDDQGRLHAHAITQQEQRHQRRQYDYDKRGNLTRILDTRKGQHDYHYDPLDRLTRANHSHDLQERFVHDPAGNLLMQDRVGPSIVKGNRLLMQGDRHYDYDAFGNLLRERRGRAQQLVTEYRYDCQHRLIAVTQPDGTQASYRYDPFDRRVAKVVNGQTTEFFWQGDALIAEHSADHHQSYLYEPNSFRPLVLLKGYGPEDVKPFHYQLDHLGTPQELTAPDGETVWSAHYRAYGQIAKLDVNTLTNPLRFQGQYFDPESGLHYNRHRYYNPDIGRYLTPDPVKLAGGLNGYQYVPSPTGWVDPLGLSCKATACPGQIVADGPYSEIVPGGGLAAHEAAGGHLMQKHIGRTDQQLATRLKNEPHVPTASTFPDRATAESAISSVIAGNETKINSFVKGKDKKIVITQKSPQPVGTSLKRGAKTTVPGREIYLILYKDKSMPDGYRIQTGYPNP>ref|WP_081353090.1MLPPDPLLKLNNSIGLLVVAALNPDQPDVEKLFQEFRLCLNNYDAWAEQFWTGTALGVEQVFQVGNDVRLSAPVGSRRPISSSVVMCPATGPLTLVHMFEAARFVPIGNTPVILEPVISDVDGVLGFGEPLHHTIGPTGILEVSDCDRGQRYRITFFPDVSTAHIQALYASYQGLVDGLEGWLREEWKGFQPQWTAFSSAGFTERYGQLQRADWHGFESALNGVWDDVKQLFALLADLQANSEKLLQYLSSAELDALLEASSEAIANGLLMLSDEPLLFIYLAAFTSWLKMLPPQYLAEVVAEVRAELLVSFLLMCVSGGMGVPLRLSTKVLGKIKSPRAREWLAASALRLAELTSAPDLTRHASALKPLMVNAGPAPLRPTPAIPLEVRTEDALVLTVPNPAAIARDKSHGMTRMERHEPRDDASDQAKNPNGDSADCAPRTCTNGCPVSMVTGEELLTLTDGVLDGLLPFEFSRLYRSSAAEIDVGLGFGWSHSLAHRLEVDGASVVWVDHENRRTRFPLPNVERPAIHNSLSRAAIFLGDEPEELIVALAGDAARFYHFRVGRLTAVSDAYGNRLRITRDRLDRVERLDNGAGRSLLLRYERAHLVAVDYQVFRESAWRTEQTLVSYRFDARHRLIEASNAVGETERYDYDDQHVILQRQLAGGASFFWEWERAGKAARCVRHWASFSQMDTRYVWDDAGSVTVQYVDGTEETYVHDVTARLVRQVAADGGEQLKAYDAQGRLVAEQDALGAVTEYRYDDAGRLIALIPPDDAPTSYEYRNGFLHSRSRGDAVWTYRRNAQGDITEAVDPDGHVTHYHYDAQGRLLSIRYPDSGRHVFMWNDLGHLVEESLPDGGVRKFSYDALGRRTTVQDEHGAVTRHAWDAVGRLIQTTSPNGATRAWSYSAYGQITAERDELGRITRYEYDDDLHLVSRRINPDGTQLRYRYDHAQLLLTEIENESGEKYRLDYTPTGLIRQETGFDGRRTAYVYDRNGHLLEKTEYGDDGSTLVTTYQRDSAGRLLLKTLPDGVEVSYRYDRLGRLVGVDDGQDHPLAFEYDLQDRLVREHQGWGTLRYTYDACGQLTRMRLPDNSKLDYHYAKGGALTAIDLNGALLTRHVYQHGREQQRQQGLLLSEYAYDEQGRLKSHAVGHQRSALYRRDFAYSANGNLEHIADTRHGQRSYTYDALDRLIRVRHTRDDVPENFAHDPAGNLLMQDRPGPTQIKGNRLQMQGDRHYDYDAFGNLIRERRGRAQTLVTEYRYDSQHRLIGLTRPDGKTATYQYDAFGRRIRKTVDGQSTEFFWQGDHLIAESSKEQHRSFIYEPGTFRPLAMLDGKGPKRACPFYYQLDHLGTPQELTDYSGDIVWSAKYSAYGKVTSLELATEDYLNQPLRFQGQYFDEESGLHYNRHRYYDPDLGRYLTPDPVKLAGGLNQYQYVPNPTGWVDPLGLTSNCPPKNGKSVACSDFGEVELPDVSRRGAFRQAKADAGIPKGQQPDVQFDEVSGKPGQFIYINMTDINGKSILNDSGIPIRTRQYMYTRQDGFKLILQDHSAGHKFGDARKIGDQGPHFNIRPAANPRTGKVKNTDEHYPFRK>ref|WP_083351558.1MTPVIARFILAPMDAKAPDVEGVLRDLRECLNTFDEWAESFWSGSALEVEQVFKVGDEVSLVAPASSKKPNRTVATCKAQGALTLVHMFESTRFVPIGNTPVMLQAIAPDGSPMGAPIHHTIGPSGMLEVSDCTRDQRYQITLYPNVSKDHIKALYASYQSVIAELEMGLRDEWAKTFKPQWSDFANAISLERSAMQGMAFSSGMAKALYNLWDNFTQLYDLLADLKSNSQKLLQYVSQAELDELLKLGKDTIAQGLLVLSDEPLMFIYLSALVAWVRMLPPPQMYELLGEMTGEVLINLFLIWATRGMGVQLRLGMQVLGHIKSERVRKWLQMLADQMVGPRLEGHVEAARPLLLGSAETPIRVIPDAPLKAADQVVSNAVPVVRNKSQRTALVRQETVDDVPVSARNPNGDAAASSDKTATNGCPVSMVTGEELLTLTDGTLDGILPFEWTRLYRTSAVDVDCGLGFGWSHALAHRLSVSGDSVLWTDHENRSTTLPLPSTSRPAITNSLAEAAIYLGSSPDELVLAQASRFYHFRDGVLTAISDAYDNRLRITRNFLGRVERVDNGVGRSLWLRYASGRIVAVDYQIHRVVDDGPFVWVTEQTVVSYAYDDLGRLVSATNAVGESEVYRYDEQHVILERGLAGGASFFWEWERPGKAARCIRHWASFSQMDTRYAWDDNGQVTVFNADGSQEVYVHDQRARLVQRVDPDGAEHFNSYDDRGRLTVEQDPLGAITAYQYDEAGRLVAVFPGDDEPTTYEHDNGFVRVVRRGQAVWKYERNDQGDVTRKTDPDGHVTDYTYNKYGQLIGVWYPDHSCQRLVWNERGQLLEEQLPNGGIKRYRYDDLGRQIAREDEHGALTQYQWNDVGRLVQVVLPSGDTRKYTYNAYGKITAERDEQGHVTRYEYADGLHLISRRINADGTQVKYRYDNARLLLTEIENEVGETYRLQYHNNGLIQQEIGFDGQRTAYVYDLNGNLQEKTEHGDDGSQLVTRYQRDHAGRLIRKTLPDGNTVDYTYDRQGNLLGVEDGHWSLAYEYDSQNRLTAEHQGWGTLRYGYDACGHLQNLRLPDNNRLTFTHDKGGHLATVELNGAVLTSHLFKAGREQQRQQGQLLSHYQHDHQGRLFNQSIVDAEGPLYRRHYDYDKTGNLTRLLDTRKGEHRYYYDPLNRLTRADHTQAEQERFGHDPAGNLLMQNRPGPDIVAGNRLMIQGDHHYDYDAFGNLIRERRGKGHQLVTEYRYDCQHRLIGIKKTNGQTASYRYDPFGRRISKTVDGITTEFFWQGDKLIAEHHADRHRSYLYEPDSFRPLALLEGFGPKETKPYHYQLDHLGTPQELTAQDGEIVWSAHYRAYGEITRLDIGKVDNPLRFQGQYFDQESGLHYNRHRYYNPDVGRYLTPDPVKLAGGINTYQYVPNPMGWVDPLGLNANCTGSVKKNPTCAIHVEPDVPDVSRKGAFREAKRDANIPMAQQPDVITAPRSGAKRQYSIVKMSDINGKAILDSSGKPVHTRVYQYTRADGSKVLIQDHSAGHKFGTPNGVGDQGAHFNLRPFDTPRTGSVPGTKDHYIFKDKK
