# Supplementary material for: Intramolecular chaperone-mediated secretion of an Rhs effector toxin by a type VI secretion system
Source: Nat Commun. 2020 Apr 20;11:1865. doi: 10.1038/s41467-020-15774-z (PMC7170923; doi:10.1038/s41467-020-15774-z)
Supplement: Supplementary file 5 — Supplementary Data 2 [file 41467_2020_15774_MOESM5_ESM.zip › 228713_2_data_set_4503146_q7ndn1.rtf]

>TseI MSNAGQAVFNEVIADYRNVLNAYRKDAESFFLGDMLSMDMEQTIKVGDKTITASSSSKKAQSVVTQCPLSGTLRLVHLFESVRFIPIGNTPYKVEAGKLVRGRFVSEAVAKEGTLDGKGIAEVGKLTPGKSYRVTFYPNVKKSDLDTLFTSYVPVQKDLEAWLTKEWNSEHKAAWARYSGTGGGFGSHALAAASGIGKALVGVWDDLKTIYNLLADPVGNAKKLAAFGVDAAAMAKAGASQMEAAMLVLQDEALLYLYAYALVCWVKMLPPDQQTEFGAQVISSVLIDVIIGVVLTGGAGLAARYGAKAASMAKNSDRVMRLVTSLINLSKKHNLAQHAQQAKKVLVAGAAPLSPAKKADLKLVDGNAKTLVEKGTAAKRRFQGHTEIKQVNKTPDASKQAVTPANRPHQPEANTCKNNCPVSMVTGEELLALQDGELPGLLPFVFGRLYRTSAVAQSCGMGSGWTHALAHRLERHGDTLTWWDEESLATELPLPSAARPMVTNLLSEAAVYLGDEADEVILAKAGSPFLHFTWHGDRGRLTGFSDPYGNRLTVRSDRQGRPAWVENEGGLALRLAYEGDHIMALELQRFDGIAWQPQATLQRYHYDDHGHLVAAENGAGECERYRYRDDGVIVERRLAGGAGFFWEWEGEGKAARAIRHWSDVAGFDVTYGWDDDKGEVTVLNADGSQEVYQHDSQARLVRQQDPDGAITEFAYNDKGQKVLARDPLGAETHYHYDEEGMLALEVAPDGSQTAYDYWDGRVRKVVQGEREWRFEHNPQGDLIARRDPLGNETRYGYTPQGKLCSVVHPDSTRTELVWNRLGQLIEEKGVDGGISRWRYDERGRQIVRQDPRGAITRYEWDATDRLSAVHLPGGGVRRYEYNAYGKVTAQWDELGRETRFEYHPGLHLVSRRINPDGSELKYRYDNAKLLLSEIENEHGEQHRIHYHPNGLVARETGFDGRTTAYRYDLNGRLSEKVEFGKQETELVTRYERDLMGRLLKKSLPDGREIAFEYDGHGQLIRVDDGAWPLAFDYDGAGHLVAEHQGWASSYFRYDAMGRLSHWQLPDGNRLGYHHQHGALGGIDLNGAELTRHQVVGGLELRRQQGALTQQYEYDEQGRLSALRLQRGKQVARERRYGYDRTGNLLRIDDSVQGEQHYRYDPLDRLLEVRGELTERFLHDPAGNLLGESSGGQFDGARTQGNRLLFSGDRHFEYDEFGRLARERRGKGQQLVTRYHYDCQHQLVRAELPDGNTACYDYDAFGRRIRKTVRGAKGEQVTEFLWQANNLIAESCYLLGDDMHRTDEQYRSFIYEPGSFKPLAQLEGEGQEAEVFHYQLDHLGTPLALTRVSGVTAWQVRYRAYGNVWREEIAEVATPLRFQGQYFDAETGLHYNRHRYYQPGTGRFITPDPIGLAGGLNNYQYAPNPTGWVDPLGLSNVPGQCPDASPSRRESFRAAKEAAGIPRSAQYKTHKYVYDKEYENRTVYEFDVHGSKKYIVLHDDDKFGRGPHFHGADDSKGSPMLPGKYKQYPGHFPEIKIGFRKGKIKK>PSPTO_5438 MDRVADIEAQLDSFKDSLTLYREQTKTWYAELADKGSRATDMPSLLGMERVIKAGGSSKAVSMNDDDFSYVAKCPLVGPLLIESKFESLYDIPVGDIQVDVIAVEGGAKTTVMLDKQGKGSFQGQPGKAYKIHVHDKVTPAQIDALFKSYDGLSADLEGFLRKEWAGFKPQWSNQSASATAMAIGAGILEGGWEAIKGVWDGITKALDILQNPKKFAEELGAGADELVKLAKEAPDVMKKAMLLASDEAALFLMVRCASIWVAGLPPTQAAGQTAKMTSAATIGILIDIVISVVLTLAAEGAGLFYLAARLAKYGKIILNAVTGFVKAVFNIIKGFMGYVSKYVAVAARGAVSATNQGVAQLRFDGKRNTTVRTSKRLDDAASQSRTHDGKSAAAAQDTCVNGCPVSMVTGEELLTLTDGQLDGLLPFEWTRLYRTSAVETDSRLGYGWSHALSHRLQLDDEGVLWIDNENRQTRFPMPTEHRPAITNSLAKAAIYLGNTPGELILTQAGKQPRFYHFRAGRLITISDAYDNQLQITYDLVDRVQRIDNNAGRSLLLRYEDRHIVAVDYQQQRPDYTDQGERQDPWATVQTLVTYQYNVKGQLVSATTAAGESEHYRYNEQNVIQERQLAGGASFFWEWENEGKSSRSIHHWANFSQMDSRYIWDDAGQVTVLNADGSEQVYVHDENARLISETTPDGAETQKAYDEKGRLIAEKDPLGAITEYRYDEDGLLIALIPPEDEPVAYEYHRGFVSDVRRGKATWKYQRNEQGDITVQTDPDGNETHYSYDLRGRLLEIRHPDGSRHQLGWNGLGQLLEERLPDGGQRKYRYDALGRQITRQEETGAITHYQWDAANRLAQATLPGGATRAFSYNAYGKVTAERDELGRITRYEYADNLHLVSRRINPDGSQLRYRYDNARLLLTEIENERGEHYHLDYYSNGLIQRETGFDGRSTAYEYDLNGQLLKKTEFGDDGSELSTEYQRDAAGRLLVKTLADGSAIHYRYDALGRLVSVDDGHWPLAYEYDLQDRLITEHQGWGTLRYEYDTVGQLKHCRLPDGSKLDYHHQRGGQLGSIDLNGSRLTTHQFSAGREQQRQQGLLLSQYQYDEQGRLQAHSVSQQDRHLFHRHYAYDANGNLAGVNDSRKGNRSYHYDPLDRLINVRGTTPESFAHDPAGNLLGQGDQPAANLANVKGNRLLMQGDRHYDYDAYGNQIRERRGTGQKLVTEYRYDCQHRLIGVSLPGGSVASYKYDAFGRRIEKTIDGHITEFLWQGERLIAESADNRYRSYIYEPGTFRPLAMLDGEGPLKAAPFYYQLDHLGTPQELTDYSGEIMWSAKYRAYGNLATLDIAEIENPLRFQGQYFDAETGLHYNRHRYYNPGTGRFLTPDPIKLAGGLNNYQYVPNPTGWVDPLGLSGECPDSEKNKIPVEEASSPSNSYDNSEELRPPESNGRRYAAGKHYVNPQDINFSQRGVHGNEYEAKMSQGDWDWSRPGSALNVMEVDGQLVSYDNRRLRAARSVGLNSIPIQIVLAEDIMPGSKKTWEKAFKLRREDRRNLLDGEPVPPKGLNTLPKINE>PSF113_5204MFAPDKLLALNNTIGLLVVAAMDPEQPDVDAVFQDFRTCLNDYDAWAESFWTGWALDLEQVFKVGNEVSLAAPKNLDTPISATVVACPAEGPLTLVHMFQAARFVPIGDTPVMLESVIDGAPGQETFGEPIHHVIGPSGILEVRECWRGQRYRITFFPHVTADHVKALYASYQGVIGELEGWLKAEWEQFQPLWKEFADAGFLERYGVLQQADWHGFEKALHQLWDDVKQVFGLLADLQANSEKLLEYLTQAELEQLLKASSEAIAKGLMILSDEPLLFVHMAAFASWLRMLPPQYLAEVVAELRASVLINFLLMRLTGGLGLGLRMSGNVLGRVRSERAREWLLASSLRLGQLAPDRLNQHADVLKPLVISQRAPLKPTPVGPLHITPEHSAPLSVSNPAAVAREKSQGATRLSKYEPHDDAPTQSKNPNGDPADTASQTQTSGCPVSMVTGEELLTLDDGTLDGRLPFVFTRLYRTSAADLDVGLGRGWSHALAHRLELDDEQVTWVDQENRRTIFPLPSAQRPAIHNSLARAAIYLGTEADELIVAQPGENAPFLHFRDGHLVALSDRYDNRLTLQRNIHGDICRLDNGAGRALRLRYEQRHLIAVDYQSFHPAITLDEAWRTEQTLVSYRYDGRFRLIEATNAAGESERYDYDDQHVILQRQLAGGASFYWEWQGLGPASRCVRHWASFAQMDSRYTWGEDGSVTVRHLDGSQEVYVHDDRARLVRKVEPDGGEHLKAYDEQGRLIAEQDPLGALTEYRYDDVGRLVALLPPDETPTSYEYRNGFLHTRARGKAVWSYRRNARGDVIVLIDPDGRRTEYAYDVHGQLLATYDPDGAEHRFTWSRLGQLTEEILPDGSRRCFSYDALGRLLSRQDEHGALTHYQWDAVGRLLQVTLPNGATRTWRYNAYGKVTAECDEQGRVTRYEYADDLHLVSRRLNPDGSELKYRYDSARLLLTEIENESGEKYQLDYTPNGLIRQQVGFDGQRTAYAYDLNGHLLEKTEHGEDGSQRLTQYQRDAAGRLRVKTLPDGQAIEYRYDELGRLVHVNDGSNHPLAFEYDAQDRLVCEHQGWGTLRYRYDACGRLNHLRLPDDSQLDYHHAPGGALTAIDLNGSRLTEHRFVGGRERQRQQGRLLSDYTYDEQGRLKAQTVWQNQQQQLFWRDYAYSAKGNLQTLSDNRNRRSYQYDPLDRLTRIDFSHSEPPEHFCHDPAGNLLMQDRPGPTTLKGNRLLKEGDRHYDYDAFGNLIRERRGQALVSAYRYDSQHRLIGVTTADGRETSYRYDAFGRRISKAVDGLTTEFFWQGDQVVAENSPRHHRSYVYEPGTFRPLAMLDGEGPEKATPFYYHLDHLGTPQELTSHSGQIVWSARYSGYGQLTELQHGGGEQLEQPLRFQGQYFDPESGLHYNRHRYYNPETGRYLTPDPSKLVGGLNGYRYTLNPTGWVDPLGLVDCPGKGGCRPGVGEQDPAGKVAVDQTEPKLPTPKKEEDFLYRGDERNPEDVFEHGFKSKGKSKNLFLHSMDSDSPPSYYISTSYSRDVGKRFATGEYTKIGYLYTLQKIPGHDLQKELGGAYLFEAEKEIAIPGRIKNEDVLGATLILDNGKEFGYSIPNPNRRIKK>VP1517 MIPQFVIPLTNCLGQSYHFSSQPIPKGEHKKFDSEQSAKAFLDDFVPLRSSRVEELYHLLGQFPPNVPDEELTPELYAPVFAKALVNGSLYVASFPKTKKNATISSEPTPVPKQVKAKSKQNKAHTSSKTQAKNSASAKPLQTGSECHEKAGDPVSLVTGEEILTLNDVELPNGFVWSRTYRSSKASRNQGLGYGWRHAFQFELKEVTDEKHNVTSWEFISDSADEIEFEPVEHGSTSYQVYVGASCHFLNPNIRIVTLSSGDQYRFELVEDIWLLKQVRNGIFSTFQLRYSRNHRLIEVAHNKRPVLECQYDKQGRLVELLNAKTEQVLTTYIYDEQDDLVGATNDLGLTERYEYQDQHLIAKRVRPTGFTHYFEWSGEGSSAKCIRNFGDSGIYDYRFHYEGAKSSYSDSLDNEWTFIHDEQGHLLEKSSPTGRTWQWHYDHLGRKEKAVFPDNSTTQYQYNQQGQLISKLHSSGAQIQYGYDSLGKLVKTVSPDGDLEKAYYNSLGQRVWDIDALGCVTEYEYDKHGQVVKRESEDGKKSRWWWDKQQRLVAHEVDGTLLRYSYGATDLVNGIAYPDGCVAQISYDDYGRRTSIRYFNDEDKVGYSEEYAYDEFSRVAQIQTPEGVTSYQWGALAQQEAVIFPDGSHISYEYDQQRNLTKLVRSDGLAFEFWYDSEGLLSGTVGFDGLHSQFKYDSMGRIIRKDVADRTVLYSYDDAGFLQHIKAGNGKNIVENHFNYTLGGRLTLASNRHQTLQYQYSSFGHLTKRIQGQFEIGEEFNRVGQRVSQTLPDKTSFNFSYDTNGRLSEIRFSDDSLPKIEFQYDVMGRLSVTETESFRESKLYDGVGRLVEQQWSGREKKYIYNAQNRISSILDNTAGATHYQYDTLGYVTKVSEAGSTSTFESDSFGNPALADSKVMSDRIEAYAGVRYKYDQQGNQVKREGDGTVQKRVFDALSQLVEVHGDSSISHYEYDALGRRTKKITQNGITEFLWEGERLLGERTADGFRWYLYQPETYIPLAVLENGSIYLYECDQVGKPERLKDSAGNIVWSASYDVHGFASIDVEEVRNPLRFQGQYFDQETNLHYNLARYYDPKLGRFIQQDPISIAGGINHYQYAVNPIQWIDPTGFLCEEGLKRLQQMLAEYQAQSDVPQEVCDQILEAAKESSVGEDGVRSQVKIRKPNGKNNIRYEYDLDHIDCKKNEITFYRHINYSDGSKRKIQYTVGIEGFVDIYDFVNVQKCDAQVYDTKTSKTVGGRKIINSEFAGKTVTTKGGDVRFDSDGFPDFTPYSKKTVRVIGLTGDMANDVPLAMARAKITKYDKSKYVWHHHQDGKTMMLIPKSVHSVRNGGVAHTGGRSVIQHNLLNPNNKLNYSSPEELV>PA2684 MSGLPVSHVGEKVSGGVISTGSPTVHVGSSAVGLADRVSACVPLVGKPVNPMLGSKLLPEEVDFALAAPDTFTFARGYLSSNPRIGRLGRGWWLPGESMHLELSEDACVLVDAQGRRIGFPALAPGAQHYSGSEELWLRRGGSSGGEAQAWRGRWAAVPAELQTQEGSVLVLSGHSYLHFQRCPDGIWRLQASFGRAGYRTEFRWSGRGLLTGVRDSAGRSYALVYQQACEPSEGDDGLRLFGVILASHDGPPPDYIDPQSPGLDWLVRYQFSDSGDLIAVRDRLGQVVRVFAWREHMLVAHGEPGGLEVRYEWDVHAPHGRVVKQIEAGGLTRTFRYLRDATEVSDSLGRVERYEFAGEGGQRRWTALVRADGSRSEFDYDLFGRLVAMRDPLGRETRRRRDGQGRMLEEESPGKARYRKRVDEETGLLVELEDAMQRRWTFERDERGNATTVRGPAGSTRYAYEDPRLPDRPTRIVDPRGGERRLEWNRFGLLAALTDCSGQVWRYDYDNEGRLVASSDPLGQLTRRRYDPLGQLIGLELADGSALSYEYDALGRQTRIADAEGHATLFSWGHGDLLARVSDAGGGELSYLHDEAGRLVALTNENGVQAQFRYDLLDRLVEETGFDGRRQRYRYNAADELIAREDADGRETTYAYDRDGRLASIRVPATEHAPALVERYRWLADGRLASAGGADCEVRYTYDEVGNLRLESQVHADGWVYSVEHSHDALGVRQTSRYGDAPPVAWLTYGPGHLHGALVGAVELAFERDALHREVRRDARRDGQDDALFTQERQHAPLGRLQRSRLRLAGGFDWQRGYRYDGLGQLVGIDDNQYPSVRYEYDLGGRLLASRRAGAAASTYRYDAAGNRLEGVGEHAREDARQAFAENELYRSGFSRSETRASQAGEGPARWAGNRVERIAGNRYRFDALGNLVERIGADGERLRLAYDGAQRLVHLTRDYADGTRLEARYRYDALSRRIAKVVLRDGVEQQVRFGWDGDRQCAEAFARELRTTVHEPGGFVPLLRLEQACEPDPPELLQLRQAFAAEGQPLPAQCVPALGEARIAFFHTDHLGTPLQLSDERGQLRWQGVPDDWRAVAPERQPGAQPIRFQGQYHDEESGLYYNRYRYYLPEAGRYASQDPLGLGGGPNPYAYALNAPTLAYDPTGLIIPLVVIGAFAARAAIGAALGAGIELGMQTGKQVLGQMKDNWDSDRDLTDIKWKCIDINWKHVGASAAIGTVAPGMLSTGKTVVQSAKAIRTLSGQAANTANRAAKLAARKAAHADTIKKAVATQAAWQTGKQIVKCPLKDEEEECPPQ>RhsC6_VA7868 MAKKKLGKAVDWVKKIEEIIKIAQKDTKKYMPELKKAVTKGEGSFNKARKKKQKFGKKNKTKLTKEATSISRNGKSKTRLKITNNKKTKKLKRKGNTRNKKKTNKEKNICINGCPVSMVSGEELLTIEDVILPGAVPFTFKRTYRTSACEVKSYLGYGWSHTLSHQLEFTGDEVLWYDNENRVTSLLLPDEAISESTNPIAGATLYLSDKAGEFVLSAPELPFLHFKRQGNTGHLVRLSDNYNNELFIQYNQNQQPQAVVNPHGIALWLNYNEADLISSIELKTFSDTPDGREWQTQRLMHQYDYNESSQLISEQNEAGEGEYYHYNEQHVITGRQMAGGMTFGWEWEGESKDVRCTKHWSNTGFQASYLWDDEAHTVTVSYPDGSTEVYQHDEETNLINTTDPDGAVTTQEYNDDGQLVRSVDALGYETLHYYDDEGQRELTVLPDGTEISFEYLLGHLFKVTCGDATWRYFYNLEGDLTEKRDPLHQTTFYHYNPQGNLSRIAYPDGSEHQLSWNRLGMLIEERYPDGTTSQYRHDISGRVIYQKSSIGGVTQYQWDEADRLVKLIQPNGKSKTFCYNAYGKVTEVMDEAGRKTAYEYEENSHLLSRVTNPDGTSLSYLYDNPKRFVSQITNERGEAYNIDYFTNGLVKTETTFDGRQMHYAYDLLGQLLKKTEVGSEGTELVTEFTHDEMGRLSEKVLPDGNKVAYGYDESGNLASIDDGETPLAWKYDLLNRVTEEHQNWASNYFEYDVVGQVVKWQLPDAKVLHYQRGQGGLLNQISLDDHVLTRHLYQNGIESRRTQGAVISSFDYDTQGRLVHQSQSINGHQTRTRDYGYDALGNLSQIADSRFGDSYFDYDPLSRLKAVRGNLEEHFVHDATGNVLSQHLGRRQDELNLAEAGGNQLTFHGDSHYEYDEFGRLMTEKRGKNQSLITTYEYDCQHRLIQASMPDGTTATYTYDAFGRRTKKVVTDKAQTTTTTEFIWQGDNLIGEVTGSDYKTYVYEPGTFRPLAQISGEGRNNGEVYYYHLDQIGTPIELTDVQGQSVWSVQYRAYGNVLTQHTEEIQSALRFQGQYYDSETGLHYNRHRYYSPGTGRFTTIDPIGLAGGLNNYQYVPNPTGWVDPLGLYVIKGSQCQDSAARRAKIIDAIEEKMQPVIDYIHKLDPDAQVGFRGSVASGMKGEHKRFGGPDGDRVPFDGEYTHKNKVAVPHNGPQGYDVDLFIVSDKLYSKFPKIPFFKDLSNIDDNIDDILESVRQSLNNNPALKGLKQEKMDIRVWRGKDIIKKLNKGDTQVYLESKIKPQK>RhsA_Dda3937MLNDILSRVARVGAMHAGNRPNPPDDRPQPCRGKPPTSPGKTIKHKSFLGALAGAVAGALVAAAVAAAAVFLVGVTGGLAVAAVGALAVFAAGDLISAVTNKVSAMVDSASPAFGPVASGSGNVFVEKQPVARATKDTVACTKHNSPQLIAQGSESVFVNDAPAARIDDKTVCGATVKEGASTVFFGSGQGTYLDIADEFSWWEKALLIAVEFLVPPSRGMLKGLGKLFIRGPKAVLRGSRAGAKWIAGRLADKSSCASKAFKASSGLTRAKAAVKAFLKDPVYIASGEVIESRTDIELGQTLPLAFERTYRSASVHIGLLGRGWHDSWSEVATVTRDGLNTHVVITLAQGYDIDFTFHQDVQAVYCPHYPEFTLHRRGDGFSLWHRDQQTWRDFSVVQGERRLLSAIHDSHDNRIELVRDPKGYLRQVRHSDGVTLLLVWQGEFLHQIQRIDGGQKTLLAEYRQDEQGRLVEANATQAYHLYYDYDAAHRLTRWHDNDQTWARYEYDAQGRCVYTTCADGFLTARFDYLPDRVVMTDGLGQCSEFGFNDLFLMSWEKSPLGHVTRYEYDDYGNLLREISPAGRVVEFTYLDDTGRVSTFTDASGHQWQYDYDAAQRLCGVTDPLGREWGWMYDAEGNPERLTGPDASEVRFTWNRYGLLTQVSDAAGEVQARLQYDHRQRLLSATDAESRTRQLRYDGQDRVVQWQRADGARFRLGYRRASWTLPEQLIRPDDKEEQRQYDRHNNLLSYVDGNGALWRQTFGPFDLLTARTDAEGRTWHYAYDKESQQLTTVIAPDGSHWQWWLDADGRVIRERDMTGTETHYDYDEDGLCIRVRNGEGDTRHFLYDARGLLLRETAPDDTLHYRYDAVGRLTEVSSSTAHVQLEYDLRDRMVREWHNGTLLTRQVDDAARTVTRTLTWDGDADDTINALAPLTSLFHYTRTGELRQVQLPDGADLTLTHDAAGRESHRTGGSGFVQQREYDVMGWLTREMSGAQHDGHLLATQTREYRYDGAGNLTGVRHNRDAEGYRLDATGRVQEILSGGAGKPVDTTARFLYTRTGLPQEAGRLTEWQAGRLVQHDDTHYQYDRAGRLIRKQVVQPGYRPQVWQYRWDSRNQLRVVDTPNGERWLYRYDPFGRRVGKRCDQKAEEIRYLWDGDQIAEIRHYRHGQLIQRRHWVYNGWELVVQQRQHTGGDWETDFVTSSQNGTPQALFTPDGTLRWQAPKATLWGQRQAEKSESPDPGLAFAGQLRDSESGLCYNRFRYYDPAGGCYVSPDPIGIAGGESNYGYVSNPMCWVDPFGLAKCPTLAHGANGEILSAKATVSKAELRTGSGTNQSSRDYARSLGNQTDDAGHILGNVLGGQGGKGNVFPQLPAINRGQYRDFEKVVKDYIGQHGSVDIEWAFKYGNGGTRPTEIYYDVYQNGQKVFGRIFNN>RhsB_Dda3937MLNDILSRVARVGAMHAGNRPNPPADRPQPCQGKPPTSPGKTIKHKSFLGALAGAVAGALVAAAVAAAAVFLVGVTGGLAVAAVGALAVFAAGDLISAVTNKVSAVVDSASPAFGPVASGSGNVFVEKQPVARATKDTVACTKHNSPQLIAQGSESVFVNDAPAARIDDKTVCGATLKEGASTVFFGSGQGTYLEIADEFSWWEKALLIAVEFLVPPSRGMLKGLGKLFTRNGLKSVLKGAKAGALFITKVPGKMGCAARAFKANKGMARFKEAAKAFKKDPVYLASGEVIESRTDIELGQTLPLVFERTYRSASAHTGLLGRGWHDSWSEVATVTHDGLNTHVVITLAQGYDIDFTFHQDVQAVYCPHYPEFTLHRRGDGFSLWHRDQQTWRDFSVVQGERRLLSAIHDSHDNRIELVRDPKGYLRQLRHSDGVTLLLVWQGEYLHQIQRIDGGQKTLLAEYRQDEQGRLVEANATHAYHLYYEYNTAHRLTRWHDNDQTWARYEYDAQGRCVYTTCADGFLTARFDYLPDRVVMTDGLGQRSEFGFNDLHLMSWEQSPLGHITRYEYDEVGNLLREISPAGRVVEFTYLDDTGRVSTFTDGSGHQWQYDYDDAQRLCGVTDPLGREWGWVYDAEGNPERLTGPDASEVRFTWNRYGLLTQVSDAAGEVQARLQYDHRQRLLSATDAESRTRQLRYDRQDRVVQWQRADGARFRLGYRRASWTLPEQLIRPDDKEEQRQYDRHNNLLSYVDGNGALWRQTFGPFDLLTARTDAEGRTWRYEYDRESQQLIAVTAPDGSRWQWWLDADGRVIRERDMTGTETHYGYDEDGLCIRVRNGEGDTRHFLYDARGLLLRETAPDDTLHYRYDAAGRLTEVSSATAHVQLDYDLRDRVVREWHNGTLLTRQYDDAARTVTRTLTWDGDADDTTGTLAPLTSLFHYTRTGELRQVQLPDGADLTLTHDAAGRESLRTGGSGFVQQREYDVMGWLTREQSGAQHDGRLQPAQTREYRYDGAGNLTGVRHNRDAEGYRLDATGRVQEMLSGGAGKPVDTTARFHYTRTGLPQEAGRLTEWQAGRLVQHDDTHYQYDRAGRLIRKQVVQPGYRPQVWQYRWDSRNQLRVVDTPNGERWLYRYDPFGRRVGKRCDQKAEETRYLWDGDQIAEIRHYRHGQLIQRRHWVYNGWELVVQQRQHTGGDWETDFVTSSQNGTPQALFTPDGTLRWQVPKATLWGQRQTEKSESPDPGLAFAGQLRDSESGLCYNRFRYYDPAGGCYVSPDPIGIAGGESNYGYVQNPNTRVDPLGLAGCAMGEILADADKWSLAKIGDRQKGMIKDKLSTVKERSKALNTKMREHFNANEQKIISEWEKQTGMNWPTLSSGSRATPHHVIPIKNGGSNEWWNIIPVQHPHTGTIHGTGSALRTHLPYQKDGGKLWNLLGY>RhsA1_PWN146MTEAARVGDIIGHSHALAGMIAGTLVGGLIAAAGAVAAGALFVAGLAASCIGVGVLLIGASLAVGYLTGEAATAARDGIADAGAGSLTPKGNIVTGSPNVFINGKPAALATHSQVACSDDGPSMQMAQGSDKVSINGQPASRVGDKTNCDAQVMEGSPNVFIGGGTVTTLPIKPEVPDWLYKVSDLTLLFAGLVGGVGGAAGKLGALGKLLGKLPGINKLARIACRAGTLMTATAAVGIIARPVDIVSGQKFLDGEDDLDFVLPSRLPVAWQRYWRSGNPGDSVLGRGWNLFWESSLQPYQDGLVWRAPSGDFVAFPMVPRGHKTYCEAEKCWLMHNDDGSWQLFDVGEQIFHYPPLAGEQPSRLSMITDPIGNATSLFYDDEGLLSELVDSAGQRLMCRYAQGRLREVALQTAEGERTLARYGYDEQGQLTTVSNRAGEVTRRFGWRDGLMISHQDAAGLLNEYQWQEIDGVPRVTAYRNSAGESLEFGYDFAGGRRSAVRGDGKRAEWRLDDDDNVAQYTDFDQRRYGFIYQRGELCSVLLPGGAQRQSEWDSYGRLLSETDPLGRVTRYQYSRNSGRLFAVAYPDGSSEAQHWDTLGRPTRYVDALGNTTLYRYPDDEESLPASVIDALGGEVKLEWDARGQLTRYTDCSGSVTAYTYDARGQLTAQTDAEGHQTRYLWDNGGRLHTLIHPDGGEERFNWNAHGQLAEHQDALGSLTRWQYNALGLPVSITDRINRTRRYHYSPQGWLTRLENGNGGDYRFSYDAVGRVLAEERPDDTRHYYRYGAAGLLEEHREVGLPGSAGELTQREQRFRFDEAGQLVWRGNASAEWHYRFDAMGRLRELNRLPTASGAALGIEPDSVQMRYDAAGRLLGEQGVNGELQYQWDALANLQALTLPQGDRLQWLYYGSGHASAIKFNQQVVSEFTRDRLHRETGRSQGALQQQRRYDAMGRRSWQSSAFGHDKLTRPEDGVLWRAYRYTGRGELAGVSDALRGEVHYGYDAEGRLLQHREPNQGKPGARLVYDLADNLLGERSPQSDIDAHLPLAPIADNRLTHWQKLFYRYDAWGNLISRRNGLYEQHYRYDADNRLVQAHGRGPQGEFEAQYHYDALGRRSRKAVRYKGKTEQTTRFLWQGYRLLQEQRDDGSRRSWSYDPASPWSPLAALEQAGDSRSADIYWYHTDLNSAPLEVTDAAGNLCWSGQYDTFGKLQGQTVAGAAKRQGAQYQQPLRYAGQYQDDESGLHYNLFRYYEPEVGRFTTQDPIGLRGGLNLYQYAPNPLMWVDPFGLSGTRIPRTNGHWDGVPGESNWYSYKPAVNAVTNNQPIPFSNGRPDFSPWSKGSITFENGVLDGTEKDFPKVYKAIAKEKGITQSAAQKLLRKNKLTPHHLSNTEIILVPRGLHGNVPHIGSASDMRNGKKC>RhsC2_PWN146 MYDLADNLLGERSPQSDIDAHLPLAPIADNRLTHWQKLFYRYDAWGNLISRRNGLYEQYYRYDADNRLVQAHGRGPQGEFEAQYHYDALGRRSRKAVRYKGKTEQTTRFLWQGYRLLQEQRDDGSRRSWSYDPASPWSPLAALEQAGDSRSADIYWYHTDLNSAPLEVTDAAGNLCWSGQYDTFGKLQGQTVAGAAKRQGAQYQQPLRYAGQYQDDESGLHYNLFRYYEPEVGRFTTQDPIGLRGGLNLYQYAPNPLMWVDPFGLSTRRDQLPGDKTLETSRAARREAMRSQGIPTSQTYQVQVVEDITQSENSKLNSGILHEERIMRNNSEVGRVKVHHQGHVFTDNDTFEKPHYHGTKGEHFSYEKGRPRNTNKYKGDRCF>RhsA_Z5014 MSGKPAARQGDMTQYGGSIVQGSAGVRIGAPTGVACSVCPGGVTSGHPVNPLLGAKVLPGETDIALPGPLPFILSRTYSSYRTKTPAPVGSLGPGWKMPADIRLQLRDNTLILSDNGGRSLYFEHLFPGEDGYSRSESLWLVRGGVAKLDEGHRLAALWQALPEELRLSPHRYLATNSPQGPWWLLGWCERVPEADEVLPAPLPPYRVLTGLVDRFGRTQTFHREAAGEFSGEITGVTDGAGRHFRLVLTTQAQRAEEARQQAISGGTEPSAFPDTLPGYTEYGRDNGIRLSAVWLTHDPEYPENLPAAPLVRYGWTPRGELAVVYDRSGKQVRSFTYDDKYRGRMVAHRHTGRPEIRYRYDSDGRVTEQLNPAGLSYTYQYEKDRITITDSLNRREVLHTQGEGGLKRVVKKEHADGSVTQSQFDAVGRLRAQTDAAGRTTEYSPDVVTGLITRITTPDGRASAFYYNHHSQLTSATGPDGLEIRREYDEWGRLIQETAPDGDITRYRYDNPHSDLPCATEDATGSRKTMTWSRYGQLLSFTDCSGYVTRYDHDRFGQMTAVHREEGLSQYRAYDSRGQLIAVKDTQGHETRYEYNAAGDLTTVIAPDGSRNGTQYDAWGKAICTTQGGLTRSMEYDAAGRVIRLTSENGSHTTFRYDVLDRLIQETGFDGRTQRYHHDLTGKLIRSEDEGLVTHWHYDEADRLTHRTVKGETAERWQYDERGWLTDISHISEGHRVTVHYGYDEKGRLTGERQTVHHPQTEALLWQHETRHAYNAQGLANRCIPDSLPAVEWLTYGSGWLAGMKLGDTPLVDFTRDRLHRKTLRRFGRYELTTAYTPAGQLQSQHLNSLQYDRDYTWNDNGELIRISSPRQTRSYSYSDSGRLTGVHTTAANLDIRIPYATDPAGNRLPDPELHPDSTLSMWPDNRIARDAHYLYRYDRHGRLTEKTDLIPEGVIRTDDERTHRYHYDSQHRLVHYTRTQYEEPLVESRYLYDPLGRRVAKRVWRRERDLTGWMSLSRKPQVTWYGWDGDRLTTIQNDRTRIQTIYQPGSFTPLIRVETATGELAKTQRRSLADALQQSGGEDGGSVVFPPVLVQMLDRLESEILADRVSEESRRWLASCGLTVAQMQSQMDPVYTPARKIHLYHCDHRGLPLALISAEGATEWCAEYDEWGNLLNEENPHQLQQLIRLPGQQYDEESGLYYNRHRYYDPLHGRYITHDPIGLKGGWNFYQYPLNPVINVIRKLVDVFPRPFPLPIPWPKSPAQQQADDNAAKALTKWWNDTASQRIFDSLILNNPGLALDITMIASRGNVADTGITDRVNDIINDRFWSDGKKPDRCDVLQELIDCGDISAKDAKSTQKAWNCRHSRQSNDKKR>Ct1C_CRM83 MSEGPGGPQGATAGGTLAMRMLSQQAMVASQMKRAANDKAIAQMLASKKSGPPAARLGDEIQHKSFLGALAGAVLGAIVTIAEGCLIMAACATGPYALVLVPALMYASYKASDYVEEKQNQLESWINSFCDTDGAINTGSENVKINGELAARAAVTLPPPPPPGAIPEVPQGEPSWGDIATDLLESAAEKAVPLAKAWGNAVITLTDSNAGFMDRVSAGASLLFPAGPVLMEFATMVGGRGEIKKEVDFPEAGEDTALCDKENKPPRIAQGSSNVFINNQPAARKGDKLECSAAIVGGSPDVFIGGEQVTYLDIQPEFPPWQRMILGGITIASYLLPPAGLLGKLKNLARLGKLGNLLGKSGKLLGAKLGALLGKTGKSLKSIANKVIRWVTDPVDPVTGAYCDERTDFTLGQTLPLSFTRFHSSVLPLHGLTGVGWSDSWSEYAWVREQGNRVDIISQGATLRFAFDGDSDTTVNPYHAQYILRRRDDYLELFDRDALSSRFFYDAFPGMRLRHPVTDDTSDDRLAHSPNDRMYMLGGMSDTASNRITFERDSQYRITGVSHTDGIRLKLTYHASGYLKAIHRTDNGIQTLATYEQDARGRLTEADARLDYHLFYEYDAADRIIRWSDNDQTWSRFTYDAQGRCVTVTGAEGYYNATLDYGDGCTTVTDGKGTHRYYYDPDGNILREAAPDGSTTTYEWDEFHHLLARHSPAGRVEKFEYNAALGQLSRYTAADGAEWLYRYDERGLLSNITDPAGQTWTQQCDERGLPVSLVSPQGEETRLAYTAQGLLSGIFRQDERRLGIEYDHHNRPETLTDVMGREHHTEYSGHDLPVKMRGPGGQSVRLQWQQHHKLSGIERAGTGAEGFSYDRHGNLLAWTDGNGVVWTMEYGPFDLPVARTDGEGHRWQYRYDKDTLQLTEVINPQGESYRYILDNCGRVTEERDWGGVVWRYRYDADGLCTARVNGLEETILYSRDAAGRLAEIITPEGKTQYAYDKSGRLTGIFSPDGISQRTGYDERGRVNVTTQGRRTIEYHYPDEHTVIRCILPPEDERDRHPDESLLKTTYRYNAAGELTEVILPGDETLTFSRDEAGREVLRHSNRGFACEQGWNAAGQLTSQRAGLFPAEATWGGLLPSLVREYRYDSAGNVSGVTSREDYGRETRREYRLDRNGQVTAVTASGTGLGYGEGDESYGYDSCGYLKAQSAGRHRISEETERYAGGHRLKQAGNTQYDYDAAGRMVSRTKHRDGYRPETERFRWDSRDQLTGYCSAQGEQWEYRHDASGRRTEKRCDRKKIRFTYLWDGDSIAEIREYRDDKLYSVRHLVFNGFELISQQFSRVRQPHPSVAPQWVTRTNHAVSDLTGRPLMLFNSEGKTVWRPGQTSLWGLALSLPADTGYPDPRGELDPEADPGLLYAGQWQDAESGLCYNRFRYYEPETGMYLVSDPLGLLGGEQTYRYVPNPCGWVDPLGLAASSKISSLMDYIGDGRRVSGHTGFLDGVRLSRSQINNIAKEMEKLGIKVIRKADKYLPPNARAAFDYGLRNIYLRKNATLYEVYHEVIHAKQFAKIGREAYEALGRLSREEHVLNEILKSKNLFNEAEIAHAIKYVEGLREKFMMGLTN>TccC3MKNIDPKLYQKTPTVSVYDNRGLIIRNIDFHRTTANGDPDTRITRHQYDIHGHLNQSIDPRLYEAKQTNNTIKPNFLWQYDLTGNPLCTESIDAGRTVTLNDIEGRPLLTVTATGVIQTRQYETSSLPGRLLSVAEQTPEEKTSRITERLIWAGNTEAEKDHNLAGQCVRHYDTAGVTRLESLSLTGTVLSQSSQLLIDTQEANWTGDNETVWQNMLADDIYTTLSTFDATGALLTQTDAKGNIQRLAYDVAGQLNGSWLTLKGQTEQVIIKSLTYSAAGQKLREEHGNDVITEYSYEPETQRLIGIKTRRPSDTKVLQDLRYEYDPVGNVISIRNDAEATRFWHNQKVMPENTYTYDSLYQLISATGREMANIGQQSHQFPSPALPSDNNTYTNYTRTYTYDRGGNLTKIQHSSPATQNNYTTNITVSNRSNRAVLSTLTEDPAQVDALFDAGGHQNTLISGQNLNWNTRGELQQVTLVKRDKGANDDREWYRYSGDGRRMLKINEQQASNNAQTQRVTYLPNLELRLTQNSTATTEDLQVITVGEAGRAQVRVLHWESGKPEDIDNNQLRYSYDNLIGSSQLELDSEGQIISEEEYYPYGGTALWAARNQTEASYKTIRYSGKERDATGLYYYGYRYYQPWIGRWLSSDPAGTIDGLNLYRMVRNNPVTLLDPDGLMPTIAERIAALKKNKVTDSAPSPANATNVAINIRPPVAPKPSLPKASTSSQPTTHPIGAANIKPTTSGSSIVAPLSPVGNKSTSEISLPESAQSSSSSTTSTNLQKKSFTLYRADNRSFEEMQSKFPEGFKAWTPLDTKMARQFASIFIGQKDTSNLPKETVKNISTWGAKPKLKDLSNYIKYTKDKSTVWVSTAINTEAGGQSSGAPLHKIDMDLYEFAIDGQKLNPLPEGRTKNMVPSLLLDTPQIETSSIIALNHGPVNDAEISFLTTIPLKNVKPHKR>WapA MKKRKRRNFKRFIAAFLVLALMISLVPADVLAKSTEEENGNRIAADDPEETLQKEQTEEAVPFDPKDINKEGEITSERTENTKLYYEGDGVYKQEVYLDPIHTKETPDADWEDISPELKESTSKQVETENAILNSDFQKQMKNGLYATFEHNDHKVTYSLAEAKGPNKTSLTPKDTSADYKTDSNEIVYPDVFPNIDLQTFTFNENIKEDLVLHQYNGYNTFTFQLKTDLQAKEQEDGSIDFSDEKGKVVFSVPKPFMTDSKLDELSGEVERSDKVSYKLEKNEEGYLLHLTADENWLKDPERVYPVSIDPSTSLSVSSDTFVMSAYPTTNYSASSQKWDANLKAYVLKTGYYDKTTGTNYAFMKFNNLKPIQNMTVTKATLKTYVAHSYYGTKATGLWLDTVNSNYDNAKVTWNTKPASKNIGKADVHKGQWASYDVTAAVKSWNSGGANYGFKLHTNGNGKEYWKKLISSANSANKPYIEVTYTIPKGNTPTIKAYHNGDSTGYFDISWKKVEGAKGYKVWIYNGKEYQAISAGNVTSWSTKGKKIWPTSAEIASKRYKLHLDGKDGAELALDPSPVYKNSGGSYATSKNYWIGVSAIFDQGEGAMSAPAKPVIPNVGKAQAPSAKGYNNGNATGYFDLSWKAVSGATGYKVQVFNGKGFETLDLGNQTSWTTKGKKIWPTSAEIKAGKYALHLKDGSGAELPINPGPTYKNAGGDGAKRNYSFKIIAYNKDGEAIASPAATPALPDIARPKNVTGYLYTNTKSSQTGYVNLIWEKVQNAKGYKVNIYNGKEYQSFDVGDADHWTTQNKNIWPTSEEIKAGSYKLHTDGKGGELALDPSPVYNNANGNYKGKKNYSFTLVAYDANGETIPTAPFNPTFHEGAEFLGTEEYWSIIDIPSGQLNGATGNVIVNEEDLSIDGRGPGLGLSRTYNSLDSSDHLFGQGWYADAETSVISTDGGAMYIDEDATTHRFTKKADGTYQPPTGVYLELTETADQFILKTKDQTNAYFNKKGGKLQKVVDGHNNATVYTYNDKNQLTAITDASGRKLTFTYDENGHVTSITGPKNKKVTYSYENDLLKKVTDTDGTVTSYDYDSEGRLVKQYSANSTEAKPVFTEYQYSGHRLEKAINAKKETYVYSYDADKKTLLMTQPNGRKVQYGYNEAGNPIQVIDDAEGLKITTNTKYEGNNVVEDVDPNDVGTGKATESYQYDKDGNVTSVKDAYGTETYEYNKNNDVTKMKDTEGNVTDIAYDGLDAVSETDQSGKSSSAAVYDKYGNQIQSSKDLSASTNILKDGSFEAQKSGWNLTASKDSGKISVIADKSGVLSGSKALEVLSQSTSAGTDHGYSSATQTVELEPNTTYTLSGKIKTDLAKSRAYFNIDLRDKDQKRIQWIHNEYSALAGKNDWTKRQITFTTPANAGKAVVYMEVDHKDKDGKGKAWFDEVQLEKGEVSSSYNPVQNSSFTSATENWNVSGASVDSEEGFNDDVSLKAARTSASQAGSVTKQTVVLGQSANDKPVYLTLTGMSKASSVKFTDEKDYSLQANVTYADGSTGIYNAKFPSGTQEWNRAAVVIPKTKPINKVDISILFQKSATGTVWFDDIRLIEGSLLTKSTYDSNGNYVTKEEDELGYATSTDYDETGKKTSETDAKGEKTTYTYDQADQLTNMTLSNGTSILHSYDKEGNEVSKTIRAGADQTYKFEYDVMGKLVKTTDPLGNVLASEYDANSNLTKTISPNGNEVSLSYDGTDRVKSKSYNGTEKYIFTYDKNGNETSVVNKEQNTTKKRTFDNKNRLTELTDRGGSQTWTYPSDSDKLKTFSWIHGDQKGTNQFTYNKLDQMIEMKDSTSSYSFDYDENGNVQTFITGNGGGTSFSYDERNLVSSLHIGDKNGGDILTESYEYDANGNRTTINSSASGKVQYEYGKLNQLVKETHEDGTVIEYTYDGFGNRKTVTTIKDGSSKTVNASFNIMNQLTKVNDESISYDKNGNRTSDGKFTYTWDAEDNLTAVTKKGEDKPFATYKYDEKGNRIQKTVNGKVTNYFYDGDSLNVLYETDADNNVTKSYTYGDSGQLLSYTENGKKYFYHYNAHGDIIAISDSTGKTVAKYQYDAWGNPTKTEASDEVKDNRYRYAGYQYDEETGLYYLMARYYEPRNGVFLSLDPDPGSDGDSLDQNGYAYGNNNPVMNVDPDGHWVWLVVNAGFAAYDGYKAYKSGKGWKGAAWAAASNFGPGKIFKGASRAYKFTKKAVKITGHTRHGLNQSIGRNGGRGVNLRAKLNAVRSPKKVIKQPNGATKYVGKKATVVLNKRGKVITAYGSSRAKGSKHVFHTHGKGNKSKRRR
