# Supplementary material for: Intramolecular chaperone-mediated secretion of an Rhs effector toxin by a type VI secretion system
Source: Nat Commun. 2020 Apr 20;11:1865. doi: 10.1038/s41467-020-15774-z (PMC7170923; doi:10.1038/s41467-020-15774-z)
Supplement: Supplementary file 9 — Reporting Summary [file 41467_2020_15774_MOESM9_ESM.pdf]

nature research

Corresponding author(s): Tao Dong

Last updated by author(s): Mar 23, 2020

Reporting Summary

Nature Research wishes to improve the reproducibility of the work that we publish. This form provides structure for consistency and transparency in reporting. For further information on Nature Research policies, see Authors & Referees and the Editorial Policy Checklist .

Statistics

For all statistical analyses, confirm that the following items are present in the figure legend, table legend, main text, or Methods section.

n/a	Confirmed	
☐	☒	The exact sample size (*n*) for each experimental group/condition, given as a discrete number and unit of measurement
☐	☒	A statement on whether measurements were taken from distinct samples or whether the same sample was measured repeatedly
☐	☒	The statistical test(s) used AND whether they are one- or two-sided<br>*Only common tests should be described solely by name; describe more complex techniques in the Methods section.*
☐	☒	A description of all covariates tested
☐	☒	A description of any assumptions or corrections, such as tests of normality and adjustment for multiple comparisons
☐	☒	A full description of the statistical parameters including central tendency (e.g. means) or other basic estimates (e.g. regression coefficient) AND variation (e.g. standard deviation) or associated estimates of uncertainty (e.g. confidence intervals)
☐	☒	For null hypothesis testing, the test statistic (e.g. *F*, *t*, *r*) with confidence intervals, effect sizes, degrees of freedom and *P* value noted<br>*Give P values as exact values whenever suitable.*
☒	☐	For Bayesian analysis, information on the choice of priors and Markov chain Monte Carlo settings
☒	☐	For hierarchical and complex designs, identification of the appropriate level for tests and full reporting of outcomes
☒	☐	Estimates of effect sizes (e.g. Cohen's *d*, Pearson's *r*), indicating how they were calculated

Our web collection on statistics for biologists contains articles on many of the points above.

Software and code

Policy information about availability of computer code

Data collection	No software was used for data collection.
Data analysis	Commercial and open source programs were used for analysis (as indicated in Methods) including Microsoft Office (2016), Benchling (https://www.benchling.com/), Phyre2 (http://www.sbg.bio.ic.ac.uk/phyre2/html/page.cgi?id=index), Blast(https://blast.ncbi.nlm.nih.gov/Blast.cgi), Chimera(1.13.1), Clustal Omega(https://www.ebi.ac.uk/Tools/msa/clustalo/), WebLogo(2.8.2), IQ-Tree (1.6.8), iTOL(v5)

For manuscripts utilizing custom algorithms or software that are central to the research but not yet described in published literature, software must be made available to editors/reviewers. We strongly encourage code deposition in a community repository (e.g. GitHub). See the Nature Research guidelines for submitting code & software for further information.

Data

Policy information about availability of data

All manuscripts must include a data availability statement. This statement should provide the following information, where applicable:

- Accession codes, unique identifiers, or web links for publicly available datasets
- A list of figures that have associated raw data
- A description of any restrictions on data availability

The data that support the findings of this study are available within the paper or available from the corresponding author upon reasonable request. The source data underlying Fig. 1B, 1D, 2C–D, 3A-F, 4A–I, 5E and Supplementary Fig. 2B, 6B-E, 7A-B, and 7D are provided as a Source Data file.

Field-specific reporting

Please select the one below that is the best fit for your research. If you are not sure, read the appropriate sections before making your selection.

[x] Life sciences [ ] Behavioural & social sciences [ ] Ecological, evolutionary & environmental sciences

For a reference copy of the document with all sections, see nature.com/documents/nr-reporting-summary-flat.pdf

Life sciences study design

All studies must disclose on these points even when the disclosure is negative.

Sample size	We used at least 3 biological replicates for all experiments. We chose this sample size as it is standard practice for most microbiological assays.
Data exclusions	No data were excluded from analysis.
Replication	All results reported in the paper were reliably reproduced in at least two independent experiments.
Randomization	Not applicable to this research as it employed bacterial strains rather than placebo-controlled patient groups.
Blinding	Not applicable to this research as it employed bacterial strains rather than placebo-controlled patient groups.

Reporting for specific materials, systems and methods

We require information from authors about some types of materials, experimental systems and methods used in many studies. Here, indicate whether each material, system or method listed is relevant to your study. If you are not sure if a list item applies to your research, read the appropriate section before selecting a response.

Materials & experimental systems

n/a	Involved in the study	
[ ]	[x]	Antibodies
[x]	[ ]	Eukaryotic cell lines
[x]	[ ]	Palaeontology
[x]	[ ]	Animals and other organisms
[x]	[ ]	Human research participants
[x]	[ ]	Clinical data

Methods

n/a	Involved in the study	
[x]	[ ]	ChIP-seq
[x]	[ ]	Flow cytometry
[x]	[ ]	MRI-based neuroimaging

Antibodies

Antibodies used	Monoclonal antibodies were purchased from Sigma Aldrich (FLAG # F1804 and 6His # SAB4600386), Thermo Scientific (V5 # 37-7500), and Biolegend (RpoB # 663905). The polyclonal antibodies to Hcp, the VIRN and the Rhs domains of TseI were custom-made by Shanghai Youlong Biotech. The secondary antibodies (anti-mouse # 7076S or anti-rabbit # 7074S IgG HRP linked) were purchased from Cell Signaling Technology (CST) .
Validation	Commercial antibodies have been validated by the manufacturers as described on the websites following the links below:<br>RpoB: https://www.biolegend.com/en-us/products/purified-anti-e-coli-rna-polymerase-beta-antibody-12495<br>FLAG: https://www.sigmaaldrich.com/catalog/product/sigma/f1804?lang=en&region=CA<br>6His: https://www.sigmaaldrich.com/catalog/product/sigma/sab4600386?lang=en&region=CA<br>V5: https://www.thermofisher.com/cn/en/antibody/product/V5-Tag-Antibody-clone-2F11F7-Monoclonal/37-7500<br>The polyclonal antibodies to Hcp, the VIRN and the Rhs domains of TseI were validated in this study and shown in the Supplementary figure.
